# Does emotion regulation engage the same neural circuit as working memory? A meta-analytical comparison between cognitive reappraisal of negative emotion and 2-back working memory task

**DOI:** 10.1371/journal.pone.0203753

**Published:** 2018-09-13

**Authors:** Tien-Wen Lee, Shao-Wei Xue

**Affiliations:** 1 Center for Cognition and Brain Disorders, Hangzhou Normal University, Hangzhou, China; 2 Zhejiang Key Laboratory for Research in Assessment of Cognitive Impairments, Hangzhou, China; 3 Department of Psychiatry, Dajia Lee's General Hospital, Lee's Medical Corporation, Taichung, Taiwan; University of Graz, AUSTRIA

## Abstract

Research into cognitive emotion regulation (ER) extends our understanding of human cognition, which is capable of processing objective information and is crucial in maintaining subjective/internal homeostasis. Among various ER strategies, the alleviation of negative emotion via reappraisal is of particular importance for adaptation and psychological well-being. Although still debated, previous neuroimaging studies tend to infer that the reappraisal ER is mediated by the capability of working memory (WM), which has not been examined empirically. This meta-analytical study of published neuroimaging literature used activation likelihood estimation (ALE) to compare the neural circuits that regulate negative emotion (reappraisal tasks; 46 studies/1254 subjects) and execute WM (2-back tasks; 50 studies/1312 subjects), with special emphasis on the prefrontal cortex (PFC). Taking the canonical WM network as a reference, ALE results revealed that the dorsal midline PFC was partly shared by both ER and WM, whereas ER-specific PFC structures were delineated in the inferior, middle, and superior frontal cortices, as well as in the posterior brain regions. The peak coordinates of ER in the middle frontal cortex were dorsal to those of WM by 15.1 mm (left) and 21.6 mm (right). The results support specialized emotion-related neural substrates in the PFC, negating the assumption that reappraisal ER and WM rely on the same neural resources. The holistic picture of "emotional brain" may need to incorporate the emotion-related PFC circuit, together with subcortical and limbic emotion centers.

## Introduction

Emotions describe multi-faceted, whole-body phenomena that involve loosely coupled changes in the domains of subjective experience, behavior, and central as well as peripheral physiology [1, 2]. Human emotion can be driven by external or internal factors, e.g., events that threaten or reward, cognitive processes such as appraisal, expectation or imagination or changes in psychophysiological states [3, 4]. The elicited emotional response comprises a broad set of mental and physiological processes, including (not exclusively) multi-level evaluation, system regulation, motivation, action preparation (e.g., muscle-tone and energy level), communication (e.g., tone of voice and facial expressions) and monitoring [5, 6]. In addition, emotions may impose considerable influence on an organism by enhancing, distorting, or suppressing other mental constructs [7]. Conversely, emotions themselves are subject to regulation to optimize adaptation, termed emotion regulation (ER). ER may diminish or augment an emotional response in either amplitude or duration [8]. According to the process model proposed by Gross [9], ER strategies can be largely categorized into antecedent- and response-focused schemes; the former includes situation selection, situation modification, attention deployment, and cognition changes (reappraisal), while the latter modifies experiential, behavioral, or physiological responses. Recent studies have scrutinized various self-regulatory mechanisms and have substantially expanded the repertoire of ER research [10, 11]. It has been suggested that an individual's habitual use of ER strategies may affect psychological well-being [12]. Impaired ER has been implicated in a wide range of psychiatric conditions, especially depression and anxiety disorders [13–15].

Antecedent-focused ER has been explored by various paradigms, such as reinterpretation, distancing, imagination, counteraction by recalling memory; these ER tactics generally demand the participation of cognitive reappraisal (note: antecedent-focused ER via thought suppression and distraction may not be mediated by reappraisal) [16–25]. In adults, reappraisal is one of the most commonly used ER strategies, and greater reappraisal use is associated with greater positive affect, greater well-being, diminished negative affect and fewer depressive symptoms [12]. Although the tactics of reappraisal are quite diverse, several mental operations are indispensable and shared: keeping the tactic in mind to exert its influence; resolving/monitoring the conflict between habitual and target reactions; selection among possible alternatives; and the modulation of behavior [26]. Previous researchers have addressed similarities in the psychological processes involved in the cognitive reappraisal of emotion and working memory (WM) (and several orchestrated mental processes, described below) [1, 17, 26–28]. WM is a member of the executive function set that enables us to hold information temporarily and manipulate the content online to execute complex cognitive tasks, which usually requires overriding habitual responses (e.g., resolution of the conflict between upcoming and already registered information) [29, 30]. Similar to ER in that it may attenuate or augment the impact of induced emotion, WM function is also adaptive since it may sustain goal-directed behavior and free us from distractions and habitual responses. In experimental conditions where the tactic of reappraisal is pre-determined, reappraisal ER involves keeping the goal to reappraise in WM (either up- or downregulation of emotions), maintaining the selected appraisal in WM, modifying/adjusting the situation online, and finally monitoring the extent to which one is successful in changing the affective state [1, 31]. This has led to the conceptualization that reappraisal ER may depend on well-studied cognitive abilities, such as working memory, attention, response selection and outcome monitoring, that engage lateral prefrontal-parietal regions and the anterior cingulate cortex (ACC) [28, 31, 32]. Concordant with the above inferences, a wealth of neuroimaging studies have consistently disclosed that ER and WM engage proximal neural substrates in the prefrontal cortex (PFC) and midline PFC structures [33–36]. Specifically, previous meta-analyses have shown the ER network involves the bilateral superior, middle and inferior frontal cortex (SFC, MFC, and IFC), dorsal medial PFC (dmPFC, which may extend to the ACC), left middle temporal gyrus, and left angular gyrus [33, 34], whereas the WM network comprises the bilateral MFC, pre-supplementary motor area (pre-SMA, situated at the dorsomedial part of the PFC), ACC, bilateral insula (extending to the adjacent IFC), bilateral inferior parietal lobule (extending to the superior parietal lobule), and thalamus [35, 36]. The MFC is believed to be the neural substrate that actively maintains information, while the IFC may be related to response selection/inhibition and regulatory control [37–40]. The adjacent ACC, pre-SMA, and dmPFC may be involved in conflict resolution (between top-down and bottom-up competition) and self-monitoring processes [26, 41, 42]. The issue of whether WM and ER recruit the same neural correlates, although advocated by previous research, has never been examined by rigorous statistical examination.

The theoretical implication of the ER–WM resemblance is profound. Although emotion and cognition used to be regarded as separate entities, recent progress in affective neuroscience has persuasively challenged the dichotomous notion and has claimed that they are actually embodied in conjoined and highly interactive brain regions [43]. The relationship between emotion and cognition is bi-directional and could be far more intimate than previously thought. For example, emotion may grab attention, bias cognition, alter memory, affect perception, and modulate behavior [43]. Conversely, cognition may precede and evoke emotion (refer to appraisal theory) and exercise top-down control over emotion. In addition, the neural structures conventionally viewed as affective, e.g., the amygdala, are also cognitive (such as in decision-making), and the neural structures ordinarily presumed to be cognitive, e.g., the lateral PFC, also carry affective function (such as in ER). Whether ER and WM rely on the same neural substrates is thus an issue of paramount importance in affective neuroscience. Despite the apparent validity, our daily experience does assure that cognition can work independent of emotion, and sometimes we fall prey to emotions when our cognition seems unable to prevent us from regrettable decisions. Careful inspection of previous meta-analyses reveals that in the inferior lateral forebrain, the major activation foci of ER and WM appear to be close but distinct, with the former and the latter being situated in the lateral (IFC) and medial (anterior insula) sectors, respectively [33–36]. In addition, the reported SFC foci of ER (e.g., Talairach coordinates [–14 42 40] in [33]), which used to be attributed to the neural correlates of attention-related process, is actually positioned anterior to the supplementary eye field (e.g., Talairach coordinates [0 0 52] in [44]). Importantly, recent evidence suggests that only the practice of emotional WM, not standard WM, may benefit affective control [45]. This intriguing result implies that emotion-related cognition (hot cognition) and its non-emotional counterpart (cold cognition) are, at least partly, separable and non-transferable. Altogether, the dissociation of ER and WM is implicated from both neuroscientific and psychological perspectives. To reconcile the discrepancy that ER and WM appear similar but dissociable (both neurally and psychologically) [1, 17, 26–28, 33–36], the authors hypothesized that ER may be processed by a specialized circuit in the PFC, which accounts for its uniqueness (e.g., [45]) and closeness to WM (e.g., [43]). To be clear, although the neural substrates of ER and WM appear proximal to each other at first glance, these functions might be supported by distinct and neighboring neural resources.

This study, accordingly, aims to investigate whether the neural substrates of WM and ER are indistinguishable in the three sectors of the PFC (IFC, MFC and SFC). Both ER and WM have been explored extensively in neuroimaging literature and there have been many modified experimental designs. Given that this study attempted to differentiate the neural correlates of ER and WM, which are observed to be proximal in the frontal region, meta-analyses (activation likelihood estimation; ALE) were employed to extract representative brain maps, and, thus, facilitate direct comparisons [46, 47]. Among the various reappraisal tactics of ER, only reinterpretation and distancing to downregulate negative emotion were incorporated into the analysis because (1) decreasing the duration or intensity of negative emotions appears to be of particular importance in daily life [48], (2) most ER research has been performed on the regulation of negative emotions, (3) reinterpretation and distancing are the most commonly applied paradigms (our literature search supports this impression, see Results) and arguably also the most frequently used reappraisal method for coping with negative emotion in adversity, (4) previous neuroimaging research has confirmed a remarkable emotion valence effect of ER, even after controlling for arousal ratings [49, 50], and (5) previous neuroimaging research has confirmed that up- and downregulation of emotions engage different sets of neural correlates [33]. Reinterpretation entails changing the meaning of the appraised situation, while distancing requires adjusting the degree of personal involvement (for example, by thinking as an objective observer or an emotionally detached third party) [40]. To enhance comparability, other ER tactics (e.g., thought stopping and distraction) that mobilize different sets of cognition dissimilar to WM (e.g., thought suppression and disengagement of attention) were excluded. All literature regarding affective regulation in the scenarios of reward/loss, social stress, pain perception or fear conditioning was eliminated. The meta-analysis of WM focused on functional imaging studies of n-back designs.

## Materials and methods

**Fig 1:**
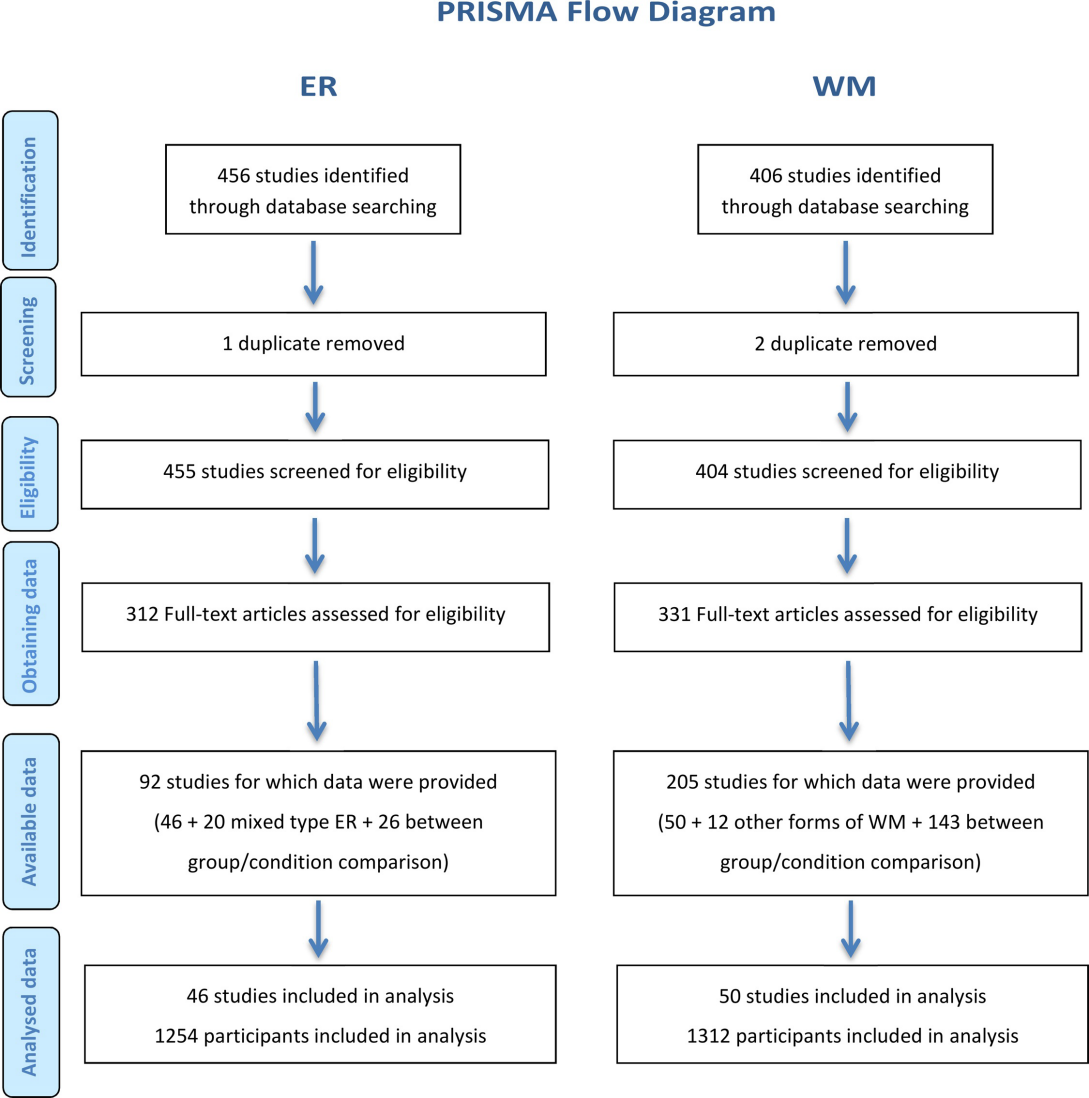
PRISMA flow chart for ER (left) and WM (right).

The conduction of this meta-analysis conformed to Preferred Reporting Items for Systematic Reviews and Meta-Analyses (PRISMA) guidelines [51], summarized and listed below (see **S3 File**). This section contains two parts. The first and second parts, respectively, address ER and WM. PRISMA flow diagram is illustrated in Fig 1.

### Study and contrast selection of ER

#### Eligibility criteria

Peer-reviewed neuroimaging articles with respect to human emotion regulation, published between January 2000 and December 2016.

#### Information source

Electronic dataset of PubMed.

#### Search strategies

1. Species restricted to “Humans”; 2. Search terms were ("emotion regulation" [Title/Abstract] OR ((emotion [Title/Abstract] OR sad [Title/Abstract] OR threat [Title/Abstract] OR fear [Title/Abstract] OR amygdala [Title/Abstract]) AND reappraisal [Title/Abstract])) AND ("functional magnetic resonance imaging" [Title/Abstract] OR "functional MRI" [Title/Abstract] OR fMRI [Title/Abstract]) NOT meta-analysis [Title/Abstract] NOT review [Publication Type].

#### Study records (selection process)

Searched data were included if they satisfied the following criteria: (1) whole brain analysis, instead of seed-based analysis, since pre-selected seeds may bias the results; (2) restriction of ER paradigms to the conventional emotion research that can be accommodated within the process model proposed by Gross [9]; (3) reappraisal tactics of reinterpretation and/or distancing; (4) downregulation of the emotion to aversive or negative valence material; (5) application of a general linear model to localize the neural substrates of ER efforts; (6) reporting of standardized coordinates for activation foci in either Montreal Neurological Institute (MNI) or Talairach space [52]; and (7) healthy non-medicated subjects, mean age > 18 and < 60. Articles only reporting the results of mixed samples (main effect of patients and controls), mixed ER tactics, and mixed emotions (main effect of both positive and negative emotions) were excluded (criterial 8). Patient studies with clear imaging information about healthy controls in the reappraisal of negative emotion were included.

#### Data collection process

This study analyzed 46 neuroimaging studies (1254 participants) of ER out of the 456 articles retrieved from the PubMed search, as listed in Table 1. The incorporated ER contrasts was mainly “decrease emotion minus view/maintain emotion”. It was noted that the experimental materials of the selected ER literature were delivered through a visual channel and were mainly adopted from the International Affective Picture System (IAPS) [53]. Other sources included the NimStim Set of Facial Expressions, Karolinska Directed Emotional Faces and Chinese Affective Picture System [54–56]. The specific reason for each excluded study is listed in Part I of the **S1 File**, and summarized as follows (please refer to the 8 selection criteria described above): (1) region of interest analysis (N = 7); (2) diverse designs that were relevant to ER in a broad sense, such as the regulation in conditions of pain perception, reward/loss, anticipation, conditioning, social interaction, psycho-social stress, interference resolution, mindfulness training, and self-relevance (N = 236). All of these excluded paradigms, despite their significance and importance, are known to engage neural networks different from those reported in conventional emotion research. This study restricted the focus to well-established ER reappraisal tactics, which, remarkably, out-numbered other individually excluded designs (detailed in **S1 File)**; (3) other ER strategies such as thought suppression, expression suppression, listening to music, automatic ER, and emotion labeling/matching (N = 14); (4) up-regulation of emotion (N = 2); (5) resting state connectivity analysis (N = 47), task-related connectivity analysis (N = 9), and correlation/regression analysis (N = 6); (6) unclear coordinate conversion (N = 1); (7) age beyond the range 18–60 (N = 22) and patient study (N = 6); (8) mixed samples, emotions, and tactics (N = 20). Finally, it was noted that some studies used imaging tools other than fMRI (N = 9), and some studies attempted to develop research protocol/stimuli/design/methodology (N = 4). Twenty-six ER studies explored between condition/group comparisons, without reporting the contrast that is of interest in this meta-analysis. One study with sample duplication was noticed. All of these articles were also excluded.

**Table 1:** Studies of emotion regulation included in the meta-analysis.

Author, Year	Experimental Design	Experiment Stimuli	Contrast	N	Age	M/F
Albein-Urios, 2013	Reinterpret (-), Maintain	unpleasant & arousing IAPS pictures	Decrease - Maintain	21	31	20/1
Allard, 2014	Reinterpret+Distance (-) and View	negative film clips	Decrease - View	34	23.8	18/16
Che, 2015	Reinterpret (-), Maintain	aversive CAPS pictures	Decrease - Maintain	29	22.6	14/15
Corbalan, 2015	Reinterpret (-) and View	negative IAPS images	Decrease - View	17	41.4	8/9
de Wit, 2015	Reinterpret (-) and View	fearful stimuli	Decrease - View	38	39.6	18/20
Denny, 2015	Distance (-) and View	negative IAPS photos	Decrease - View	17	24.1	5/12
Domes, 2010	Distance (+,-), Maintain	negative IAPS photos	Decrease - Maintain	33	24.9	16/17
Eippert, 2007	Distance (+,-) and View	threat-related IAPS photos	Decrease - View	24	23.3	0/24
Erk, 2010	Distance (-) and View	negative IAPS photos	Decrease - View	17	43.9	9/8
Goldin, 2008	Distance (-) and View	disgust-inducing clips	Decrease - View	17	22.7	0/17
Golkar, 2012	Reinterpret (-) and View	negative IAPS photos	Decrease - View	58	24	26/32
Hallam, 2015	Diatance (-) and View	disgust and sadness-eliciting IAPS images	Decrease - View	20	20	10/10*
Harenski, 2006	Distance (-) and View	moral-violating IAPS photos and photos from public media	Decrease - View	10	18–29	0/10
Hayes, 2010	Distance (-) and View	negative & arousing IAPS and in-house photos	Decrease - View	25	21.6	14/11
Hermann, 2009	Distance (+,-) and View	aversive IAPS photos	Decrease - View	14	22.1	0/14
Kim, 2007	Reinterpret (+,-) and View	aversive IAPS photos	Decrease - View	10	20.7	0/10
Koenigsberg, 2009	Distance (-) and View	negative IAPS photos (social)	Decrease - View	16	31.8	7/9
Koenigsberg, 2010	Distance (-) and Maintain	negative social IAPS photos	Decrease - Maintain	16	31.8	7/9
Kompus, 2009	Attractiveness rating and View	negative emotional faces from Karolinska Directed Emotional Faces (fear/anger)	Rating - View	18	22.4	11/7
Mak, 2009	At subject's own choice (-)	negative IAPS and media photos	Decrease - View	12	24	0/12
Modinos, 2010	Reinterpret (-) and View	negative IAPS photos	Decrease - View	18	21.1	11/7
Modinos, 2010	Reinterpret (-) and View	negative IAPS pictures	Decrease - View	34	20.4	14/20
Nelson, 2015	Reinterpret (-), Maintain	negative emotional faces from NimStim Set of Facial Expressions	Decrease - Maintain	21	25.2	11/11-1*
Ochsner, 2002	Reinterpret (-) and View	negative IAPS photos	Decrease - View	15	21.9	0/15
Ochsner, 2004	Reinterpret and Distance (+,-) and View	aversive IAPS photos	Decrease - View	24	20.6	0/24
Otto, 2014	Reinterpret (-) and View	fearful faces	Decrease - View	26	24.9	0/26
Paret, 2011	Distance (-), Maintain	anticipatory electrical stimuli (fear/threat)	Decrease - Maintain	21	28	21/0
Paschke, 2016	Distance (-) and View	negative social pictures from Emotional Picture Set	Decrease - View	108	26.1	53/55
Phan, 2005	Reinterpret (-), Maintain	aversive & arousing IAPS photos	Decrease - Maintain	14	27.6	6/8
Price, 2013	Reinterpret (-) and Fixation	sadness and guilt induction by autobiographical memory recall	Decrease - Fixation	11	22.2	3/8
Rabinak, 2014	Reinterpret (-), Maintain	aversive IAPS photos	Decrease - Maintain	21	34.8	21/0
Sarkheil, 2015	Reinterpret (-) and View	aversive IAPS pictures	Decrease - View	14	20–27	6/8
Schardt, 2010	Distance (-) and View	aversive IAPS photos (fear/disgust)	Decrease - View	37	22.6	0/37
Silvers, 2015	Reinterpret (-) and View	negative IAPS pictures	Decrease - View	30	22	17/13
Sripada, 2014	Reinterpret (-), Maintain	aversive IAPS pictures	Decrease - Maintain	49	23.6	26/23
Stephanou, 2016	Reinterpret (-) and View	negative pictures from IAPS, Empathy Picture System database and others	Decrease - View	78	19.91	34/44
van der Meer, 2014	Reinterpret (-) and View	negative IAPS photos	Decrease - View	20	35.5	14/6
van der Velde, 2015	Reinterpret or distance (-) and View	negative IAPS photos	Decrease - View	51	37.1	28/23
van der Velde, 2015	Reinterpret or distance (-) and View	negative IAPS photos	Decrease - View	16	22.1	8/8
Vanderhasselt, 2013	Reinterpret (-) and View	negative IAPS photos	Decrease - View	42	21.3	0/42
Veit, 2012	Distance (+,-) and View	aversive IAPS photos	Decrease - View	11	21–28	3/8
Walter, 2009	Distance (-) and View	negative IAPS photos	Decrease - View	20	24	0/20
Winecoff, 2011	Distance (-) and View	negative IAPS photos	Decrease - View	42	23 and 69	22/20**
Winecoff, 2013	Distance (-) and View	negative IAPS photos	Decrease - View	31	25	10/21
Zhang, 2012	Reinterpret (-) and View	negative IAPS photos	Decrease - View	27	20.6	4/23
Ziv, 2013	Reinterpret (-) and View	anger and contempt faces	Decrease - View	27	32.6	14/13

+ and - in parentheses indicates up- and down-regulation of induced emotion. CAPS: Chinese Affective Picture System; IAPS: International Affective Picture System.

Contrasts are all about negative/aversive stimuli, e.g., Decrease negative emotion–View/Maintain negative emotion.

* gender information not clear.

** young/old, gender information not clear.

#### Data items

The peak coordinates of the neuroimaging reports were extracted for quantitative analysis (see next section). Only positive activations in the contrast “reappraisal minus baseline” were analyzed since activations indicate ER efforts, while deactivations may represent attenuated emotional responses that were not of interest in this study.

### Study and contrast selection of WM

#### Eligibility criteria

Peer-reviewed neuroimaging articles of 2-back WM tasks, published between January 2000 and December 2015.

#### Information source

Electronic dataset of PubMed.

#### Search strategies

1. Species restricted to “Humans”; 2. Search terms were ("working memory" [Title/Abstract] OR "short-term memory" [Title/Abstract]) AND (n-back [Title/Abstract] OR 2-back [Title/Abstract]) AND ("functional magnetic resonance imaging" [Title/Abstract] OR "functional MRI" [Title/Abstract] OR fMRI [Title/Abstract]) NOT meta-analysis [Title/Abstract] NOT review [Publication Type].

#### Study records (selection process)

Searched data were included if they satisfied the following criteria: (1) whole brain analysis, instead of seed-based analysis, since pre-selected seeds may bias the results; (2) restriction of WM paradigms to the 2-back design; (3) restriction of experimental material to visual stimuli because most previous ER research uses visual stimuli and the neural networks of WM seems to be different between visual, auditory and tactile modalities [57, 58]; (4) application of a general linear model to localize the neural substrates of ER efforts; (5) reporting of standardized coordinates for activation foci in either Montreal Neurological Institute (MNI) or Talairach space [52]; and (6) healthy non-medicated subjects, mean age > 18 and < 60. Articles only reporting the results of mixed samples (main effect of patients and controls) were excluded (criteria 7). Patient studies with clear neuroimaging information of healthy controls in performing 2-back tasks were included.

#### Data collection process

This study analyzed 50 n-back neuroimaging studies (1312 participants) out of the retrieved 406 articles, listed in Table 2. The inclusion criteria were similar to those of ER, and the main contrast of interest was “2-back minus 0-back”. The specific reason for each excluded study is listed in Part II of the **S1 File**, and summarized as follows (please refer to the 7 selection criteria described above): (1) region of interest analysis and incomplete report of coordinates (N = 48); (2) other forms of WM paradigms, such as emotional and social variants of WM tasks, face-matching WM task, modified Stroop task, combined WM and dichotic-listening paradigm (N = 12); (3) stimuli not delivered through a visual channel, such as tactile and auditory WM tasks (N = 11); (4) resting state analysis (N = 1), multi-variate analysis (N = 3), task-related connectivity analysis (including path analysis; N = 12), correlation/regression analysis (N = 12), independent component analysis (N = 9), graph theoretical approach (N = 4), machine learning/classification (N = 2), trend analysis (N = 1), permutation analysis for parametric effect (N = 1); (5) unclear about coordinate conversion (N = 14); (6) age beyond the range 18–60 (N = 40), patient study (N = 9), case study (N = 6), and participants not in normal states (i.e., in nicotine withdrawal or sleep deprivation, N = 2); and (7) mixed samples (N = 11). In addition, it was noted that some studies used imaging tools other than fMRI (N = 9), and some studies aimed to develop a methodology (N = 2). Since n-back is a mature design that has been used to explore a broad range of neuro-psychiatric conditions, the WM studies in recent years tend to ignore the contrast of "2-back minus baseline" and only report the results of "between condition" or "between group" contrasts (N = 143). Two studies with sample duplication and two studies focused on behavior/performance were noticed. All of these articles were excluded.

#### Data items

The peak coordinates of the neuroimaging reports were extracted for quantitative analysis (see next section). Only positive activations in the contrasts that included “2-back minus baseline” were analyzed since the deactivated default-mode network was not the focus of this study.

**Table 2:** Studies of working memory included in the meta-analysis.

Author, Year	Experiment Stimuli	Contrast	N	Age	M/F
Allen, 2006	letter	2-back - control	10	23–35	8/2
Barch, 2007	word/face	2-back - encoding	120	27.2	50/70
Binder, 2006	letter	2-back - control	12	23.5	7/5
Bleich-Cohen, 2014	number	2-back - control	20	26.4	12/8
Blokland, 2011	number	2-back - control	319	23.6	120/199
Cader, 2006	letter	(1-, 2-back) - control	16	39	6/10
Chang, 2010	letter	2-back - fixation	21	49.7	21/0
Chechko, 2015	letter	2-back (conjunction of placebo and glucagon conditions) - control	40	24.5	20/20
Deckersbach, 2008	letter	2-back - fixation	17	25.6	0/17
Dima, 2014	letter	2-back - control	40	31.5	20/20
Drapier, 2008	letter	2-back - control	20	41.9	10/10
Drobyshevsky, 2006	letter	2-back - control	31	40.9	16/15
Garrett, 2011	letter	2-back - control	19	34.9	13/6
Habel, 2007	letter	2-back - control	21	30.8	21/0
Haller, 2005	letter	3-back - control	16	25.2	8/8
Harding, 2016	number	2-back - control	25	25.5	14/11
Harvey, 2005	letter	(1-, 2-, 3-back) - control	10	29	5/5
Honey, 2000	letter	2-back - control	20	39.3	20/0
Honey, 2003	letter	2-back - control	27	35.1	21/6
Jansma, 2004	spatial position	(1-, 2-, 3-back) - control	10	27.8	8/2
Johannsen, 2013	letter	2-back - control	12	26.1	4/8
Joseph, 2012	letter	2-back - control	19	25	0/19
Koppelstaetter, 2008	letter	2-back - control	15	25–47	15/0
Koshino, 2008	faces	(0-, 1-, 2-back) - fixation	11	28.7	10/1
Lycke, 2008	syllable	2-back - viewing blue screen	26	23.4	12/14
Lythe, 2012	letter	(1-, 2-, 3-back) - control	20	26.7	20/0
Malisza, 2005	spatial position	1-back - control	10	18–33	*
Monks, 2004	letter	2-back - control	12	45.6	12/0
Oflaz, 2014	letter	2-back - control	9	44.6	7/2
Park, 2011	letter	2-back - control	10	23.7	10/0
Paskavitz, 2010	letter	2-back - control	17	35.1	8/9
Quide, 2013	number	2-back - control	28	33	14/14
Ragland, 2002	letter	2-back - control	11	32.2	6/5
Ravizza, 2004	letter	3-back - control	21	18–37	10/11
Reuter, 2008	number	2-back - control	49	27.4	19/30
Ricciardi, 2006	shape	1-back - rest	6	28	6/0
Rudner, 2013	picture (semantics)	2-back - control	31	28.4	8/23
Sanchez-Carrion, 2008	number	2-back - control	14	24.2	11/7-4
Scheuerecker, 2008	letter	2-back - control	23	32.6	19/4
Schneiders, 2011	abstract patterns	2-back - control	48	23.7	22/26
Seo, 2012	letter	2-back - control	22	38.3	0/22
Seo, 2014	letter	2-back - control	34	59.3	0/34
Stoodley, 2012	letter	2-back - control	9	25.5	9/0
Stretton, 2012	spatial position	2-back - control	15	27	4/11
Sweet, 2010	letter	2-back - control	12	38.7	5/7
Thomas, 2005	letter	2-back - control	16	37.6	11/5
Townsend, 2010	letter	2-back - control	14	30.8	6/8
van der Wee, 2003	spatial position	(1-, 2-, 3-back) - control	11	34.8	0/11
Yan, 2011	spatial position	2-back - control	28	20.9	12/16
Yoo, 2004	letter	1-back - rest	14	26.3	9/5

* gender information not clear.

In the contrast, control means 0-back condition.

Since the fMRI research of ER began to burgeon several years after Gross's seminal work (psychological approach) in 1998 [9], we set the start date of Pubmed search to 2000. During the searching process, we noticed that the WM studies in recent years tend to ignore the contrast of "2-back minus baseline" and only report the results of "between condition" or "between group" contrasts. This trend is understandable because n-back is a mature design that has been used to explore a broad range of neuro-psychiatric conditions. Detailed information about conventional contrasts will not provide useful information to the readers but only cost journal space. Of the 39 articles in 2015, only 1 article fits our need. We thus stopped the WM literature search in 2015.

### ALE procedure

A meta-analysis that accommodated idiosyncratic study-level variations was performed by the algorithm ALE, implemented in the software GingerALE (http://www.brainmap.org/ale) [46, 47]. ALE is a coordinate-based method that reveals consistent locations of neural activation/deactivation in the brain across different neuroimaging studies. The calculation starts by generating 3D-modeled activation maps for each included contrast in which the peak coordinates are “blurred" with a Gaussian function, with the "full width at half maximum" empirically determined by subject size. An ALE image is constructed by the union of all the modeled activation maps to produce voxel-wise ALE scores, which provide the basis for ALE statistics, with the accumulated scores constituting a histogram and probability table. To determine significance, the ALE map is tested against a null distribution map derived from permutation procedures. The peak activation coordinates of each contrast were all aligned or transformed to Talairach space, and the ALE results for ER and WM were thresholded with a cluster level inference less than 0.05 [59].

GingerALE provides a module for contrast analysis, which was realized by another permutation process. In brief, the included contrasts for ER and WM were pooled together and then randomly assigned to two groups to produce a new contrast, which was then repeated 10,000 times. Significant differences between ER and WM were determined by comparisons with the permutated results, with a false discovery rate (FDR) of less than 0.05. The neuroimaging results were illustrated in a sliced standard brain using the Analysis of Functional NeuroImages software package (AFNI) [60].

## Results

**Fig 2:**
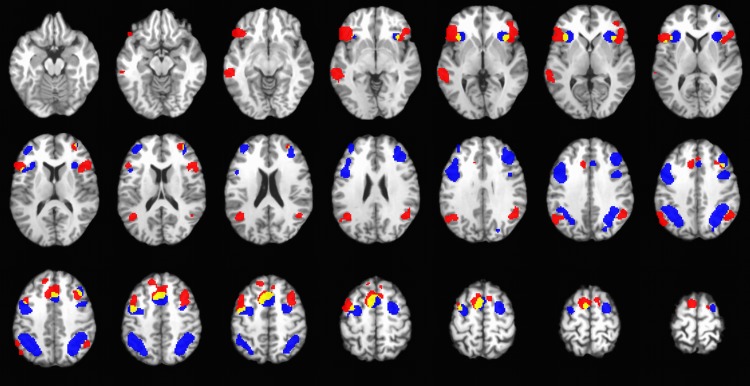
Mosaic view of emotion regulation (red) and working memory (blue) networks. Brain regions recruited by both tasks are depicted in yellow. Maps were thresholded at P < 0.05 and were superimposed on a T1-weighted structural image in axial sections from z = -8 mm to z = 61 mm, with a between-slice gap of 3 mm.

The ALE-meta-analytical results of ER and WM were grossly concordant with previous reports, see Tables 3 and 4. The ER network comprised the ACC/dmPFC, left middle temporal gyrus, bilateral SFC, MFC and IFC, and the temporoparietal junction (TPJ). The core network of WM was also replicated in the meta-analysis and included bilateral MFC, bilateral inferior parietal lobules, pre-SMA and ACC, and bilateral dorsal anterior insula. It was observed that the frontal and TPJ clusters of ER were different from those of WM. In Fig 2, ER and WM activations are, respectively, colored red and blue, while the overlapped voxels are yellow. The gender ratios of the included ER and WM studies are 0.67 and 0.94, respectively.

**Table 3:** Left: ALE clusters resulting from "downregulation > control" contrasts of 46 emotion regulation studies, with cluster level inference *P* < 0.05.Right: The contrast of ER > WM, with *P* < 0.05.

Structure	Volume (mm3)	x	y	z	Volume (mm3)	x	y	z
Superior frontal gyrus (L, BA 6)	11544	-6	6	60	1416	-10.3	12	62
(L, BA 8)		-14	38	44	1088	-15.5	41	40.5
(R, BA 6)		12	12	58	128	12	16	58
Anterior Cingulate Gyurs (L, BA 32)		-8	18	42	512	-12	18	36
Medial frontal cortex (R, BA 8)		4	30	40	432	4	28	46
Middle frontal gyrus (L, BA 6/8)	3544	-40	-2	48	760	-44	9	50
Middle frontal gyrus (R, BA 8)	3104	36	16	44	1384	33.2	16.2	46
Middle frontal gyrus (R, BA 10)	800	30	48	16				
Inferior frontal gyrus (L, BA 45/47)	7848	-44	24	-2	4552	-45.6	27.3	-1.4
Inferior frontal gyrus (R, BA 45)	6600	46	28	0	3024	50.4	27.7	4.9
Temporoparietal junction (L, BA 40)	4416	-54	-54	38	3648	-43.3	-60	25.3
(L, BA 39)		-40	-56	20				
Temporoparietal junction (R, BA 40)	3400	52	-54	36	1584	48	-58	30
(R, BA 39)		46	-56	24				
Middle tempoarl Gyrus (L, BA 21)	3848	-58	-36	-4	3136	-59.1	-34.6	-2.5

The peak coordinates are reported in Talairach space. BA represents Brodmann area. L: Left; R: Right.

**Table 4:** Left: ALE clusters resulting from "2-back (mainly) > baseline" contrasts of 50 working memory studies, with cluster level inference *P* < 0.05. Right: The contrast of WM > ER, with *P* < 0.05.

Structure	Volume (mm3)	x	y	z	Volume (mm3)	x	y	z
Middle frontal gyrus (L, BA 6/9)	15928	-44	2	34	6424	-39.3	-1.1	36.8
		-26	-6	52		-27.6	-9.8	50.4
Insula (L, BA 13)		-32	20	4	1480	-25.8	17.7	6.3
Middle frontal gyrus (L, BA 10)	2296	-36	44	18	224	-36	52	22
Middle frontal gyrus (R, BA 9/10)	6408	40	30	30	3608	40.6	30.1	26.6
		34	44	24				
Middle frontal gyrus (R, BA 6/9)	6032	26	-2	58	2408	25.3	-1.3	57.8
		42	6	34	1448	42.5	7	30.5
Insula (R, BA 13)	3336	30	20	4	2248	28.3	21.3	6.3
Pre-SMA (BA 6)	6928	2	10	48	2304	3.4	7.3	47.1
Inferior parietal lobule (L, BA 40)	9480	-34	-56	40	8968	-34.7	-54.4	40.9
(L, BA 7)		-26	-64	38				
Inferior parietal lobule (R, BA 39)	10808	30	-58	38	9496	33.6	-53.4	40.2
(R, BA 40)		40	-46	40				
Cerebellum (L)	2832	-28	-62	-26	2729	-30.5	-58.5	-27.7
Cerebellum (R)	1256	30	-58	-28	40	32	-54	-34

The peak coordinates are reported in Talairach space. BA represents Brodmann area. SMA: supplementary motor area; L: Left; R: Right.

### ALE analysis of ER and WM

The peak ALE coordinates of ER in the IFC (left -44, 24, -2; right 46, 28, 0) were lateral to those of WM in the anterior insula (left -32, 20, 4; right 32, 20, 4), with an average distance of 15.3 mm. The spatial discrepancy implied a dissociation of ER and WM in the inferior forebrain. The WM clusters in the MFC extended from the precentral cortex to the rostral frontal region (Brodmann areas 6 to 10) and occupied approximately 18224 mm^3^ and 12440 mm^3^ in the left and right hemispheres, respectively. Not covered in the WM ALE map, ER clusters in the MFC were smaller (3544 mm^3^ in the left and 3105 mm^3^ in the right hemispheres) and were situated dorsally to the WM analogs, near the border between the MFC and SFC. The distances between the peak coordinates of ER and WM in the MFC were 15.1 mm (left) and 21.6 mm (right). In addition, ER activated the bilateral SFC (much larger on the left side) that were absent in the WM ALE map. The above findings in the PFC (superior, middle and inferior) support the distinction of hot and cold cognition from the perspective of neural correlates. ER and WM both engaged the dorsal part of the midline PFC, and their spatial extent overlapped considerably. It was noted that the peak ER foci was situated in the ACC and was anterior to the peak WM foci in the pre-SMA. The dissociation in neural correlates of ER and WM was also observed in the posterior brain region, with ER in the TPJ (lower Z coordinate) and WM in the inferior parietal lobule (higher Z coordinate). In summary, there is an interesting pattern where the activation foci of ER and WM were on one hand dissociable and on the other hand in close proximity. The detailed results are depicted in Tables 3 and 4.

### Contrast analysis of "ER vs. WM"

**Fig 3:**
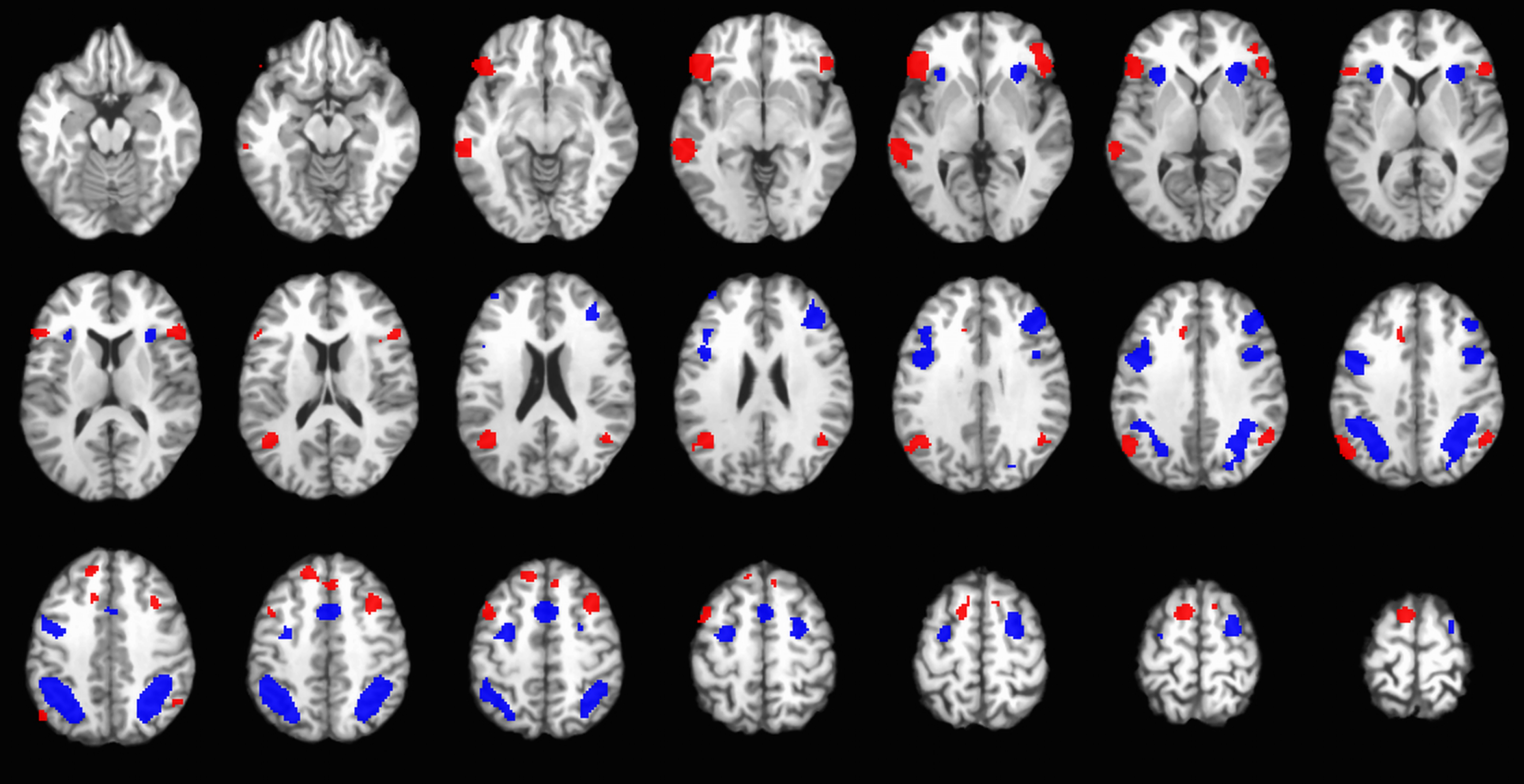
Mosaic view of the contrast "emotion regulation > working memory" (red) and "working memory > emotion regulation" (blue). Maps were thresholded at P < 0.05 and were superimposed on a T1-weighted structural image in axial sections from z = -8 mm to z = 61 mm, with a between-slice gap of 3 mm.

The contrast analyses of "ER > WM" and "WM > ER" nearly replicated all of the significant meta-analytical results of the ER and WM maps, supporting the above observation that the activation maps of ER and WM were grossly non-overlapping; see Tables 3 and 4. Fig 3 illustrates the contrast analyses of "ER > WM" and "WM > ER" that are, respectively, colored red and blue, with the overlapped voxels colored yellow (as in Fig 2). Since the neural correlates of ER and WM appears to be largely dissociable (except the dmPFC/cingulate area), Figs 1 and 2 look similar. An additional conjunction analysis was also performed, which was used to show the similarity between the ER and WM datasets. The results of ER–WM conjunction are highly concordant with the intersection of ER and WM activation maps that have already been illustrated in Fig 2 (in yellow color) and are thus summarized in the **S1 File** (Part III). All the significant conjunction clusters aggregated in the PFC (i.e., the overlapped voxels in the posterior brain region did not survive the conjunction analysis). The total volumes of the significant PFC foci in ER, WM and conjunction analysis are 33440 mm^3, 40928 mm^3 and 6640 mm^3, respectively. After excluding the highly overlapping conjunction foci in the dmPFC/cingulate area (3672 mm^3; please refer to the **S1 File** for details), the ratio of conjunction to non-conjunction voxels/volumes in the lateral PFC of ER and WM are, respectively, 0.10 (2968/29768) and 0.08 (2968/37256), providing additional persuasive evidence that the neural substrates of ER and WM are dissociable in the lateral PFC (and posterior brain region).

## Discussion

**Fig 4:**
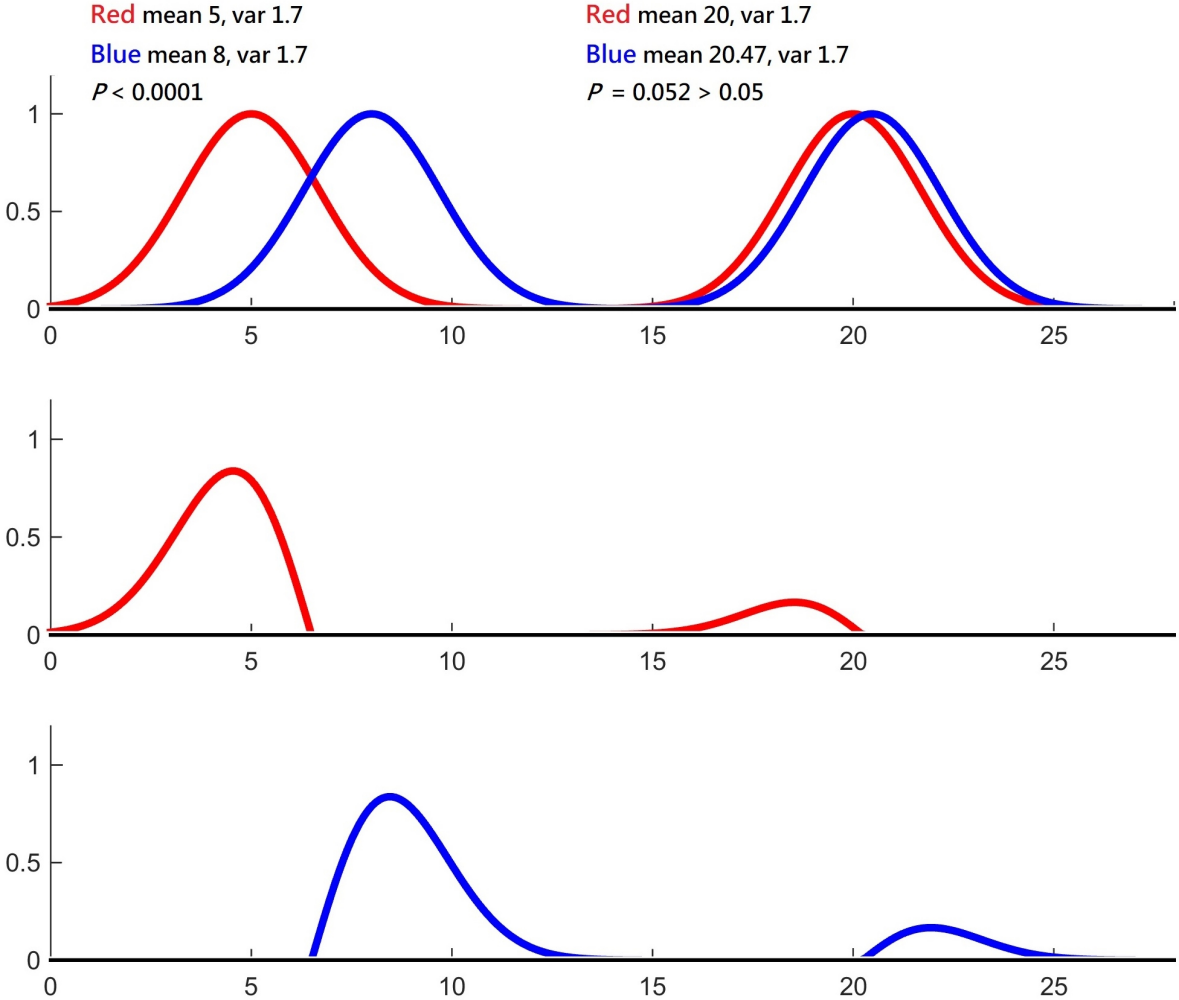
A heuristic about separated clusters is demonstrated. Left: when two clusters (Red and Blue) are separable (upper row; assume the X-axis is the coordinate in the brain, P < 0.0001), the contrasts "Red > Blue" (middle row) and "Blue > Red" (lower row) largely retain the respective distribution of Red and Blue clusters. Right: when two clusters are not separable (P = 0.052), the contrasts "Red > Blue" (middle row) and "Blue > Red" (lower row) will lose their original shape and become remarkably dampened (and hence insignificant). In the latter case, when the maximum value (Y-axis) of one distribution is much larger than the other one, one subtraction contrast may retain its significance, while the other will lose its significance (not shown). In this simulation, the data number is 100 for each cluster.

Convergent evidence from rat and human studies has highlighted the importance of the PFC in ER [61]. In terms of evolution, Homo sapiens are characterized by a dramatic expansion of the forebrain with specialized neural circuits to perform different cognitive functions, such as linguistic processing, cognitive control, and prospective memory [62–64]. Whether the human PFC has evolved to possess specialized modules that underlie "cold" (e.g., WM) and "hot" (e.g., ER) cognition is an issue of both great interest and theoretical importance. It has been suggested that both ER and WM require the maintenance and manipulation of goal-related information (psychological), and present similar neural activation pattern (neurobiological) [1, 17, 26–28]. This study aimed to investigate the hypothesis that the downregulation of negative emotion via reappraisal relies on the same neural network as in a WM task. The meta-analytical tool GingerALE was applied with a particular emphasis on the PFC. The ALE results of ER and WM are highly concordant with previous meta-analytical reports [33–36], and their associated neural correlates differed in all the three sectors of lateral PFC (IFC, MFC and SFC) and in the posterior brain region. In contrast to the canonical network of WM (insula, fronto-parietal network, pre-SMA/ACC; named the task activation ensemble by another research group [65] and regarded as the fundamental structure to support top-down executive control [66]), ER activated bilateral IFC, MFC, SFC, dmPFC and TPJ. The overlapping lateral PFC volumes of both ER and WM were less than 10 percent, and the statistical comparisons of the contrasts "ER > WM" and "WM > ER" largely retained the peak coordinates of the ER and WM activation maps (see Tables 3 and 4), respectively. The results altogether indicate that the major activation foci of ER and WM were separated far enough (the heuristic is explicated in Fig 4), and the hypothesis that ER is endorsed by a specialized neural circuit is supported. Although ER and WM ALE maps overlapped considerably in the dmPFC and cingulate, the respective peak coordinates of ER and WM were in the dmPFC and pre-SMA, and both survived statistical comparison.

With respect to the inferior forebrain, previous WM reports mentioned both anterior insula (more) and IFC (less) [67–69], whereas downregulation of negative emotion consistently involved greater activity in the IFC (which may extend to lateral orbitofrontal cortex; OFC) [22, 33, 34, 50]. The functions of the IFC and insula are very different. The caudal portion of the left IFC used to be regarded as a linguistic center, commonly known as Broca's area. Recent neuroimaging research has extended the understanding of the functions of the IFC, which may buttress a wide range of behavioral controls, such as response inhibition, risk aversion, hierarchical action plans, imitation (mirror neuron system), emotion–cognition interaction and ER [38, 62, 70–72]. The insular cortex has been associated with a multitude of functions, such as interoceptive awareness, emotion processing, visceral and autonomic functions, and motor control. There are functional dissociations in different sub-regions of the insula, namely, the dorsal anterior, ventral anterior, central and posterior divisions, which have been identified by cytoarchitectonic and functional parcellation studies [73, 74]. The dorsal anterior insula is regarded as playing an important role in general cognition, and the network positively correlated with it corresponds nicely with the canonical WM network, as demonstrated in this and previous studies [74]. Given that the performance of WM is predictive of academic achievement and intelligence [75], the orchestrated neural substrates of WM could underlie various cognitive capabilities. As to why some WM research reported IFC activity, there are two possibilities worth considering. First, since the IFC hosts the linguistic center, it might facilitate verbal WM in certain conditions [76]. Concordant with the conjecture, a lesion study suggested that the IFC may be associated with articulatory rehearsal in a WM task [77]. Second, neuroimaging signals of neighboring voxels are usually smoothed by kernels of a certain width to enhance the signal-to-noise ratio. The activities of the dorsal anterior insula may thus spill over to the adjacent IFC due to the smoothing procedure. Nevertheless, the meta-analyses of the 2-back tasks have confirmed the consistency and robustness of the dorsal anterior insula activity in WM [35, 36]. Moreover, inverse relationships between the neural activities in the ventrolateral PFC (vlPFC) and amygdala and between the neural response in the vlPFC and negative emotion experiences have been consistently replicated by previous neuroimaging reports [40, 78–80]. In the reappraisal of social emotion, the activity in IFC was positively correlated with the frequency and capacity of ER in daily life (as indicated by the Emotion Regulation Questionnaire [81]) [39]. The IFC may thus serve as an avenue for the PFC to modulate saliency, arousal, and perceptual processing to decrease felt emotions.

Although previous research frequently referred the dorsolateral PFC finding of ER to WM processing [17, 26, 27], ALE meta-analysis showed that the foci of ER and WM in the MFC were different. Since the vlPFC is more relevant to the limbic system [82], if there is a functional specialization of ER in the MFC, it is expected to occur in the ventral portion. However, the ER foci in the MFC were observed to be dorsal to the WM foci. Another unexpected finding was the (left) SFC, which is located at the vertex of the cerebrum, in the proximity of the dmPFC. Careful surveying revealed that this spot was neither in the dorsal attention system nor compatible with the frontal/supplementary eye field [44, 83, 84]. The apical SFC is reportedly associated with introspection, which is suppressed while processing demanding perception tasks [85]. The position of the ER foci in the dorsal MFC is convenient for accessing the SFC, possibly via short-range connections. A recent study revealed that a similar region in the SFC showed hyper-activation in problem stimulant users during anticipating and experiencing pleasant soft touch, whereas the neural activity was absent or decreased in former drug users and controls [86]. The abnormal (disinhibited) apical SFC may transform external cues to internal motives in substance abusers. In contrast, social anxiety disorder was reported to show a marked decrease in neural activity in the left SFC during ER (peak coordinates [–3, 4, 63], very close to our meta-analytical finding of [–6, 6, 60]) [15]. The neuroimaging reports from the exploration of psychopathology support the idea that the apical SFC is an important neural structure for ER that has long been ignored in the previous literature. As to the midline forebrain structure, the neural correlates of ER (dmPFC) and WM (pre-SMA) in the dorsal midline PFC were proximal; the former is related to self-referential function [42], while the latter is related to voluntary control over actions in conflict response situations [87]. The findings on the MFC, SFC and dmPFC all together can be integrated under a framework of self-related cognitive and introspective synergism.

Mega et al. distinguished two limbic sub-systems (i.e., hippocampus/cingulate-centered and OFC/amygdala-centered divisions) [88]. The ER foci MFC-SFC-dmPFC and IFC may modulate the two limbic sub-systems to achieve ER, which in turn may lay the neural foundation for cognitive therapy and psychotherapy. This proposition is supported by the observation that the strengths of the amygdala coupling with the OFC and with the dmPFC predict the extent of attenuation of negative affect following reappraisal [89]. The ALE results may provide further insight to the debated issue of emotion–cognition dichotomy, which has been raised by rigorous neuroscientific reviews [43, 90]. An alternative account, as provided by this study, could be that the PFC possesses specialized regions for human emotions. This perspective reconciles the facts that emotion and cognition interact intimately (against the dichotomy) and that emotion and cognition are treated as distinct psychological entities (for the dichotomy). The neural circuit in charge of reappraisal may also produce emotions through top-down control [91]. In other words, the holistic picture of an "emotion circuit" should not be restricted to conventional affective centers (e.g., the amygdala, anterior insula, and limbic cortex) but should also incorporate emotion-related PFC substrates as indispensable members, which are at least partly separable from the PFC centers of cold cognition.

The posterior brain findings (i.e., the TPJ and left middle temporal gyrus) are much less appreciated in ER literature. However, the functions of the TPJ are closely related to ER, such as social interaction, theory of mind, language comprehension, empathy, and context updating (note: the language function of TPJ seems to be left-lateralized, while other TPJ functions tend to be right-lateralized) [92–94]. It has been shown that the lateral PFC can be divided into dorsal and ventral divisions, and the former and the latter possess structural connections with the parietal and the temporal/limbic regions, respectively [82]. Concordantly, a resting fMRI study revealed significant functional connectivity between the TPJ and ventral PFC [95]. The dissociation of ER and WM in posterior brain regions may reflect the topological organization of the white matter bundles that bridge the IFC and TPJ and the MFC and inferior parietal lobule. The left middle temporal gyrus has been suggested to be a heteromodal cortex that responds mainly to speech but also to visually presented words, faces and objects [96]. Comparisons of ER tactics revealed that this region is robustly activated in the reinterpretation condition but not in other tactics, such as distraction or expressive suppression [97]. Accordingly, the left middle temporal gyrus may be a tactic-specific brain region in ER, may address the linguistic processing/updating of meaning and not be engaged in WM. Our findings in the TPJ and left middle temporal gyrus agree with a previous report that emphasized the importance of the semantic process in the reappraisal of negative emotion [98].

Our meta-analytical results disclose that the ER network is distinct from but adjacent to the WM counterpart. The neighboring regions in the PFC may share similar computational characteristics. Storing information temporarily and manipulating/updating the information online are the essential features of WM [29, 30], which appears to be the foundation of various cognitive capabilities [75]. Previous ER research persuasively infers the similarities between WM and ER; the latter also entails the mental processes of maintenance (of the ER tactic and the confronted emotional material in the mind) and manipulation (of the denotation of the emotional material) [1, 31, 32]. Concordant with the above speculation, people with higher WM capacity are more successful in ER, such as in the suppression and reappraisal of emotions [28]. From a neuroscientific perspective, the "adjacency" of ER–WM neural substrates nicely accommodates the two seemingly contradictory observations: the performances of WM and ER are correlated [28], and the trainings of affective WM and cognitive WM are not transferable (already described in the **Introduction**) [45]. In addition to reconciling the debate of emotion–cognition dichotomy, this study may provide novel insight about the distinction of hot and cold cognitions [99]. Hot cognition generally refers to information processing with an emotional influence, contrary to cold cognition that is emotionally/motivationally neutral and generally lacks direct personal relevance. From the neuroscientific perspective, as revealed in this study, hot cognition can be conceptualized as the mental operations that involve the PFC modules with the ability (or privilege) to access the limbic system (and vice versa). Two PFC-to-limbic routes are elucidated, i.e., the ER foci of IFC and MFC-SFC-dmPFC that may, respectively, modulate the OFC/amygdala and hippocampus/cingulate limbic divisions. It is noteworthy that the dorsolateral PFC and dmPFC are the major targets of transcranial magnetic stimulation to treat depressive disorders [100]. The results of this report suggest that the magnetic stimulation may actually spread to nearby ER foci in the MFC and SFC to exert therapeutic effects. It would be interesting to examine whether the refinement of the stimulation sites based on ER substrates in the PFC would improve the treatment efficacy.

Although ER has been studied from various perspectives, there is some ambiguity worthy of clarification. First, the jargon in ER literature is sometimes confusing. For example, the term "emotion suppression" was occasionally adopted to indicate the cognitive operation of distancing, which is very different from the original denotation of expressive suppression by Gross [9, 101]. In addition, some studies used the term "voluntary suppression" but in fact requested that the participants used a reinterpretation tactic to regulate their emotions [23]. According to Gross and John, expressive suppression is a response-focused ER (conceptually a type of response inhibition) that could be achieved by instructing the participants to hide their emotions so that the observers could not detect what they are feeling [12]. Distancing and reinterpretation, on the other hand, represent two of the many tactics of antecedent-focused ER and actually belong to a reappraisal strategy. The confusion in the nomenclature may cause the risk of the ER meta-analyses to combine inhomogeneous studies under the category of suppression. It is evident that these ER tactics involve distinct neural and psychological mechanisms since reappraisal would decrease the felt emotion but expressive suppression might increase subjective emotion intensity. Second, ER tactics are quite diverse and may engage different sets of neural substrates. In contrast to other ER tactics (e.g., distraction, thought interruption, imagination, and counteracting negative emotion with positive memory), reinterpretation and distancing are both adaptive in the social context, commonly utilized by research, and importantly, comparable to WM from both psychological and neural perspectives [1, 17, 26–28]. Comparability is a critical factor for disentangling the paramount issue of whether there are emotion-specialized circuits in the PFC with reference to pertinent (or pre-assumed) cognitive functions. Based on the results of this report (i.e., WM vs. reinterpretation/distancing), it would be valuable to investigate the neural correlates of expressive suppression vs. response inhibition (e.g., Go/No-Go), and of attention deployment in emotional vs. cognitive contexts. Moreover, the conclusions provided by meta-analysis may be hard to achieve through individual experiments since, for example, the findings in the MFC and SFC tended to be attributed hastily to standard WM and attention, a reminder of the criticism of reverse inferences in neuroimaging research (a reverse inference is to infer a cognitive process from the activation of a particular brain region) [102, 103]. It is thus beneficial to re-scrutinize the emotion–cognition debates using the meta-analytical method in other cognitive domains. According to a recent power analysis [59], it is recommended to have at least 20 neuroimaging studies in a meta-analysis. Although we only looked up Pubmed database, the retrieved articles obviously outnumber this lower limit. For the meta-analytic research of other ER strategies, the literature number is expected to be much lower than that of reinterpretation and distancing. It is worthwhile to consider broader databases in literature search, such as PsycINFO and EMBASE.

### Limitations

Gender differences are a potential confounding variable of this research. Regarding the participants in the neuroimaging literature, females outnumber males in ER studies, while the opposite trend is observed in WM reports (not in this meta-analysis). The gender imbalance originates from the fact that males and females activate more robust and distributed networks in WM and in ER tasks, respectively. Under a null hypothesis that ER and WM utilize the same neural resources, a meta-analytical comparison of the two functions is expected to yield a higher possibility of overlapping maps and hence a more conservative result. We thus infer that our positive results are not caused by gender bias. A recent meta-analysis of WM revealed consistent networks across both sexes [104], while gender differences could be more significant in ER [105]. Nonetheless, the gender issue of ER remains an open question to be explored in future research. It is noteworthy that previous ER studies have suggested the involvement of the orbital and ventral medial PFC in ER, which was not seen in our meta-analysis [21, 61]. Since the ventral forebrain is subject to MRI susceptibility artifacts, the possibility of a false-negative finding due to signal dropout should be considered. Neuroimaging tools, especially fMRI, have been acknowledged to be powerful in localizing neural substrates associated with mental processes. However, whether two nearby clusters should be viewed as the same or different entities is a difficult and unresolved issue. The heuristic adopted by this study, i.e., comparing the patterns of significant clusters with those after contrast calculations, could be over-conservative and not a generalizable standard for other research. It is of great theoretical interest to examine whether the PFC circuit of a particular ER tactic remains the same across different contexts, such as in negative emotion, financial loss, social stress, or fear conditioning [106–108]. Comparisons of ER tactics with other validated WM variants, such as complex span tasks, may provide further understanding of the specificity and commonality between ER-related and (cold) cognition-related neural circuits [109].

## Conclusions

The cognitive capability to attenuate negative emotional responses is important for adaptive social functioning and crucial for psychotherapy. This meta-analytical study examined whether ER was processed by cold cognition. Given that cognitive reframing to modulate emotional content is conceptually similar to cognitive maintaining/updating/manipulating online information, to ensure comparability, reappraisal ER tasks (reinterpretation and distancing) were contrasted with 2-back WM tasks. Both commonality (minor) and uniqueness (major) in the neural correlates of ER and WM were elucidated. Taking the canonical WM network as a reference, the dmPFC/ACC was partially shared by both mental operations, while differential neural circuits were delineated in both forebrain (IFC, MFC and SFC) and posterior brain (TPJ and left middle temporal gyrus) regions. The findings in the SFC-dmPFC are believed to be related to the introspective process of ER. The ER foci MFC-SFC and IFC may, respectively, modulate the hippocampus/cingulate-centered division and the OFC/amygdala-centered division of the limbic system. The demonstrated emotion-related circuit in the PFC and TPJ could be integrated with conventional emotion centers (subcortical and limbic structures) to provide a fuller picture of the emotion network. Our discovery of adjacent and distinct ER foci in the PFC may reconcile the debated emotion–cognition dichotomy, and characterize the distinction between hot and cold cognitions. Future studies may consider applying meta-analytical methods to decipher ER-specific circuitry in other psychological domains (such as response inhibition and attention deployment) and in other contexts (such as the regulation of social stress, pain perception, fear conditioning and financial loss).

## Supporting information

S1 File S1_File. The supplementary information contains three parts. The first two parts summarize the material excluded from the meta-analyses of ER and WM. The third part describes the results of the ER-WM conjunction analysis.
(DOCX)

S2 File S2_File. The minimal data set necessary to replicate these study findings are provided as a supplementary file.
(ZIP)

S3 File PRISMA_checklist_TWLee. (DOCX)

## References

[1] McRae K, Gross JJ, Weber J, Robertson ER, Sokol-Hessner P, Ray RD, et al The development of emotion regulation: An fmri study of cognitive reappraisal in children, adolescents and young adults. Soc Cogn Affect Neurosci. 2012;7(1):11–22. Epub 2012/01/10. 10.1093/scan/nsr093 ; PubMed Central PMCID: PMC3252634. 22228751 PMC3252634

[2] Mauss IB, Levenson RW, McCarter L, Wilhelm FH, Gross JJ. The tie that binds? Coherence among emotion experience, behavior, and physiology. Emotion. 2005;5(2):175–90. Epub 2005/06/29. 10.1037/1528-3542.5.2.175 . 15982083

[3] Lazarus RS. Progress on a cognitive-motivational-relational theory of emotion. Am Psychol. 1991;46(8):819 1928936 10.1037//0003-066x.46.8.819

[4] Izard CE. The many meanings/aspects of emotion: Definitions, functions, activation, and regulation. Emotion Review. 2010;2(4):363–70.

[5] Scherer KR, Schorr A, Johnstone T. Appraisal processes in emotion: Theory, methods, research: Oxford University Press; 2001.

[6] Scherer KR. What are emotions? And how can they be measured? Social science information. 2005;44(4):695–729.

[7] Gray JR. Emotional modulation of cognitive control: Approach-withdrawal states double-dissociate spatial from verbal two-back task performance. J Exp Psychol Gen. 2001;130(3):436–52. Epub 2001/09/20. . 11561919 10.1037//0096-3445.130.3.436

[8] McRae K, Misra S, Prasad AK, Pereira SC, Gross JJ. Bottom-up and top-down emotion generation: Implications for emotion regulation. Soc Cogn Affect Neurosci. 2012;7(3):253–62. Epub 2011/02/08. 10.1093/scan/nsq103 ; PubMed Central PMCID: PMC3304475. 21296865 PMC3304475

[9] Gross JJ. The emerging field of emotion regulation: An integrative review. Review of general psychology. 1998;2(3):271.

[10] Ochsner KN, Gross JJ. The cognitive control of emotion. Trends Cogn Sci. 2005;9(5):242–9. 10.1016/j.tics.2005.03.010 . 15866151

[11] Phillips ML, Ladouceur CD, Drevets WC. A neural model of voluntary and automatic emotion regulation: Implications for understanding the pathophysiology and neurodevelopment of bipolar disorder. Mol Psychiatry. 2008;13(9):833–57. 10.1038/mp.2008.65 18574483 PMC2745893

[12] Gross JJ, John OP. Individual differences in two emotion regulation processes: Implications for affect, relationships, and well-being. J Pers Soc Psychol. 2003;85(2):348–62. 10.1037/0022-3514.85.2.348 12916575

[13] Abler B, Hofer C, Walter H, Erk S, Hoffmann H, Traue HC, et al Habitual emotion regulation strategies and depressive symptoms in healthy subjects predict fmri brain activation patterns related to major depression. Psychiatry Res. 2010;183(2):105–13. Epub 2010/07/16. 10.1016/j.pscychresns.2010.05.010 . 20630713

[14] Beauregard M, Paquette V, Levesque J. Dysfunction in the neural circuitry of emotional self-regulation in major depressive disorder. Neuroreport. 2006;17(8):843–6. Epub 2006/05/19. 10.1097/01.wnr.0000220132.32091.9f . 16708026

[15] Ziv M, Goldin PR, Jazaieri H, Hahn KS, Gross JJ. Emotion regulation in social anxiety disorder: Behavioral and neural responses to three socio-emotional tasks. Biol Mood Anxiety Disord. 2013;3(1):20 Epub 2014/02/13. 10.1186/2045-5380-3-20 ; PubMed Central PMCID: PMCPmc4029608. 24517388 PMC4029608

[16] Eippert F, Veit R, Weiskopf N, Erb M, Birbaumer N, Anders S. Regulation of emotional responses elicited by threat-related stimuli. Hum Brain Mapp. 2007;28(5):409–23. 10.1002/hbm.20291 . 17133391 PMC6871321

[17] Erk S, Mikschl A, Stier S, Ciaramidaro A, Gapp V, Weber B, et al Acute and sustained effects of cognitive emotion regulation in major depression. J Neurosci. 2010;30(47):15726–34. Epub 2010/11/26. doi: 30/47/15726 [pii] 10.1523/JNEUROSCI.1856-10.2010 . 21106812 PMC6633759

[18] Harenski CL, Hamann S. Neural correlates of regulating negative emotions related to moral violations. Neuroimage. 2006;30(1):313–24. Epub 2005/10/27. 10.1016/j.neuroimage.2005.09.034 . 16249098

[19] Hayes JP, Morey RA, Petty CM, Seth S, Smoski MJ, McCarthy G, et al Staying cool when things get hot: Emotion regulation modulates neural mechanisms of memory encoding. Front Hum Neurosci. 2010;4:230 Epub 2011/01/08. 10.3389/fnhum.2010.00230 ; PubMed Central PMCID: PMC3015134. 21212840 PMC3015134

[20] Herwig U, Kaffenberger T, Jancke L, Bruhl AB. Self-related awareness and emotion regulation. Neuroimage. 2010;50(2):734–41. 10.1016/j.neuroimage.2009.12.089 . 20045475

[21] Ochsner KN, Bunge SA, Gross JJ, Gabrieli JD. Rethinking feelings: An fmri study of the cognitive regulation of emotion. J Cogn Neurosci. 2002;14(8):1215–29. Epub 2002/12/24. 10.1162/089892902760807212 . 12495527

[22] Ochsner KN, Ray RD, Cooper JC, Robertson ER, Chopra S, Gabrieli JD, et al For better or for worse: Neural systems supporting the cognitive down- and up-regulation of negative emotion. Neuroimage. 2004;23(2):483–99. Epub 2004/10/19. 10.1016/j.neuroimage.2004.06.030 . 15488398

[23] Phan KL, Fitzgerald DA, Nathan PJ, Moore GJ, Uhde TW, Tancer ME. Neural substrates for voluntary suppression of negative affect: A functional magnetic resonance imaging study. Biol Psychiatry. 2005;57(3):210–9. Epub 2005/02/05. doi: S0006-3223(04)01110-2 [pii] 10.1016/j.biopsych.2004.10.030 . 15691521

[24] Kalisch R, Wiech K, Herrmann K, Dolan RJ. Neural correlates of self-distraction from anxiety and a process model of cognitive emotion regulation. J Cogn Neurosci. 2006;18(8):1266–76. Epub 2006/07/25. 10.1162/jocn.2006.18.8.1266 ; PubMed Central PMCID: PMC2638061. 16859413 PMC2638061

[25] Gillath O, Bunge SA, Shaver PR, Wendelken C, Mikulincer M. Attachment-style differences in the ability to suppress negative thoughts: Exploring the neural correlates. Neuroimage. 2005;28(4):835–47. 10.1016/j.neuroimage.2005.06.048 . 16087352

[26] Ochsner KN, Silvers JA, Buhle JT. Functional imaging studies of emotion regulation: A synthetic review and evolving model of the cognitive control of emotion. Ann N Y Acad Sci. 2012;1251:E1–24. Epub 2012/10/03. 10.1111/j.1749-6632.2012.06751.x . 23025352 PMC4133790

[27] Mak AK, Hu ZG, Zhang JX, Xiao ZW, Lee TM. Neural correlates of regulation of positive and negative emotions: An fmri study. Neurosci Lett. 2009;457(2):101–6. Epub 2009/05/12. 10.1016/j.neulet.2009.03.094 . 19429172

[28] Schmeichel BJ, Volokhov RN, Demaree HA. Working memory capacity and the self-regulation of emotional expression and experience. J Pers Soc Psychol. 2008;95(6):1526–40. Epub 2008/11/26. 10.1037/a0013345 . 19025300

[29] Miyake A, Shah P. Models of working memory: Mechanisms of active maintenance and executive control: Cambridge University Press; 1999.

[30] Baddeley AD, Hitch G. Working memory. Psychology of learning and motivation. 1974;8:47–89.

[31] Ochsner KN, Gross JJ. Cognitive emotion regulation: Insights from social cognitive and affective neuroscience. Current directions in psychological science. 2008;17(2):153–8. 10.1111/j.1467-8721.2008.00566.x 25425765 PMC4241349

[32] Kalisch R. The functional neuroanatomy of reappraisal: Time matters. Neuroscience & Biobehavioral Reviews. 2009;33(8):1215–26. 19539645 10.1016/j.neubiorev.2009.06.003

[33] Frank DW, Dewitt M, Hudgens-Haney M, Schaeffer DJ, Ball BH, Schwarz NF, et al Emotion regulation: Quantitative meta-analysis of functional activation and deactivation. Neurosci Biobehav Rev. 2014;45:202–11. Epub 2014/07/02. 10.1016/j.neubiorev.2014.06.010 . 24984244

[34] Kohn N, Eickhoff SB, Scheller M, Laird AR, Fox PT, Habel U. Neural network of cognitive emotion regulation—an ale meta-analysis and macm analysis. Neuroimage. 2014;87:345–55. Epub 2013/11/14. 10.1016/j.neuroimage.2013.11.001 ; PubMed Central PMCID: PMCPmc4801480. 24220041 PMC4801480

[35] Rottschy C, Langner R, Dogan I, Reetz K, Laird AR, Schulz JB, et al Modelling neural correlates of working memory: a coordinate-based meta-analysis. NeuroImage. 2012;60(1):830–46. Epub 2011/12/20. 10.1016/j.neuroimage.2011.11.050 ; PubMed Central PMCID: PMCPMC3288533. 22178808 PMC3288533

[36] Wager TD, Smith EE. Neuroimaging studies of working memory: a meta-analysis. Cognitive, affective & behavioral neuroscience. 2003;3(4):255–74. Epub 2004/03/26. . 15040547 10.3758/cabn.3.4.255

[37] Aron AR, Fletcher PC, Bullmore ET, Sahakian BJ, Robbins TW. Stop-signal inhibition disrupted by damage to right inferior frontal gyrus in humans. Nat Neurosci. 2003;6(2):115–6. Epub 2003/01/22. 10.1038/nn1003 . 12536210

[38] Rubia K, Russell T, Overmeyer S, Brammer MJ, Bullmore ET, Sharma T, et al Mapping motor inhibition: conjunctive brain activations across different versions of go/no-go and stop tasks. NeuroImage. 2001;13(2):250–61. Epub 2001/02/13. 10.1006/nimg.2000.0685 . 11162266

[39] Grecucci A, Giorgetta C, Bonini N, Sanfey AG. Reappraising social emotions: The role of inferior frontal gyrus, temporo-parietal junction and insula in interpersonal emotion regulation. Front Hum Neurosci. 2013;7:523 Epub 2013/09/13. 10.3389/fnhum.2013.00523 ; PubMed Central PMCID: PMC3759791. 24027512 PMC3759791

[40] Winecoff A, Labar KS, Madden DJ, Cabeza R, Huettel SA. Cognitive and neural contributors to emotion regulation in aging. Soc Cogn Affect Neurosci. 2011;6(2):165–76. Epub 2010/04/14. 10.1093/scan/nsq030 ; PubMed Central PMCID: PMC3073384. 20385663 PMC3073384

[41] Kerns JG, Cohen JD, MacDonald AW, Cho RY, Stenger VA, Carter CS. Anterior cingulate conflict monitoring and adjustments in control. Science (New York, NY). 2004;303(5660):1023–6. 10.1126/science.1089910 14963333

[42] Northoff G, Bermpohl F. Cortical midline structures and the self. Trends Cogn Sci. 2004;8(3):102–7. Epub 2004/08/11. 10.1016/j.tics.2004.01.004 . 15301749

[43] Pessoa L. On the relationship between emotion and cognition. Nature reviews neuroscience. 2008;9(2):148–58. 10.1038/nrn2317 18209732

[44] Jamadar SD, Fielding J, Egan GF. Quantitative meta-analysis of fMRI and PET studies reveals consistent activation in fronto-striatal-parietal regions and cerebellum during antisaccades and prosaccades. Front Psychol. 2013;4:749 10.3389/fpsyg.2013.00749 ; PubMed Central PMCID: PMCPMC3797465. 24137150 PMC3797465

[45] Schweizer S, Hampshire A, Dalgleish T. Extending brain-training to the affective domain: Increasing cognitive and affective executive control through emotional working memory training. PLoS ONE. 2011;6(9):e24372 10.1371/journal.pone.0024372 ; PubMed Central PMCID: PMCPMC3176229. 21949712 PMC3176229

[46] Eickhoff SB, Bzdok D, Laird AR, Kurth F, Fox PT. Activation likelihood estimation meta-analysis revisited. Neuroimage. 2012;59(3):2349–61. Epub 2011/10/04. 10.1016/j.neuroimage.2011.09.017 ; PubMed Central PMCID: PMCPMC3254820. 21963913 PMC3254820

[47] Turkeltaub PE, Eickhoff SB, Laird AR, Fox M, Wiener M, Fox P. Minimizing within-experiment and within-group effects in Activation Likelihood Estimation meta-analyses. Hum Brain Mapp. 2012;33(1):1–13. Epub 2011/02/10. 10.1002/hbm.21186 ; PubMed Central PMCID: PMCPMC4791073. 21305667 PMC4791073

[48] Gross JJ, Richards JM, John OP. Emotion regulation in everyday life. Emotion regulation in couples and families: Pathways to dysfunction and health. 2006;2006:13–35.

[49] Morris JA, Leclerc CM, Kensinger EA. Effects of valence and divided attention on cognitive reappraisal processes. Soc Cogn Affect Neurosci. 2014;9(12):1952–61. 10.1093/scan/nsu004 ; PubMed Central PMCID: PMCPMC4249474. 24493837 PMC4249474

[50] Kim SH, Hamann S. Neural correlates of positive and negative emotion regulation. J Cogn Neurosci. 2007;19(5):776–98. Epub 2007/05/10. 10.1162/jocn.2007.19.5.776 . 17488204

[51] Moher D, Shamseer L, Clarke M, Ghersi D, Liberati A, Petticrew M, et al Preferred reporting items for systematic review and meta-analysis protocols (prisma-p) 2015 statement. Systematic Reviews. 2015;4:1 10.1186/2046-4053-4-1 ; PubMed Central PMCID: PMCPMC4320440. 25554246 PMC4320440

[52] Talairach J, Tournoux P. Co-planar stereotaxic atlas of the human brain. 3-Dimensional proportional system: an approach to cerebral imaging. 1988.

[53] Lang PJ, Greenwald MK, Bradley MM, Hamm AO. Looking at pictures: Affective, facial, visceral, and behavioral reactions. Psychophysiology. 1993;30(3):261–73. 8497555 10.1111/j.1469-8986.1993.tb03352.x

[54] Bai L, Ma H, Huang Y-X. The Development of Native Chinese Affective Picture System-A pretest in 46 College Students. Chinese Mental Health Journal. 2005;11:000.

[55] Tottenham N, Tanaka JW, Leon AC, McCarry T, Nurse M, Hare TA, et al The NimStim set of facial expressions: judgments from untrained research participants. Psychiatry Res. 2009;168(3):242–9. 10.1016/j.psychres.2008.05.006 19564050 PMC3474329

[56] Lundqvist D, Flykt A, Öhman A. The Karolinska directed emotional faces (KDEF). CD ROM from Department of Clinical Neuroscience, Psychology section, Karolinska Institutet 1998;(1998).

[57] Rodriguez-Jimenez R, Avila C, Garcia-Navarro C, Bagney A, Aragon AM, Ventura-Campos N, et al Differential dorsolateral prefrontal cortex activation during a verbal n-back task according to sensory modality. Behavioural brain research. 2009;205(1):299–302. Epub 2009/08/29. 10.1016/j.bbr.2009.08.022 . 19712703

[58] Ricciardi E, Bonino D, Gentili C, Sani L, Pietrini P, Vecchi T. Neural correlates of spatial working memory in humans: a functional magnetic resonance imaging study comparing visual and tactile processes. Neuroscience. 2006;139(1):339–49. 10.1016/j.neuroscience.2005.08.045 . 16324793

[59] Eickhoff SB, Nichols TE, Laird AR, Hoffstaedter F, Amunts K, Fox PT, et al Behavior, sensitivity, and power of activation likelihood estimation characterized by massive empirical simulation. Neuroimage. 2016;137:70–85. 10.1016/j.neuroimage.2016.04.072 ; PubMed Central PMCID: PMCPMC4981641. 27179606 PMC4981641

[60] Cox RW. AFNI: software for analysis and visualization of functional magnetic resonance neuroimages. Comput Biomed Res. 1996;29(3):162–73. Epub 1996/06/01. . 8812068 10.1006/cbmr.1996.0014

[61] Quirk GJ, Beer JS. Prefrontal involvement in the regulation of emotion: Convergence of rat and human studies. Curr Opin Neurobiol. 2006;16(6):723–7. 10.1016/j.conb.2006.07.004 . 17084617

[62] Koechlin E, Jubault T. Broca's area and the hierarchical organization of human behavior. Neuron. 2006;50(6):963–74. Epub 2006/06/15. 10.1016/j.neuron.2006.05.017 . 16772176

[63] Koechlin E, Ody C, Kouneiher F. The architecture of cognitive control in the human prefrontal cortex. Science (New York, NY). 2003;302(5648):1181–5. Epub 2003/11/15. 10.1126/science.1088545 . 14615530

[64] Burgess PW, Veitch E, de Lacy Costello A, Shallice T. The cognitive and neuroanatomical correlates of multitasking. Neuropsychologia. 2000;38(6):848–63. . 10689059 10.1016/s0028-3932(99)00134-7

[65] Seeley WW, Menon V, Schatzberg AF, Keller J, Glover GH, Kenna H, et al Dissociable intrinsic connectivity networks for salience processing and executive control. J Neurosci. 2007;27(9):2349–56. 10.1523/JNEUROSCI.5587-06.2007 ; PubMed Central PMCID: PMCPMC2680293. 17329432 PMC2680293

[66] Sheffield JM, Repovs G, Harms MP, Carter CS, Gold JM, MacDonald AW 3rd, et al Fronto-parietal and cingulo-opercular network integrity and cognition in health and schizophrenia. Neuropsychologia. 2015;73:82–93. 10.1016/j.neuropsychologia.2015.05.006 ; PubMed Central PMCID: PMCPMC4505838. 25979608 PMC4505838

[67] Monks PJ, Thompson JM, Bullmore ET, Suckling J, Brammer MJ, Williams SC, et al A functional MRI study of working memory task in euthymic bipolar disorder: evidence for task-specific dysfunction. Bipolar Disord. 2004;6(6):550–64. 10.1111/j.1399-5618.2004.00147.x . 15541071

[68] Nebel K, Wiese H, Stude P, de Greiff A, Diener HC, Keidel M. On the neural basis of focused and divided attention. Brain research Cognitive brain research. 2005;25(3):760–76. Epub 2005/12/13. 10.1016/j.cogbrainres.2005.09.011 . 16337110

[69] Honey GD, Bullmore ET, Sharma T. Prolonged reaction time to a verbal working memory task predicts increased power of posterior parietal cortical activation. NeuroImage. 2000;12(5):495–503. 10.1006/nimg.2000.0624 . 11034857

[70] Christopoulos GI, Tobler PN, Bossaerts P, Dolan RJ, Schultz W. Neural correlates of value, risk, and risk aversion contributing to decision making under risk. The Journal of neuroscience: the official journal of the Society for Neuroscience. 2009;29(40):12574–83. 10.1523/JNEUROSCI.2614-09.2009 ; PubMed Central PMCID: PMCPMC2794196. 19812332 PMC2794196

[71] Lee TW, Josephs O, Dolan RJ, Critchley HD. Imitating expressions: Emotion-specific neural substrates in facial mimicry. Soc Cogn Affect Neurosci. 2006;1(2):122–35. Epub 2007/03/16. 10.1093/scan/nsl012 ; PubMed Central PMCID: PMC1820946. 17356686 PMC1820946

[72] Wang L, LaBar KS, Smoski M, Rosenthal MZ, Dolcos F, Lynch TR, et al Prefrontal mechanisms for executive control over emotional distraction are altered in major depression. Psychiatry Res. 2008;163(2):143–55. Epub 2008/05/06. doi: S0925-4927(07)00224-7 [pii] 10.1016/j.pscychresns.2007.10.004 ; PubMed Central PMCID: PMC2553159. 18455373 PMC2553159

[73] Kurth F, Zilles K, Fox PT, Laird AR, Eickhoff SB. A link between the systems: functional differentiation and integration within the human insula revealed by meta-analysis. Brain structure & function. 2010;214(5–6):519–34. Epub 2010/06/01. 10.1007/s00429-010-0255-z ; PubMed Central PMCID: PMCPMC4801482. 20512376 PMC4801482

[74] Chang LJ, Yarkoni T, Khaw MW, Sanfey AG. Decoding the role of the insula in human cognition: functional parcellation and large-scale reverse inference. Cereb Cortex. 2013;23(3):739–49. 10.1093/cercor/bhs065 ; PubMed Central PMCID: PMCPMC3563343. 22437053 PMC3563343

[75] Conway AR, Kane MJ, Engle RW. Working memory capacity and its relation to general intelligence. Trends Cogn Sci. 2003;7(12):547–52. . 14643371 10.1016/j.tics.2003.10.005

[76] Liakakis G, Nickel J, Seitz RJ. Diversity of the inferior frontal gyrus—a meta-analysis of neuroimaging studies. Behav Brain Res. 2011;225(1):341–7. Epub 2011/07/07. 10.1016/j.bbr.2011.06.022 . 21729721

[77] Baldo JV, Dronkers NF. The role of inferior parietal and inferior frontal cortex in working memory. Neuropsychology. 2006;20(5):529–38. 10.1037/0894-4105.20.5.529 . 16938015

[78] Silvers JA, Shu J, Hubbard AD, Weber J, Ochsner KN. Concurrent and lasting effects of emotion regulation on amygdala response in adolescence and young adulthood. Dev Sci. 2015;18(5):771–84. Epub 2014/12/03. 10.1111/desc.12260 ; PubMed Central PMCID: PMCPmc4459932. 25439326 PMC4459932

[79] Wager TD, Davidson ML, Hughes BL, Lindquist MA, Ochsner KN. Prefrontal-subcortical pathways mediating successful emotion regulation. Neuron. 2008;59(6):1037–50. Epub 2008/09/27. 10.1016/j.neuron.2008.09.006 S0896-6273(08)00753-8 ; PubMed Central PMCID: PMC2742320. 18817740 PMC2742320

[80] Hariri AR, Mattay VS, Tessitore A, Fera F, Weinberger DR. Neocortical modulation of the amygdala response to fearful stimuli. Biol Psychiatry. 2003;53(6):494–501. . 12644354 10.1016/s0006-3223(02)01786-9

[81] John OP, Gross JJ. Healthy and unhealthy emotion regulation: personality processes, individual differences, and life span development. J Pers. 2004;72(6):1301–33. 10.1111/j.1467-6494.2004.00298.x . 15509284

[82] Wilson FA, Scalaidhe S, Goldman-Rakic PS. Dissociation of object and spatial processing domains in primate prefrontal cortex. Science. 1993;260(5116):1955–8. 8316836 10.1126/science.8316836

[83] Cutini S, Scatturin P, Menon E, Bisiacchi PS, Gamberini L, Zorzi M, et al Selective activation of the superior frontal gyrus in task-switching: an event-related fNIRS study. Neuroimage. 2008;42(2):945–55. 10.1016/j.neuroimage.2008.05.013 . 18586525

[84] Pessoa L, Kastner S, Ungerleider LG. Attentional control of the processing of neutral and emotional stimuli. Cognitive Brain Research. 2002;15(1):31–45. 12433381 10.1016/s0926-6410(02)00214-8

[85] Goldberg II, Harel M, Malach R. When the brain loses its self: prefrontal inactivation during sensorimotor processing. Neuron. 2006;50(2):329–39. Epub 2006/04/25. 10.1016/j.neuron.2006.03.015 . 16630842

[86] Stewart JL, May AC, Tapert SF, Paulus MP. Hyperactivation to pleasant interoceptive stimuli characterizes the transition to stimulant addiction. Drug Alcohol Depend. 2015;154:264–70. 10.1016/j.drugalcdep.2015.07.009 ; PubMed Central PMCID: PMCPMC4537790. 26228575 PMC4537790

[87] Nachev P, Wydell H, O'Neill K, Husain M, Kennard C. The role of the pre-supplementary motor area in the control of action. NeuroImage. 2007;36 Suppl 2:T155–63. 10.1016/j.neuroimage.2007.03.034 ; PubMed Central PMCID: PMCPMC2648723. 17499162 PMC2648723

[88] Mega MS, Cummings JL, Salloway S, Malloy P. The limbic system: an anatomic, phylogenetic, and clinical perspective. J Neuropsychiatry Clin Neurosci. 1997;9(3):315–30. Epub 1997/07/01. 10.1176/jnp.9.3.315 . 9276837

[89] Banks SJ, Eddy KT, Angstadt M, Nathan PJ, Phan KL. Amygdala-frontal connectivity during emotion regulation. Soc Cogn Affect Neurosci. 2007;2(4):303–12. Epub 2008/11/06. 10.1093/scan/nsm029 ; PubMed Central PMCID: PMCPmc2566753. 18985136 PMC2566753

[90] Okon-Singer H, Hendler T, Pessoa L, Shackman AJ. The neurobiology of emotion-cognition interactions: Fundamental questions and strategies for future research. Front Hum Neurosci. 2015;9:58 Epub 2015/03/17. 10.3389/fnhum.2015.00058 ; PubMed Central PMCID: PMCPMC4344113. 25774129 PMC4344113

[91] Otto B, Misra S, Prasad A, McRae K. Functional overlap of top-down emotion regulation and generation: An fmri study identifying common neural substrates between cognitive reappraisal and cognitively generated emotions. Cogn Affect Behav Neurosci. 2014;14(3):923–38. Epub 2014/01/17. 10.3758/s13415-013-0240-0 . 24430617

[92] Vossel S, Geng JJ, Fink GR. Dorsal and ventral attention systems: distinct neural circuits but collaborative roles. The Neuroscientist: a review journal bringing neurobiology, neurology and psychiatry. 2014;20(2):150–9. 10.1177/1073858413494269 ; PubMed Central PMCID: PMCPMC4107817. 23835449 PMC4107817

[93] Decety J, Lamm C. The role of the right temporoparietal junction in social interaction: how low-level computational processes contribute to meta-cognition. The Neuroscientist: a review journal bringing neurobiology, neurology and psychiatry. 2007;13(6):580–93. Epub 2007/10/04. 10.1177/1073858407304654 . 17911216

[94] Krall SC, Rottschy C, Oberwelland E, Bzdok D, Fox PT, Eickhoff SB, et al The role of the right temporoparietal junction in attention and social interaction as revealed by ALE meta-analysis. Brain structure & function. 2015;220(2):587–604. Epub 2014/06/12. 10.1007/s00429-014-0803-z ; PubMed Central PMCID: PMCPMC4791048. 24915964 PMC4791048

[95] Mars RB, Sallet J, Schuffelgen U, Jbabdi S, Toni I, Rushworth MF. Connectivity-based subdivisions of the human right "temporoparietal junction area": evidence for different areas participating in different cortical networks. Cereb Cortex. 2012;22(8):1894–903. Epub 2011/10/01. 10.1093/cercor/bhr268 . 21955921

[96] Mesulam MM. From sensation to cognition. Brain. 1998;121 (Pt 6):1013–52. . 9648540 10.1093/brain/121.6.1013

[97] Dorfel D, Lamke JP, Hummel F, Wagner U, Erk S, Walter H. Common and differential neural networks of emotion regulation by detachment, reinterpretation, distraction, and expressive suppression: A comparative fmri investigation. Neuroimage. 2014;101:298–309. Epub 2014/07/06. 10.1016/j.neuroimage.2014.06.051 . 24993897

[98] Messina I, Bianco S, Sambin M, Viviani R. Executive and semantic processes in reappraisal of negative stimuli: Insights from a meta-analysis of neuroimaging studies. Front Psychol. 2015;6:956 Epub 2015/07/29. 10.3389/fpsyg.2015.00956 ; PubMed Central PMCID: PMCPMC4499672. 26217277 PMC4499672

[99] Brand AG. Hot cognition: Emotions and writing behavior. Journal of advanced composition. 1985:5–15.

[100] Downar J, Geraci J, Salomons TV, Dunlop K, Wheeler S, McAndrews MP, et al Anhedonia and reward-circuit connectivity distinguish nonresponders from responders to dorsomedial prefrontal repetitive transcranial magnetic stimulation in major depression. Biol Psychiatry. 2014;76(3):176–85. Epub 2014/01/07. 10.1016/j.biopsych.2013.10.026 S0006-3223(13)01034-2 . 24388670

[101] Levesque J, Eugene F, Joanette Y, Paquette V, Mensour B, Beaudoin G, et al Neural circuitry underlying voluntary suppression of sadness. Biol Psychiatry. 2003;53(6):502–10. Epub 2003/03/20. . 12644355 10.1016/s0006-3223(02)01817-6

[102] Poldrack RA. Can cognitive processes be inferred from neuroimaging data? Trends Cogn Sci. 2006;10(2):59–63. Epub 2006/01/13. 10.1016/j.tics.2005.12.004 . 16406760

[103] Hutzler F. Reverse inference is not a fallacy per se: cognitive processes can be inferred from functional imaging data. Neuroimage. 2014;84:1061–9. Epub 2013/01/15. 10.1016/j.neuroimage.2012.12.075 . 23313571

[104] Hill AC, Laird AR, Robinson JL. Gender differences in working memory networks: a BrainMap meta-analysis. Biol Psychol. 2014;102:18–29. Epub 2014/07/22. 10.1016/j.biopsycho.2014.06.008 ; PubMed Central PMCID: PMCPMC4157091. 25042764 PMC4157091

[105] Mak AK, Hu ZG, Zhang JX, Xiao Z, Lee TM. Sex-related differences in neural activity during emotion regulation. Neuropsychologia. 2009;47(13):2900–8. Epub 2009/06/27. 10.1016/j.neuropsychologia.2009.06.017 . 19555702

[106] Staudinger MR, Erk S, Walter H. Dorsolateral prefrontal cortex modulates striatal reward encoding during reappraisal of reward anticipation. Cereb Cortex. 2011;21(11):2578–88. 10.1093/cercor/bhr041 . 21459835

[107] Delgado MR, Nearing KI, Ledoux JE, Phelps EA. Neural circuitry underlying the regulation of conditioned fear and its relation to extinction. Neuron. 2008;59(5):829–38. 10.1016/j.neuron.2008.06.029 ; PubMed Central PMCID: PMCPMC3061554. 18786365 PMC3061554

[108] Campbell-Sills L, Simmons AN, Lovero KL, Rochlin AA, Paulus MP, Stein MB. Functioning of neural systems supporting emotion regulation in anxiety-prone individuals. Neuroimage. 2011;54(1):689–96. Epub 2010/08/03. 10.1016/j.neuroimage.2010.07.041 S1053-8119(10)01014-1 ; PubMed Central PMCID: PMC2962684. 20673804 PMC2962684

[109] Redick TS, Lindsey DR. Complex span and n-back measures of working memory: a meta-analysis. Psychon Bull Rev. 2013;20(6):1102–13. 10.3758/s13423-013-0453-9 . 23733330

