# Supplementary material for: Does emotion regulation engage the same neural circuit as working memory? A meta-analytical comparison between cognitive reappraisal of negative emotion and 2-back working memory task
Source: PLoS One. 2018 Sep 13;13(9):e0203753. doi: 10.1371/journal.pone.0203753 (PMC6136767; doi:10.1371/journal.pone.0203753)
Supplement: S1 File — The supplementary information contains three parts. The first two parts summarize the material excluded from the meta-analyses of ER and WM. The third part describes the results of the ER-WM conjunction analysis. (DOCX) [file pone.0203753.s001.docx]

# Supplementary Data

The supplementary document contains three parts. The first two parts summarize the material excluded from the meta-analyses of ER and WM. The references are listed in alphabetic order by the first author's surname. The reasons for exclusion are appended and some of them are coded as follows:

MS: mixed samples, ME: mixed emotions, MR: mixed up- and down-regulation; D: distraction or attention manipulation, T: thought suppression, E: expression suppression or manipulation, R: reward (e.g., financial gain) and loss or financial game design, C: conditioning and extinction design, Miss: missing pertinent data, P: regulation of pain. U: unclear about coordinate transformation. These codes are explained below:

Abbreviation: dynamic causal modeling (DCM), emotion regulation (ER), Montreal Neurological Institute (MNI), obsessive-compulsive disorder (OCD) , prefrontal cortex (PFC), positron emission tomography (PET), psychophysiological interaction (PPI), single-photon emission computed tomography (SPECT), transcranial magnetic stimulation (TMS), working memory (WM).

1. MS (mixed samples): the surveyed emotion reappraisal article reports the main effect of, for example, patient and healthy control groups. This study only included the brain activation maps of healthy groups.
2. ME (mixed emotions): the surveyed emotion reappraisal article reports a main effect of the regulation of positive and negative emotions. This study only included the brain activation maps of the down-regulation of negative emotions.
3. MR (mixed up- and down-regulation): the surveyed emotion reappraisal article reports a main effect of the up- and down-regulation of emotions. This study only included the brain activation maps of the down-regulation of negative emotions.
4. D (distraction or attention manipulation): the surveyed ER article adopts distraction or attention manipulation as the ER strategy. This study only included the brain activation maps of reappraisal as the ER strategy (i.e., reinterpretation and distancing).
5. T (thought suppression): the surveyed ER article adopts thought suppression as the ER strategy.
6. E (expression suppression or manipulation): the surveyed ER article adopts expression suppression or manipulation as the ER strategy.
7. R (reward and loss or financial game design): the surveyed article explores the ER of financial loss.
8. C (conditioning and extinction design): the surveyed article explores the ER of conditioning responses.
9. Miss (missing pertinent data): this code specifically refers to the surveyed emotion reappraisal articles that only report between group/condition comparisons, devoid of information on the ER of negative emotions.
10. P (regulation of pain): the surveyed article explores the ER of pain.
11. U (unclear about coordinate transformation): some papers (especially before 2007) used MNI coordinates as an approximation of Talairach coordinates, some papers adopted Brett's algorithm to convert the coordinates between MNI and Talairach space, and some papers adopted Lancaster's algorithm to convert the coordinates between MNI and Talairach space. This code was used when the information of coordinate conversion is not clear (e.g., declaring normalization to MNI standard space but reporting Talairach coordinates or not stating/referencing which algorithm was used to convert the coordinates from MNI to Talairach space, etc.).

The third part summarizes the results of the ER-WM conjunction analysis. The results of ER-WM conjunction analysis are highly concordant with the intersection of ER and WM activation maps that have already been illustrated in **S1 Fig** (in yellow) of the formal manuscript. To save article space, the conjunction results are summarized in this supplementary material.

# Part I: ER

Based on the literature search criteria, 456 articles were obtained for ER, and among them, 46 were included in the meta-analysis and 410 were excluded. Simple statistics of the various reasons for exclusion are described as follows (number of articles is depicted in parentheses): affective Stroop task (including emotion-counting Stroop and emotion/face-word Stroop; 10), age beyond the range 18-60 (22), attention task (2), automatic ER (1), C (9), cognitive task with emotion as a distractor/interference (not Stroop task; 8), comparisons between maintain and view (1), connectivity analysis (including PPI, DCM, path analysis; 9), correlation/regression analysis (6), controlled behavioral aggression paradigm (1), cued emotion conflict task (1), design regarding food (3), design of anticipation (10), design of autobiographical memory (1), design of comparisons between different traits (not reappraisal; 1), design of Cyberball game (1), decision-based cognitive dissonance paradigm (1), design about empathy (6), design about acupuncture (1), design about motivated reasoning (1), design about romantic love (1), design of appraising emotional and social material (3), design of attention to breath (1), design of auditory and visual stimuli congruence or incongruence for emotional content (1), design of cigarette-craving regulation (1), design of cognitive load and perception of attraction (1), design of a dictator game (1), design of emotion Go/NoGo (5), design of emotion induction (4), design of emotion priming (1), design of emotion words with personal relevance judgment (1), design of emotional working memory (4), design of feedback (1), design of gaze fixation (1), design of introspection (1), design of moral decision (1), design of observation of risk-taking actions (1), design of odorant-induced emotion and working memory task (1), design of shallow and deep emotion processing (1), design of shame and guilt induction (1), design of social interaction (5), design of symptom provocation of OCD (1), design of trauma experience and oxytocin (1), directed forgetting task (1), E (1), EEG/ERP study (3), emotion labeling/matching task (8), emotion rating in social interaction (1), emotion recognition task (6), ER by music listening (1), experimental design (1), exposure to infant's or child's cry/laughter/stress (4), exposure to stigma-related material (1), face matching task (2), functional near infrared spectroscopy (2), hemodynamic response function (1), imaging genetics (1), implicit processing of emotion (including emotional-face gender labeling; 5), linguistic study (1), ME (2), memory tasks (e.g., ER on subsequent memory; 3), mentalizing animation task (1), mindfulness task/training (3), Miss (26), motor response inhibition or flexibility task (including Go/NoGo and stop signal tasks; 5), MR (3), MS (15), neurofeedback study (10), no ER during scanning and the regulation was rated after the scan (1), P (15), paradigm of Prisoner's Dilemma Game (1), patient study (6), perception of emotional material (moderated by other factors/methods rather than the reappraisal tactics of reinterpretation and distancing; 35), perception of self-injurious material (2), perception of social interaction/material (5), psychological/psychosocial stress task (including Montreal imaging stress test, imagery stress; 4), R (10), regions of interest analysis (7), ER associated with autobiographical memory (incomparable with reappraisal; 2), regulation of emotion response during error commission (1), research protocol (1), resting state connectivity analysis (47), sample duplication (1), self-related task (including self-awareness, self-belief, self-criticism, self-praise, self-referential thinking; 10), shifted-attention emotion appraisal task (2), social regulation of emotion (1), stimuli: Adult Attachment Projective Picture System (1), structural connectivity analysis (1), T (3), task about prejudice (2), task of aversive imagery (1), task of emotional faces n-back task (1), task of up-regulation of emotion (2), task of viewing alcohol cues (1), task of face-word relevance rating (1), task-rest interactions in emotive paradigm (1), TMS study (3), and U (1).

It is notable that the excluded ER and WM (see Part II) studies are quite different in their constituents. For ER, a substantial portion of the exclusions originated from the diversity of experimental designs (237/410), such as the scenarios of pain, reward/loss, anticipation, conditioning, social interaction, psycho-social stress, interference resolution, and self-relevance conditions. All of these paradigms, despite their significance and importance, are well-known to engage neural networks different from those reported in conventional emotion research. To ensure comparability with WM, this study restricted the focus to well-established ER reappraisal tactics, which remarkably out-numbered other individual designs (i.e., number = 46 after excluding MS, MR, ME and Miss).

| Year | First Author | Reference | Reasons for exclusion from the meta-analysis |
| --- | --- | --- | --- |
| 2003 | Abel | [1] | emotion recognition and pharmaco-fMRI study |
| 2007 | Abler | [2] | emotion anticipation design |
| 2010 | Abler | [3] | comparison between different traits (e.g. high vs. low suppression score) not reappraisal |
| 2016 | Achterberg | [4] | automatic ER |
| 2009 | Akitsuki | [5] | P |
| 2014 | Albein-Urios | [6] | MS, contrast between patients (cocaine dependent) and controls |
| 2007 | Alia-Klein | [7] | linguistic and emotion perception |
| 2016 | Alkozei | [8] | emotion anticipation design |
| 2014 | Allard | [9] | connectivity analysis (psychophysiological interaction) |
| 2011 | Almeida | [10] | connectivity analysis (dynamic causal modeling) |
| 2014 | Altmann | [11] | linguistic study |
| 2013 | Amianto | [12] | resting state connectivity analysis |
| 2016 | Andari | [13] | face matching task |
| 2011 | Andreescu | [14] | age older than 60 y/o |
| 2013 | Anticevic | [15] | resting state connectivity analysis |
| 2016 | Arizmendi | [16] | emotion counting Stroop task |
| 2016 | Baczkowski | [17] | resting state connectivity analysis |
| 2013 | Ball | [18] | MS |
| 2014 | Bangen | [19] | emotion perception design |
| 2007 | Banks | [20] | connectivity analysis (psychophysiological interaction) |
| 2016 | Beauchamp | [21] | Miss, interaction analysis |
| 2014 | Belden | [22] | age younger than 18 (mean 10.5) |
| 2015 | Belden | [23] | age younger than 18 (mean 11.5) |
| 2012 | Benelli | [24] | processing of emotion words |
| 2012 | Bermingham | [25] | imaging genetics |
| 2010 | Berna | [26] | the impact of mood induction on pain perception |
| 2012 | Bertocci | [27] | design of emotional n-back |
| 2014 | Birn | [28] | resting state connectivity analysis |
| 2016 | Brady | [29] | resting state connectivity analysis |
| 2010 | Brassen | [30] | emotional faces as distractors in spatial-cueing paradigm |
| 2015 | Brown | [31] | design of emotion Go/NoGo |
| 2015 | Brown | [32] | design of emotion Go/NoGo |
| 2016 | Brown | [33] | design of emotion interference |
| 2012 | Bruce | [34] | design of emotion interference |
| 2013 | Bruhl | [35] | anticipation and perception of emotional stimuli |
| 2014 | Bruhl | [36] | neurofeedback study, focusing on amygdala |
| 2016 | Buchheim | [37] | stimuli: Adult Attachment Projective Picture System |
| 2014 | Burklund | [38] | age older than 60 y/o |
| 2015 | Burklund | [39] | emotion labeling design |
| 2011 | Campbell-Sills | [40] | MS: contrast between normal and the subjects with high anxiety trait |
| 2005 | Canli | [41] | imaging genetics and emotion perception |
| 2014 | Cao | [42] | resting state connectivity analysis |
| 2015 | Carlson | [43] | ER by music listening |
| 2016 | Cascio | [44] | self-affirmation task |
| 2015 | Caseras | [45] | n-back design with emotion distractors |
| 2014 | Chase | [46] | resting state connectivity analysis |
| 2015 | Chen | [47] | resting state connectivity analysis |
| 2007 | Cheng | [48] | P |
| 2013 | Cisler | [49] | pre-determined neural nodes of ER |
| 2016 | Cisler | [50] | patient study |
| 2013 | Clarke | [51] | design of threat anticipation and working memory load |
| 2014 | Clauss | [52] | design of anticipation of viewing fear faces |
| 2009 | Cojan | [53] | Go/NoGo task |
| 2010 | Coman | [54] | patient study (velocardiofacial syndrome ) |
| 2016 | Compere | [55] | a study of autobiographical memory |
| 2016 | Costanzo | [56] | emotion perception design |
| 2015 | Cremers | [57] | anticipation anxiety and resting state connectivity analysis |
| 2014 | Das | [58] | resting state connectivity analysis |
| 2014 | Davis | [59] | MS |
| 2012 | de Greck | [60] | task of empathy for anger |
| 2010 | De Raedt | [61] | combined HF-rTMS and fMRI study |
| 2014 | Dean | [62] | resting state connectivity analysis and patient study |
| 2008 | Delgado | [63] | C |
| 2011 | Demenescu | [64] | emotion perception task |
| 2015 | Denkova | [65] | task of autobiographical memory (distracted away from emotion) |
| 2014 | Denny | [66] | task of anticipation of ER |
| 2015 | Denny | [67] | Miss |
| 2015 | Derntl | [68] | task of empathy |
| 2013 | Deveney | [69] | age younger than 18 (mean 14.3), design of spatial curing and frustration induction |
| 2014 | DeWitt | [70] | resting state connectivity analysis |
| 2014 | Diers | [71] | task of up-regulation of emotion (by command "permit") |
| 2013 | Dillon | [72] | MS |
| 2014 | Dodhia | [73] | resting state connectivity analysis |
| 2014 | Doerig | [74] | task of self-criticism |
| 2011 | Dolcos | [75] | the paper is about experimental design |
| 2014 | Dolcos | [76] | design of different arousal levels and emotion perception |
| 2013 | Doll | [77] | resting state connectivity analysis |
| 2016 | Doll | [78] | design of attention to breath |
| 2015 | Dong | [79] | design about expertise in acupuncture |
| 2014 | Dorfel | [80] | Miss |
| 2015 | Dougherty | [81] | age younger than 18 (mean 6.7) |
| 2014 | Downar | [82] | rTMS and resting state connectivity analysis |
| 2009 | Drabant | [83] | emotion perception task |
| 2011 | Dziobek | [84] | empathy study |
| 2016 | Eack | [85] | resting state connectivity analysis |
| 2016 | Eack | [86] | task of emotional faces n-back task |
| 2016 | Emmerling | [87] | controlled behavioral aggression paradigm |
| 2015 | Engen | [88] | up-regulation of positive affect via compassion-meditation |
| 2015 | Enzi | [89] | R |
| 2016 | Enzi | [90] | task of empathy for pain and its interaction with angry face perception |
| 2006 | Erk | [91] | design of graded cognitive distraction during the anticipation of negative emotions |
| 2010 | Erk | [92] | design of ER on subsequent memory |
| 2013 | Ertl | [93] | EEG study |
| 2010 | Etkin | [94] | task of emotion conflict resolution (face-word Stroop) |
| 2011 | Farb | [95] | task of emotion perception |
| 2015 | Favre | [96] | task of emotion conflict resolution (face-word Stroop) |
| 2012 | Felder | [97] | depressive participants |
| 2015 | Feng | [98] | design of social interaction |
| 2013 | Firk | [99] | Miss, imaging genetics |
| 2010 | Fisher | [100] | design about romantic love |
| 2013 | Fox | [101] | P |
| 2016 | Frijling | [102] | design of trauma experience and oxytocin |
| 2012 | Fulwiler | [103] | resting state connectivity analysis |
| 2014 | Gaebler | [104] | MS |
| 2013 | Gaffrey | [105] | age younger than 18 (age 4-6) |
| 2013 | Gardener | [106] | ERP study |
| 2016 | Gasnier | [107] | attention task and patient study |
| 2012 | Gee | [108] | emotion labeling/matching design |
| 2013 | Gee | [109] | age younger than 18 (mean 11.6), design of emotional faces task |
| 2014 | Gee | [110] | age younger than 18 (mean 7.3), design of mother/stranger task |
| 2001 | George | [111] | task of viewing alcohol cues |
| 2015 | Gilam | [112] | R |
| 2005 | Gillath | [113] | T |
| 2015 | Giuliani | [114] | reappraisal of craved food (not negative emotion) |
| 2014 | Glaser | [115] | emotion-arousal word task |
| 2011 | Glotzbach | [116] | functional near infrared spectroscopy |
| 2014 | Goerlich-Dobre | [117] | task of emotional prosody categorization |
| 2009 | Goldin | [118] | MS |
| 2009 | Goldin | [119] | task of negative self-belief regulation |
| 2010 | Goldin | [120] | task of negative self-belief regulation |
| 2013 | Goldin | [121] | task of negative self-belief regulation |
| 2013 | Goldin | [122] | regions of interest analysis |
| 2014 | Goldin | [123] | task of negative self-belief regulation |
| 2009 | Goldstein | [124] | task of rewarded drug cue to cocaine addictives |
| 2015 | Gollan | [125] | task of perception of social scenes |
| 2014 | Goodman | [126] | design of repeated emotion perception |
| 2016 | Goodman | [127] | task of psychosocial stress |
| 2016 | Gorka | [128] | connectivity analysis (PPI) |
| 2011 | Grant | [129] | P |
| 2013 | Grecucci | [130] | R |
| 2013 | Grecucci | [131] | design of dictator game |
| 2014 | Greening | [132] | MS |
| 2014 | Gu | [133] | R |
| 2015 | Guhn | [134] | functional near-infrared spectroscopy |
| 2012 | Gyurak | [135] | design of appraising emotional and social material |
| 2010 | Habel | [136] | design of odorant-induced emotion and working memory task |
| 2014 | Hafeman | [137] | task of emotion perception |
| 2014 | Hallam | [138] | emotional dynamic faces task |
| 2014 | Hamm | [139] | resting state connectivity analysis |
| 2010 | Han | [140] | modified affective Stroop task |
| 2016 | Han | [141] | design of working memory task under emotional distraction |
| 2005 | Hare | [142] | emotional Go/NoGo task |
| 2008 | Hare | [143] | emotional Go/NoGo task |
| 2003 | Hariri | [144] | emotion recognition task |
| 2014 | Heissler | [145] | regions of interest analysis |
| 2013 | Heller | [146] | correlation/regression analysis |
| 2013 | Heller | [147] | patient study (depressive disorder ) |
| 2015 | Helmbold | [148] | R, modified Go/No-Go task |
| 2015 | Hennig-Fast | [149] | design of shame and guilt induction |
| 2013 | Hermann | [150] | task of emotion perception |
| 2014 | Hermann | [151] | C |
| 2016 | Hermann | [152] | correlation/regression analysis |
| 2016 | Herremans | [153] | design of TMS and alcohol-cue paradigm |
| 2007 | Herwig | [154] | Miss, between group comparison |
| 2010 | Herwig | [155] | design is introspection, not comparable with conventional ER/WM experiments |
| 2016 | Herwig | [156] | recognition of healthiness of food |
| 2013 | Holland | [157] | Miss, contras of up- and down-regulation of the emotions associated with negative autobiographical memories |
| 2013 | Holland | [158] | Miss, contras of up- and down-regulation of the emotions associated with negative autobiographical memories |
| 2013 | Holtmann | [159] | attention task with emotion as distractor |
| 2013 | Holzel | [160] | emotion recognition task and mindfulness training |
| 2010 | Hooker | [161] | no ER during scanning; the regulation is rated after scan |
| 2013 | Hulvershorn | [162] | facial emotion matching task |
| 2014 | Hulvershorn | [163] | resting state connectivity analysis |
| 2014 | Hwang | [164] | affective Stroop task |
| 2015 | Hwang | [165] | affective Stroop task |
| 2016 | Hwang | [166] | resting state connectivity analysis |
| 2011 | Ichikawa | [167] | regulation of emotion response during error commission, strategy not clear |
| 2013 | Ives-Deliperi | [168] | mindfulness task |
| 2013 | Jacob | [169] | Go/NoGo task |
| 2011 | Jarcho | [170] | decision-based cognitive dissonance paradigm |
| 2015 | Jarcho | [171] | social interaction and peer feedback on eating |
| 2016 | Javanbakht | [172] | regions of interest analysis |
| 2011 | Jeong | [173] | design of auditory and visual stimuli congruent or incongruent for emotional content |
| 2013 | Johnson | [174] | design of emotionally focused therapy on threat perception |
| 2007 | Johnstone | [175] | MS |
| 2016 | Kadosh | [176] | age younger than 18 (mean 11.6) |
| 2006 | Kalisch | [177] | ER by self-distraction |
| 2011 | Kanske | [178] | ME |
| 2012 | Kanske | [179] | MS |
| 2015 | Kanske | [180] | Miss, between group comparisons |
| 2015 | Kerr | [181] | R |
| 2016 | Keynan | [182] | neurofeedback study, focusing on amygdala |
| 2013 | Kienast | [183] | design of aversive emotional stimuli, dopamine and alcohol |
| 2013 | Kim | [184] | correlation/regression analysis |
| 2016 | Kim | [185] | task of face-word relevance rating |
| 2015 | Klucken | [186] | C |
| 2015 | Klucken | [187] | C and imaging genetics |
| 2016 | Klucken | [188] | C |
| 2012 | Klumpp | [189] | emotional face matching task |
| 2014 | Klumpp | [190] | resting state connectivity analysis |
| 2015 | Kogler | [191] | Montreal imaging stress test |
| 2014 | Kohn | [192] | design of mood induction |
| 2010 | Kraus | [193] | task about self-injurious behavior |
| 2009 | Krendl | [194] | task about prejudice |
| 2012 | Krendl | [195] | task about prejudice |
| 2011 | Kret | [196] | perception of threatening (fearful and angry) facial and bodily expressions |
| 2009 | Kross | [197] | "accept" to regulate negative autobiographical memories is uncomparable with active reappraisal |
| 2009 | Kumari | [198] | perception and anticipation of threat (electric shock) |
| 2011 | Ladouceur | [199] | emotional-face gender labeling tasks |
| 2013 | Ladouceur | [200] | emotional working memory task |
| 2012 | Laeger | [201] | perception of emotional words |
| 2014 | Lamke | [202] | task-rest interactions in emotive paradigm |
| 2012 | Lang | [203] | MS |
| 2012 | Laurent | [204] | maternal exposure to infant's cry |
| 2008 | Leclerc | [205] | emotion perception task |
| 2012 | Lee | [206] | connectivity analysis and individual ER |
| 2016 | Lemenager | [207] | self-retrieval paradigm |
| 2016 | Lepping | [208] | perception of emotional music |
| 2016 | Leutgeb | [209] | resting state connectivity analysis |
| 2004 | Lévesque | [210] | age younger than 18 (mean 9.9) |
| 2004 | Lévesque | [210] | U |
| 2007 | Li | [211] | stop signal task |
| 2016 | Li | [212] | resting state connectivity analysis and neurofeedback |
| 2016 | Li | [213] | real-time functional MRI neurofeedback |
| 2014 | Lueken | [214] | C and imaging genetics |
| 2016 | Luo | [215] | Cyberball game and imaging genetics |
| 2014 | Lutz | [216] | design of mindfulness training and cued expectation and perception of negative stimuli |
| 2016 | Lutz | [217] | design of mindful self-awareness and self-referential thinking |
| 2016 | Lutz | [218] | design of self-criticism and self-praise |
| 2016 | MacNamara | [219] | regions of interest analysis |
| 2012 | Majdandzic | [220] | design of moral decision |
| 2009 | Mak | [221] | Miss, ANOVA of gender difference |
| 2016 | Makovac | [222] | resting state connectivity analysis |
| 2015 | Manelis | [223] | emotion perception task |
| 2013 | Mansson | [224] | task of emotional faces perception |
| 2015 | Martucci | [225] | P |
| 2015 | Marusak | [226] | emotional conflict task |
| 2014 | Mascaro | [227] | paternal exposure to infant's cry |
| 2013 | Masten | [228] | design of experience and witness of social rejection |
| 2013 | Mazza | [229] | negative emotion perception |
| 2010 | McRae | [230] | correlation analysis |
| 2012 | McRae, 2012 | [231] | correlation/regression analysis |
| 2011 | Meyer | [232] | design of cognitive load and perception of attraction |
| 2012 | Micoulaud-Franchi | [233] | neurofeedback study |
| 2012 | Minkel | [234] | Miss |
| 2016 | Mocking | [235] | research protocol |
| 2007 | Mohanty | [236] | design of color-word and emotion-word distractors |
| 2016 | Morawetz | [237] | MR |
| 2016 | Morawetz | [238] | MR, effective connectivity analysis |
| 2016 | Morawetz | [239] | MR |
| 2006 | Moriguchi | [240] | mentalizing animation task |
| 2012 | Morris | [241] | Miss, between group comparisons |
| 2014 | Morris | [242] | Miss, divided attention design |
| 2013 | Moser | [243] | perception of mother-child interactions |
| 2015 | Moser | [244] | perception of social interaction films |
| 2015 | Moser | [245] | maternal perception of stressful child stimuli |
| 2014 | Moutsiana | [246] | Miss, focusing on up-regulation of positive affect |
| 2016 | Mukherjee | [247] | implicit processing of emotion |
| 2015 | Mulej Bratec | [248] | C, design of ER and prediction error |
| 2015 | Murakami | [249] | T, suppression of feeling |
| 2016 | Murphy | [250] | age younger than 18 (mean 11.3) |
| 2007 | Nelson | [251] | motor response flexibility task |
| 2009 | New | [252] | Miss |
| 2015 | Nicholson | [253] | resting state connectivity analysis |
| 2016 | Nicholson | [254] | resting state connectivity analysis and EEG feedback |
| 2010 | Niedtfeld | [255] | P |
| 2017 | Niedtfeld | [256] | P |
| 2014 | Olatunji | [257] | design of symptom provocation of OCD |
| 2015 | O'Neill | [258] | resting state connectivity analysis and theory of mind task |
| 2015 | Opel | [259] | R, card guessing paradigm |
| 2015 | Opialla | [260] | P and ER |
| 2010 | Ossewaarde | [261] | stress induction design |
| 2015 | Outhred | [262] | connectivity analysis (PPI) |
| 2016 | Outhred | [263] | connectivity analysis (PPI), imaging genetics |
| 2013 | Pannekoek | [264] | resting state connectivity analysis |
| 2016 | Papini | [265] | resting state connectivity analysis |
| 2014 | Paret | [266] | neurofeedback study |
| 2014 | Paret | [267] | emotion perception task |
| 2016 | Paret | [268] | neurofeedback study and functional connectivity analysis |
| 2016 | Paret | [269] | neurofeedback study and functional connectivity analysis |
| 2012 | Passarotti | [270] | emotional working memory task |
| 2011 | Payer | [271] | affect labeling/matching task |
| 2012 | Payer | [272] | regions of interest analysis |
| 2010 | Perlman | [273] | age younger than 18 (mean 8.2) |
| 2011 | Perlman | [274] | age younger than 18 (mean 8.2) |
| 2012 | Perlman | [275] | age younger than 18 (mean 15.1) |
| 2015 | Perlman | [276] | age younger than 18 (mean 7.7) |
| 2015 | Petrican | [277] | perception of nonverbal affective cues |
| 2001 | Phillips | [278] | perception of aversive scenes |
| 2011 | Pitskel | [279] | age younger than 18 (mean 13.0) |
| 2014 | Pitskel | [280] | age younger than 18 (mean 13.8) |
| 2015 | Platt | [281] | age younger than 18 (mean 15.6) |
| 2012 | Plener | [282] | perception to non-suicidal self-injury material |
| 2015 | Prehn | [283] | emotion rating in social interaction |
| 2013 | Preis | [284] | P |
| 2016 | Price | [285] | age younger than 18 (mean 10.7) |
| 2014 | Puetz | [286] | age younger than 18 (mean 10.6) |
| 2016 | Puetz | [287] | age younger than 18 (mean 12.5) |
| 2015 | Puglia | [288] | emotion perception task |
| 2015 | Radaelli | [289] | face-matching task |
| 2014 | Raij | [290] | exposure to stigma-related material |
| 2014 | Rauch | [291] | memory and recognition tasks |
| 2016 | Rausch | [292] | resting state connectivity analysis |
| 2014 | Raz | [293] | design of empathy |
| 2016 | Reidy | [294] | design of emotion perception and sleep deprivation |
| 2014 | Reinecke | [295] | Miss |
| 2015 | Reinecke | [296] | MS |
| 2015 | Reitz | [297] | resting state connectivity analysis |
| 2007 | Reske | [298] | design of emotion induction |
| 2016 | Rey | [299] | resting state connectivity analysis |
| 2010 | Richter | [300] | emotion perception task |
| 2012 | Riem | [301] | exposure to infant laughter |
| 2011 | Ritchey | [302] | design of shallow and deep emotion processing |
| 2011 | Ritchey | [303] | emotion perception task |
| 2015 | Rive | [304] | MS, ME |
| 2016 | Rive | [305] | resting state connectivity analysis |
| 2009 | Roelofs | [306] | social emotion task (approach vs. avoid) |
| 2016 | Romero-Rebollar | [307] | emotion perception task |
| 2012 | Rosales-Lagarde | [308] | emotion perception task |
| 2015 | Sadeh | [309] | Go/NoGo task |
| 2007 | Salomons | [310] | P |
| 2015 | Sanchez | [311] | emotion perception task |
| 2002 | Schaefer | [312] | comparison between maintain and view |
| 2015 | Scharmuller | [313] | P, connectivity analysis |
| 2015 | Scherpiet | [314] | Miss, between group comparisons |
| 2010 | Scheuerecker | [315] | emotion recognition task |
| 2009 | Schienle | [316] | task of aversive imagery |
| 2015 | Schneider-Hassloff | [317] | paradigm of Prisoner's Dilemma Game |
| 2011 | Schulze | [318] | MS |
| 2013 | Schweizer | [319] | emotional working memory task |
| 2016 | Schweizer | [320] | regions of interest analysis |
| 2004 | Schwerdtner | [321] | design of emotion induction |
| 2009 | Seiferth | [322] | emotion recognition task |
| 2016 | Seitz | [323] | attention network task |
| 2014 | Seo | [324] | ME |
| 2016 | Seo | [325] | design of imagery stress and alcohol cue |
| 2014 | Servaas | [326] | mood (worry) induction design |
| 2016 | Servaas | [327] | resting state connectivity analysis |
| 2016 | Shen | [328] | resting state connectivity analysis |
| 2015 | Shi | [329] | implicit processing of emotion |
| 2006 | Siegle | [330] | design of emotion words with personal relevance judgment |
| 2011 | Siegle | [331] | emotion identification and label of words |
| 2015 | Silvers | [332] | connectivity analysis (PPI, path analysis) |
| 2015 | Silvers | [333] | sample duplication (Silvers, SCAN 2015, similar design) |
| 2016 | Silvers | [334] | patient study |
| 2016 | Silvers | [335] | patient study |
| 2014 | Simon | [336] | aversive stimuli as distractors |
| 2014 | Singh | [337] | resting state connectivity analysis |
| 2015 | Sladky | [338] | facial emotion and object discrimination tasks |
| 2011 | Smoski | [339] | aversive stimuli as distractors |
| 2013 | Smoski | [340] | MS |
| 2015 | Smoski | [341] | Miss, MR (down-regulate vs. accept [up-regulate?]) |
| 2013 | Sokol-Hessner | [342] | R |
| 2015 | Song | [343] | resting state connectivity analysis |
| 2012 | Sripada | [344] | resting state connectivity analysis |
| 2013 | Sripada | [345] | resting state connectivity analysis |
| 2013 | Sripada | [346] | shifted-attention emotion appraisal task |
| 2013 | Sripada | [347] | shifted-attention emotion appraisal task |
| 2011 | Staudinger | [348] | R |
| 2013 | Stevens | [349] | emotion perception task |
| 2016 | Steward | [350] | Miss, between group comparisons |
| 2016 | Striepens | [350] | exposure to pictures of palatable food |
| 2016 | Sukhodolsky | [351] | Go/NoGo task, emotion perception task |
| 2012 | Surguladze | [352] | emotion perception task |
| 2014 | Swartz | [353] | implicit emotion processing task |
| 2014 | Swartz | [354] | implicit emotion processing task |
| 2014 | Tabibnia | [355] | connectivity analysis |
| 2014 | Tadayonnejad | [356] | resting state connectivity analysis |
| 2013 | Tamura | [357] | design of observation of risk-taking actions |
| 2016 | Tang | [358] | perception of social threat |
| 2013 | Tao | [359] | resting state connectivity analysis |
| 2006 | Taylor | [360] | emotion perception and label tasks |
| 2013 | Thomaes | [361] | subsequent memory paradigm of negative words |
| 2016 | Toazza | [362] | resting state connectivity analysis |
| 2013 | Torrisi | [363] | resting state connectivity analysis |
| 2013 | Torrisi | [364] | emotion label task |
| 2013 | Townsend | [365] | Miss, between group comparisons |
| 2012 | Tromp | [366] | structural connectivity analysis |
| 2015 | Uchida | [367] | resting state connectivity analysis |
| 2006 | Urry | [368] | Miss |
| 2009 | Urry | [369] | Miss, correlation with autonomic indices, ANOVA |
| 2015 | van den Stock | [370] | Miss, between group comparisons |
| 2016 | van der Horn | [371] | resting state connectivity analysis |
| 2007 | van Reekum | [372] | design of gaze fixation and ER |
| 2011 | Vanderhasselt | [373] | emotional Go/NoGo task |
| 2013 | Vanderhasselt | [374] | cued emotion conflict task |
| 2011 | Vrticka | [375] | Miss, contrast between ERs, not ER itself |
| 2012 | Vrticka | [376] | Miss |
| 2012 | Vrticka | [377] | design of appraising emotional and social material |
| 2013 | Vrticka | [378] | E |
| 2008 | Wager | [379] | correlation analysis |
| 2013 | Wagner | [380] | emotional scenes task and attention control task |
| 2015 | Wagner | [381] | paradigm of social sharing |
| 2014 | Waring | [382] | face-word emotion conflict task |
| 2010 | Warren | [383] | emotion-word Stroop task |
| 2016 | Weidt | [384] | emotion anticipation and perception |
| 2013 | Weng | [385] | design about empathy |
| 2006 | Westen | [386] | design about motivated reasoning |
| 2014 | Wheelock | [387] | C |
| 2008 | Wiech | [388] | P |
| 2014 | Wiech | [389] | P |
| 2015 | Woo | [390] | design of feedback |
| 2015 | Wood | [391] | C |
| 2016 | Wu | [392] | resting state connectivity analysis |
| 2016 | Xie | [393] | social regulation of emotion |
| 2013 | Xiong | [394] | Miss, between group comparisons |
| 2016 | Yang | [395] | directed forgetting task |
| 2016 | Yao | [396] | neurofeedback study, focusing on insula |
| 2015 | Yoon | [397] | design of emotion prime |
| 2014 | Yoshimura | [398] | P and anxiety regulation |
| 2014 | Yuan | [399] | resting state connectivity analysis |
| 2013 | Zhang | [400] | hemodynamic response function |
| 2013 | Zhang | [401] | Miss, between group comparisons |
| 2014 | Zhang | [402] | ERP study |
| 2014 | Zhang | [403] | resting state connectivity analysis |
| 2012 | Zhao | [404] | design of cigarette craving regulation |
| 2013 | Ziv | [405] | exposure to different negative socio-emotional stimuli |
| 2013 | Zotev | [406] | neurofeedback study, focusing on amygdala |
| 2016 | Zotev | [407] | neurofeedback study, focusing on amygdala |
| 2016 | Zou | [408] | connectivity analysis |

# Part II

Based on the literature search criteria, 406 articles were obtained for WM, and of these, 50 studies were included in the meta-analysis and 356 studies were excluded. Similar to Part I, the excluded references are listed in alphabetic order by the first author's name. The reasons for exclusion are appended and coded. Simple statistics of the various reasons for exclusions are described as follows (number of articles is depicted in parentheses): 2-back minus 1-back (not baseline; 2), age beyond the range 18-60 (40), auditory WM task (10), behavioral study (1), case study (6), combined working memory and dichotic-listening paradigm (1), connectivity analysis (including path analysis, 12), correlation/regression analysis (12), emotional or social variant of WM task (9), functional near infrared spectroscopy (4), incomplete report of coordinates (10), independent component analysis (9), graph theoretical approach (4), machine learning/classification (2), magnetoencephalography study (2), main interest in performance (1), methodology of fMRI analysis (2), Miss (141), modified Stroop task (1), MS (11), multi-variate analysis (3), patient study (9), permutation analysis for parametric effect (1), PET study (1), region of interest analyses (38), resting state analysis (1), sample duplication (2), SPECT study (1), subjects in nicotine withdrawal (1), subjects in sleep deprivation (1), tactile WM task (1), trend analysis, parametric effect (1), U (14), variant of face-matching WM task (1), and volumetric study (1).

Since n-back is a mature design that has been used to explore a broad range of neuro-psychiatric conditions, the WM studies in recent years tend to ignore the contrast of "2-back minus baseline" and only report the results of "between condition" or "between group" contrasts (140/356).

| Year | First Author | Endnote | Reasons for exclusion from the meta-analysis |
| --- | --- | --- | --- |
| 2014 | Abraham | [409] | Miss |
| 2014 | Ahluwalia | [410] | patient study |
| 2009 | Aloia | [411] | Miss, between condition contrast, region of interest analysis |
| 2006 | Andrews | [412] | region of interest analysis |
| 2009 | Archbold | [413] | patient study |
| 2013 | Ashare | [414] | Miss |
| 2013 | Ashare | [415] | Miss, between condition comparison, proof of concept study |
| 2009 | Axmacher | [416] | Miss, between condition and between group comparisons |
| 2011 | Bakshi | [417] | Miss, between group comparison, connectivity analysis |
| 2003 | Barch | [418] | Miss, between group comparison |
| 2009 | Barnes | [419] | Miss, F-test of 1-, 2-back and baseline |
| 2010 | Bayerl | [420] | Miss, between group comparison |
| 2011 | Becerril | [421] | region of interest analysis, between group comparison |
| 2012 | Bechtel | [422] | age younger than 18 |
| 2014 | Bedard | [423] | age younger than 18 |
| 2007 | Beneventi | [424] | emotional variant of WM task |
| 2010 | Beneventi | [425] | age younger than 18 |
| 2010 | Beneventi | [426] | age younger than 18 |
| 2011 | Bengtsson | [427] | Miss, between group comparison |
| 2013 | Bennett | [428] | age younger than 18 |
| 2013 | Bernal-Casas | [429] | connectivity analysis |
| 2004 | Bertolino | [430] | patient study |
| 2010 | Bertolino | [431] | Miss |
| 2013 | Bilek | [432] | connectivity analysis |
| 2014 | Bleich-Cohen | [433] | machine learning |
| 2008 | Blokland | [434] | region of interest analysis |
| 2014 | Blokland | [435] | region of interest analysis |
| 2008 | Brahmbhatt | [436] | region of interest analysis |
| 2014 | Brandt | [437] | independent component analysis |
| 2007 | Brechmann | [438] | auditory WM task |
| 2011 | Brookes | [439] | magnetoencephalography study |
| 2009 | Broome | [440] | Miss, between group comparison |
| 2012 | Brown | [441] | Miss, between group comparison |
| 2011 | Burgess | [442] | correlation analysis |
| 2013 | Burzynska | [443] | multi-modal and multi-variate analysis |
| 2000 | Callicott | [444] | Miss, between group comparison |
| 2003 | Callicott | [445] | Miss, between group comparison |
| 2003 | Callicott | [446] | Miss, between group comparison |
| 2013 | Campanella | [447] | Miss, between group comparison |
| 2014 | Cao | [448] | graph theoretical approach |
| 2006 | Caseras | [449] | trend analysis, parametric effect |
| 2009 | Castronovo | [450] | MS, Miss, between group comparison |
| 2008 | Cerasa | [451] | U |
| 2004 | Chang | [452] | age younger than 18 |
| 2015 | Chantiluke | [453] | age younger than 18 |
| 2012 | Chao | [454] | Miss |
| 2014 | Charlet | [455] | region of interest analysis |
| 2013 | Chen | [456] | Miss, between group comparison |
| 2014 | Chen | [457] | Miss, between group comparison |
| 2005 | Choo | [458] | Miss, only 2-back - 1-back |
| 2010 | Christ | [459] | Miss, between group comparison |
| 2006 | Ciesielski | [460] | U |
| 2015 | Colizzi | [461] | Miss, between group comparison |
| 2012 | Colorado | [462] | Miss, between group comparison |
| 2012 | Cousijn | [463] | Miss, between group comparison |
| 2014 | Cousijn | [464] | Miss, between group comparison |
| 2014 | Cousijn | [465] | Miss, between group comparison |
| 2013 | Creswell | [466] | Miss, between group comparison |
| 2012 | Cservenka | [467] | age younger than 18 |
| 2014 | Cubillo | [468] | Miss, between group comparison |
| 2015 | Daamen | [469] | Miss, between group comparison |
| 2015 | D'Aiuto | [470] | Miss, between group comparison |
| 2003 | Daumann | [471] | Miss, between group comparison |
| 2004 | Daumann | [472] | Miss, between group comparison |
| 2010 | de Frias | [473] | Miss, between group comparison |
| 2014 | de Vries | [474] | MS |
| 2014 | Dettwiler | [475] | Miss, between group comparison |
| 2014 | Di Giorgio | [476] | Miss, comparison between different genotypes |
| 2007 | DiFrancesco | [477] | age younger than 18 |
| 2011 | Diwadkar | [478] | age younger than 18 |
| 2013 | Diwadkar | [479] | age younger than 18 |
| 2008 | Dohnel | [480] | emotional variant of WM task |
| 2013 | Drummond | [481] | region of interest analysis |
| 2001 | Druzgal | [482] | U |
| 2008 | Dumas | [483] | Miss, between group comparison |
| 2010 | Dumas | [484] | Miss, between group comparison |
| 2010 | Dumas | [484] | Miss, between group comparison |
| 2013 | Dumas | [485] | Miss, between group comparison |
| 2015 | Dumas | [486] | age older than 60 |
| 2009 | Elsabagh | [487] | Miss, between group comparison, correlation analysis |
| 2007 | Elzinga | [488] | U |
| 2012 | Epperson | [489] | region of interest analysis |
| 2002 | Ernst | [490] | region of interest analysis |
| 2006 | Esposito | [491] | independent component analysis |
| 2009 | Esposito | [492] | region of interest analysis |
| 2009 | Esposito | [493] | case study |
| 2014 | Esslinger | [494] | Miss, between condition comparison |
| 2011 | Ettinger | [495] | Miss, between group comparison |
| 2013 | Faget-Agius | [496] | Miss, between group comparison |
| 2014 | Falcone | [497] | Miss, between group comparison |
| 2012 | Fatjo-Vilas | [498] | Miss, between group comparison |
| 2013 | Fernandez-Corcuera | [499] | incomplete report of coordinates |
| 2008 | Fitzgerald | [500] | Miss, between group comparison |
| 2008 | Fitzgibbon | [501] | region of interest analysis |
| 2007 | Forn | [502] | auditory WM task |
| 2008 | Frangou | [503] | U |
| 2010 | Fusar-Poli | [504] | MS |
| 2011 | Fusar-Poli | [505] | MS |
| 2013 | Gaudeau-Bosma | [506] | Miss, between condition comparison |
| 2014 | Geerligs | [507] | independent component analysis |
| 2014 | Gelao | [508] | Miss, between group comparison |
| 2007 | George | [509] | Miss, between group comparison |
| 2013 | Georgiou-Karistianis | [510] | Miss, between group comparison |
| 2014 | Georgiou-Karistianis | [511] | Miss, between group comparison |
| 2011 | Ginestet | [512] | connectivity and graph analysis |
| 2010 | Gonzales | [513] | region of interest analysis |
| 2010 | Gonzales | [514] | region of interest analysis |
| 2011 | Gonzales | [515] | region of interest analysis |
| 2014 | Gonzales | [516] | region of interest analysis |
| 2014 | Gonzales | [517] | region of interest analysis |
| 2014 | Gordon | [518] | connectivity analysis |
| 2015 | Gordon | [519] | connectivity analysis |
| 2014 | Greve | [520] | incomplete report of coordinates |
| 2014 | Griebe | [521] | age older than 60 |
| 2013 | Griffiths | [522] | age younger than 18 |
| 2013 | Gropman | [523] | 2-back - 1-back (not baseline) |
| 2012 | Guerrero-Pedraza | [524] | Miss, between group comparison |
| 2013 | Guse | [525] | Miss, between condition comparison |
| 2010 | Habel | [136] | Miss, between group comparison |
| 2001 | Haberecht | [526] | age younger than 18 |
| 2014 | Haeussinger | [527] | region of interest analysis |
| 2008 | Haldane | [528] | patient study |
| 2007 | Haley | [529] | age older than 60, patient study |
| 2013 | Haller | [530] | age older than 60 |
| 2014 | Haller | [531] | Miss, between group comparison |
| 2015 | Harding | [532] | connectivity analysis |
| 2015 | Harvey | [533] | case study |
| 2009 | Hautzel | [534] | region of interest analysis |
| 2014 | Havermans | [535] | Miss, between condition comparison |
| 2015 | He | [536] | Miss, between group comparison |
| 2014 | Heinzel | [537] | region of interest analysis |
| 2011 | Henckens | [538] | Miss, drug condition included in main effect |
| 2008 | Hirano | [539] | Miss, between condition comparison |
| 2002 | Honey | [540] | path analysis |
| 2002 | Honey | [541] | sample duplication (Honey, Bullmore and Sharma, 2000) |
| 2011 | Hoth | [542] | Miss, between group comparison |
| 2013 | Hu | [543] | region of interest analysis |
| 2008 | Hurt | [544] | age younger than 18 |
| 2007 | Ikeda | [545] | Miss |
| 2004 | Jacobsen | [546] | auditory WM task |
| 2014 | Jacola | [547] | main interest in performance |
| 2009 | Jansen | [548] | Miss, between group comparison |
| 2000 | Jansma | [549] | region of interest analysis |
| 2013 | Kalmady | [550] | correlation analysis |
| 2014 | Kalpakidou | [551] | Miss, between group comparison |
| 2009 | Karch | [552] | Miss, between group comparison |
| 2011 | Kasahara | [553] | Miss, does not differentiate activation and deactivation |
| 2014 | Kearney-Ramos | [554] | independent component analysis |
| 2012 | Kerestes | [555] | emotional variant of WM task |
| 2006 | Kim | [556] | U |
| 2012 | Kim | [557] | Miss, between group comparison |
| 2014 | Kim | [558] | modified Stroop task |
| 2010 | Klemen | [559] | auditory WM task |
| 2006 | Knops | [560] | 2-back - 1-back (not baseline) |
| 2013 | Ko | [561] | U |
| 2009 | Kobel | [562] | age younger than 18 |
| 2007 | Koch | [563] | Miss, between condition/group comparisons |
| 2012 | Koch | [564] | Miss, between group comparison |
| 2015 | Kodama | [565] | correlation analysis |
| 2013 | Koike | [566] | functional near infrared spectroscopy |
| 2014 | Koppe | [567] | Miss, between condition comparisons (design of 1-back) |
| 2013 | Korsnes | [568] | Miss, only 2-back - 1-back |
| 2005 | Koshino | [569] | region of interest analysis |
| 2008 | Krug | [570] | Miss, between group comparison |
| 2003 | Kumari | [571] | U |
| 2006 | Kumari | [572] | patient study |
| 2006 | Kumari | [573] | U |
| 2009 | Kumari | [574] | correlation analysis |
| 2001 | Kwon | [575] | age younger than 18 |
| 2002 | Kwon | [576] | correlation analysis |
| 2013 | Landin-Romero | [577] | case study |
| 2015 | Landin-Romero | [578] | Miss, between group comparison |
| 2014 | Lao-Kaim | [579] | Miss, between group comparison |
| 2013 | Lee | [580] | age older than 60 |
| 2005 | Lenartowicz | [581] | multi-variate analysis |
| 2011 | Leung | [582] | auditory WM task |
| 2010 | Li | [583] | functional near infrared spectroscopy |
| 2013 | Li | [584] | volumetry study |
| 2014 | Li | [585] | region of interest and between condition analyses |
| 2014 | Li | [586] | age younger than 18 |
| 2013 | Liang | [587] | graph theoretical approach |
| 2012 | Liao | [588] | Miss, between group comparison |
| 2009 | Libertus | [589] | Miss, between condition/group comparisons |
| 2014 | Longo | [590] | Miss |
| 2009 | Loughead | [591] | Miss, parametric model (2*3-back + 1*2-back - 1*1-back -2*0-back) |
| 2010 | Loughead | [592] | region of interest analysis |
| 2015 | Loughead | [593] | region of interest analysis |
| 2012 | Luckhoo | [594] | magnetoencephalography study |
| 2008 | Ludwig | [595] | SPECT study |
| 2014 | Luo | [596] | emotional variant of WM task |
| 2014 | Lv | [597] | Miss, between group comparison |
| 2014 | Madre | [598] | Miss, between group comparison |
| 2015 | Manelis | [599] | permutation analysis for parametric effect |
| 2010 | Mannie | [600] | Miss, between group comparison |
| 2009 | Marklund | [601] | Miss, between group comparison |
| 2010 | Markov | [602] | Miss, between group comparison |
| 2008 | Marquand | [603] | U |
| 2011 | Marrelec | [604] | Miss, focus on default-mode network |
| 2000 | Martinkauppi | [605] | auditory WM task |
| 2012 | Massat | [606] | age younger than 18 |
| 2007 | Matsuo | [607] | Miss, focus on frontal region |
| 2006 | Mattay | [608] | Miss, between group comparison |
| 2001 | McAllister | [609] | Miss, between group comparison |
| 2012 | McDonald | [610] | Miss, between group comparison |
| 2010 | McGeown | [611] | age older than 60 |
| 2012 | Medaglia | [612] | MS (main effect reported) |
| 2006 | Meisenzahl | [613] | U |
| 2007 | Menzies | [614] | incomplete report of coordinates |
| 2013 | Meusel | [615] | Miss, between group and condition comparisons |
| 2015 | Migo | [616] | age older than 60 |
| 2008 | Miskowiak | [617] | Miss, between condition comparison |
| 2007 | Mitchell | [618] | emotional variant of WM task |
| 2011 | Nagel | [619] | Miss, only 3-back - 1-back |
| 2013 | Nagel | [620] | age younger than 18 |
| 2009 | Nakao | [621] | Miss, between group comparison |
| 2011 | Nejad | [622] | MS |
| 2013 | Nejad | [623] | machine classification |
| 2007 | Newsome | [624] | age younger than 18 |
| 2007 | Newsome | [625] | patient study |
| 2011 | Newton | [626] | region of interest analyses |
| 2014 | Nichols | [627] | subjects in nicotine withdrawal |
| 2011 | Nixon | [628] | region of interest analyses |
| 2014 | Norbury | [629] | region of interest and connectivity analyses |
| 2009 | Nyberg | [630] | Miss, only (3-back - 1-back) and (1-back - baseline) |
| 2000 | Nystrom | [631] | Miss, between condition comparison |
| 2005 | Oakes | [632] | Miss, between algorithm comparison |
| 2011 | O'Daly | [633] | Miss, between condition comparison |
| 2014 | Oksanen | [634] | Miss, between condition comparison |
| 2011 | Ortiz-Gil | [635] | Miss, between group comparison |
| 2012 | Palacios | [636] | independent component analysis |
| 2011 | Papassotiropoulos | [637] | Miss, between condition comparison (imaging genetics) |
| 2011 | Park | [638] | auditory WM task |
| 2014 | Paulus | [639] | Miss, between condition comparison (imaging genetics) |
| 2008 | Pauly | [640] | Miss, between group comparison |
| 2010 | Pauly | [641] | Miss, between group comparison |
| 2014 | Pavisian | [642] | Miss, between group comparison |
| 2012 | Pavuluri | [643] | age younger than 18 |
| 2001 | Perlstein | [644] | Miss, between group comparison |
| 2003 | Perlstein | [645] | MS (main effect reported) |
| 2004 | Perlstein | [646] | MS (main effect reported) |
| 2001 | Pfefferbaum | [647] | age older than 60 |
| 2013 | Philip | [648] | region of interest analysis |
| 2012 | Plichta | [649] | region of interest analysis |
| 2014 | Plichta | [650] | region of interest analysis |
| 2002 | Pochon | [651] | region of interest analysis (masked by reward network) |
| 2008 | Pomarol-Clotet | [652] | Miss, between group comparison |
| 2012 | Pomarol-Clotet | [653] | incomplete report of coordinates |
| 2015 | Pomarol-Clotet | [654] | Miss, between group comparison |
| 2015 | Poudel | [655] | Miss, between group comparison |
| 2011 | Prilipko | [656] | Miss, between group comparison |
| 2012 | Prilipko | [657] | Miss, between group comparison |
| 2011 | Pu | [658] | functional near infrared spectroscopy |
| 2009 | Pyka | [659] | independent component analysis |
| 2013 | Pyka | [660] | resting state analysis |
| 2009 | Qin | [661] | MS (main effect reported) |
| 2001 | Rama | [662] | emotional variant of WM task |
| 2014 | Rampino | [663] | region of interest analysis |
| 2009 | Rasetti | [664] | Miss, between group comparison |
| 2013 | Richter | [665] | emotional variant of WM task |
| 2014 | Rodriguez-Cano | [666] | incomplete report of coordinates |
| 2009 | Rodriguez-Jimenez | [667] | auditory WM task |
| 2006 | Rose | [668] | incomplete report of coordinates |
| 2006 | Rose | [669] | Miss, between condition comparison |
| 2011 | Roussotte | [670] | age younger than 18 |
| 2009 | Royer | [671] | Miss, between group comparison |
| 2007 | Rudner | [672] | auditory WM task, sign language WM |
| 2013 | Rzucidlo | [673] | graph theoretical approach |
| 2003 | Sabri | [674] | PET study |
| 2014 | Sabri | [675] | combined working memory and dichotic-listening paradigm |
| 2012 | Sala-Llonch | [676] | independent component analysis and connectivity analysis |
| 2014 | Saliasi | [677] | independent component analysis |
| 2008 | Salvador | [678] | region of interest analysis, connectivity analysis |
| 2010 | Sambataro | [679] | independent component analysis and connectivity analysis |
| 2008 | Sanchez-Carrion | [680] | Miss, between group comparison |
| 2012 | Sandstrom | [681] | multi-variate analysis |
| 2014 | Sapara | [682] | Miss, between group comparison |
| 2012 | Satterthwaite | [683] | region of interest analysis |
| 2013 | Satterthwaite | [683] | age younger than 18 |
| 2012 | Savini | [684] | tactile WM task |
| 2003 | Scheibel | [685] | case study |
| 2010 | Schlagenhauf | [686] | MS (main effect reported) |
| 2003 | Schlosser | [687] | connectivity analysis |
| 2003 | Schlosser | [688] | connectivity analysis |
| 2007 | Schlosser | [689] | Miss, between group comparison |
| 2013 | Schmidt | [690] | connectivity analysis |
| 2014 | Schmidt | [691] | connectivity analysis |
| 2009 | Schoning | [692] | Miss, between group comparison |
| 2006 | Seidman | [693] | Miss, between group comparison |
| 2014 | Seidman | [694] | incomplete report of coordinates |
| 2012 | Smieskova | [695] | Miss, between group comparison |
| 2006 | Smith | [696] | incomplete report of coordinates |
| 2010 | Smith | [697] | Miss, between group comparison |
| 2009 | Smits | [698] | MS |
| 2009 | Spadoni | [699] | age younger than 18 |
| 2014 | Spreng | [700] | variant of face-matching WM task |
| 2012 | Stingl | [701] | Miss, between group comparison |
| 2011 | Stokes | [702] | Miss, between group comparison |
| 2010 | Stollstorff | [703] | age younger than 18 |
| 2010 | Stoodley | [704] | case study |
| 2014 | Subramaniam | [705] | Miss, between group comparison |
| 2012 | Sugranyes | [706] | Miss, between group comparison |
| 2010 | Sumowski | [707] | patient study |
| 2015 | Sundermann | [708] | Miss, between group comparison |
| 2007 | Surguladze | [709] | U |
| 2004 | Sweet | [710] | Miss, between group comparison |
| 2006 | Sweet | [711] | region of interest analyses |
| 2008 | Sweet | [712] | region of interest analyses |
| 2010 | Sweet | [713] | patient study |
| 2008 | Szatkowska | [714] | connectivity analysis |
| 2011 | Takeuchi | [715] | Miss, between condition comparison |
| 2011 | Takeuchi | [716] | correlation analysis |
| 2012 | Takeuchi | [717] | Miss, between condition comparison |
| 2014 | Takeuchi | [718] | correlation analysis |
| 2006 | Tan | [719] | Miss, between group comparison |
| 2011 | Tang | [720] | correlation analysis |
| 2015 | Teipel | [721] | Miss, between group comparison |
| 2005 | Thermenos | [722] | Miss, between group comparison |
| 2010 | Thermenos | [723] | Miss, between group comparison |
| 2011 | Thermenos | [724] | age younger than 18 |
| 2005 | Thomas | [725] | case study |
| 2006 | Thomas | [726] | subjects in sleep deprivation |
| 2011 | Thormodsen | [727] | Miss, between group comparison |
| 2013 | Thornton | [728] | emotional variant of WM task (social) |
| 2012 | Thurling | [729] | incomplete report of coordinates |
| 2007 | Tomasi | [730] | methodology of fMRI analysis |
| 2012 | Tu | [731] | connectivity analysis |
| 2005 | Valera | [732] | incomplete report of coordinates |
| 2010 | Valera | [733] | Miss, between group comparison |
| 2016 | van Ast | [734] | emotional variant of WM task (social) |
| 2007 | van der Wee | [735] | patient study |
| 2009 | Venneri | [736] | age older than 60 |
| 2012 | Vermeij | [737] | functional near infrared spectroscopy |
| 2012 | Voss | [738] | Miss, between condition comparison |
| 2013 | Vu | [739] | Miss, between group comparison |
| 2009 | Vuontela | [740] | age younger than 18 |
| 2007 | Walsh | [741] | Miss, between group comparison |
| 2003 | Walter | [742] | Miss, between group comparison |
| 2013 | Wardle | [743] | behavioral study |
| 2004 | Wei | [744] | auditory WM task |
| 2012 | Weiland | [745] | correlation analysis |
| 2013 | Winston | [746] | correlation analysis |
| 2004 | Wishart | [747] | Miss, between group comparison |
| 2006 | Wishart | [748] | age older than 60 |
| 2013 | Wolfe | [749] | age younger than 18 |
| 2014 | Wylie | [750] | methodology of fMRI analysis |
| 2005 | Xu | [751] | Miss, between condition comparison |
| 2006 | Xu | [752] | Miss, between condition comparison |
| 2011 | Yan | [753] | sample duplication (Yan, Brain and Cognition 2011) |
| 2013 | Yin | [754] | Miss, between group comparison |
| 2005 | Yoo | [755] | U |
| 2011 | Yu | [756] | age younger than 18 |
| 2014 | Zhang | [757] | age older than 60 |
| 2014 | Zhou | [758] | correlation analysis |
| 2006 | Zhu | [759] | region of interest analysis |
| 2007 | Ziemus | [760] | U |
| 2013 | Zou | [761] | correlation analysis |

# Part III

The results of the ER-WM conjunction analysis are highly concordant with the intersection of the ER and WM activation maps that have already been illustrated in **S1 Fig** of the formal manuscript. It is noteworthy that the activation foci of ER and WM are dissociable (except the midline dorsal prefrontal cortex) and that most of them are adjacent to each other (except the superior PFC). These adjacent activation foci are situated side by side (i.e., inferior PFC and insula) or up and down (i.e., in the middle PFC and posterior brain region). Because of the blurring process with Gaussian kernel, it is reasonable to expect that these adjacent foci overlap to a certain extent. Under the threshold of *P* < 0.05, there are 6 conjunction clusters, detailed below.

1. Anterior cingulate and pre-supplementary area (3672 mm^3, coordinates [-3.1 11.8 49.8]); this is the only highly overlapping cluster of ER and WM activation maps.

2. Left middle PFC (1072 mm^3, coordinates [-37.9 -2.3 48.3]); the activation masses for WM and ER in middle PFC were 15928 mm^3 and 3544 mm^3, respectively. This cluster was situated at the lower border of the corresponding ER activation mass and the upper border of the corresponding WM activation mass.

3. Right middle PFC (336 mm^3, coordinates [39.5 21.6 38.6]); this cluster was situated at the lower border of the corresponding ER activation mass and the upper border of the corresponding WM activation mass.

4. Right middle PFC (184 mm^3, coordinates [33.5 47.9 15.4]); this small cluster was situated near the frontal pole. In this particular region, the ER mass was nearly completely embedded in the WM mass. This locus is not a hot zone in ER and WM literature, and its significance is currently not clear.

5. Left inferior PFC (728 mm^3, coordinates [-35.1 18.9 2.7]); this cluster was situated at the interface between the inferior PFC and the anterior insula.

6. Right inferior PFC (648 mm^3, coordinates [35.8 18.4 0.6]); this cluster was situated at the interface between the inferior PFC and the anterior insula.

The above 6 conjunction foci are illustrated in the **S1 Fig**.

**S1 Fig. The results of ER-WM conjunction analysis**

**
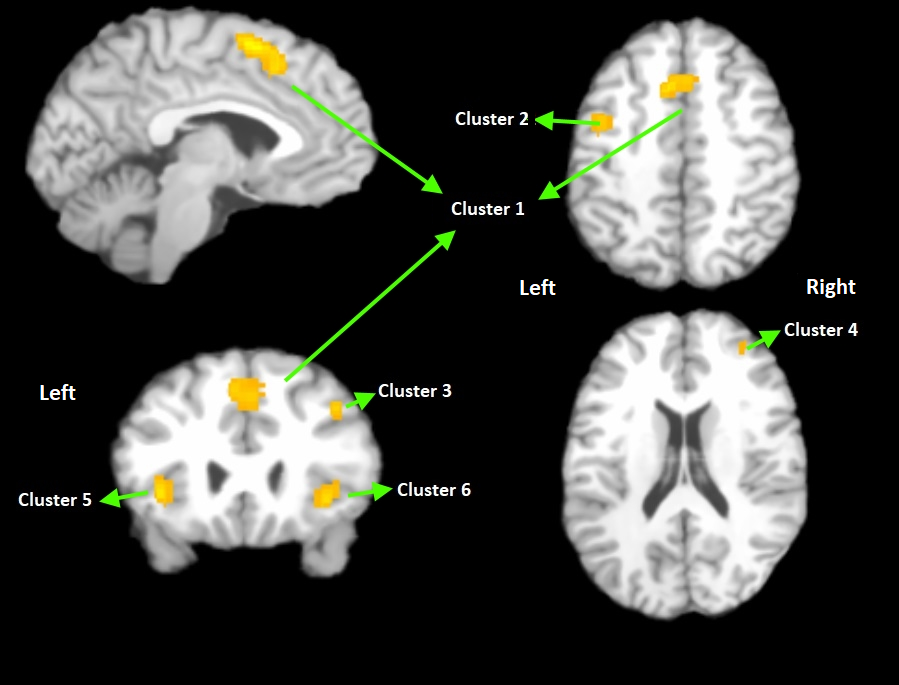
**

**References**

1. Abel KM, Allin MP, Kucharska-Pietura K, David A, Andrew C, Williams S, Brammer MJ, Phillips ML: **Ketamine alters neural processing of facial emotion recognition in healthy men: An fmri study**. *Neuroreport* 2003, **14**(3):387-391.

2. Abler B, Erk S, Herwig U, Walter H: **Anticipation of aversive stimuli activates extended amygdala in unipolar depression**. *J Psychiatr Res* 2007, **41**(6):511-522.

3. Abler B, Hofer C, Walter H, Erk S, Hoffmann H, Traue HC, Kessler H: **Habitual emotion regulation strategies and depressive symptoms in healthy subjects predict fmri brain activation patterns related to major depression**. *Psychiatry Res* 2010, **183**(2):105-113.

4. Achterberg M, van Duijvenvoorde AC, Bakermans-Kranenburg MJ, Crone EA: **Control your anger! The neural basis of aggression regulation in response to negative social feedback**. *Soc Cogn Affect Neurosci* 2016, **11**(5):712-720.

5. Akitsuki Y, Decety J: **Social context and perceived agency affects empathy for pain: An event-related fmri investigation**. *Neuroimage* 2009, **47**(2):722-734.

6. Albein-Urios N, Verdejo-Roman J, Asensio S, Soriano-Mas C, Martinez-Gonzalez JM, Verdejo-Garcia A: **Re-appraisal of negative emotions in cocaine dependence: Dysfunctional corticolimbic activation and connectivity**. *Addict Biol* 2014, **19**(3):415-426.

7. Alia-Klein N, Goldstein RZ, Tomasi D, Zhang L, Fagin-Jones S, Telang F, Wang GJ, Fowler JS, Volkow ND: **What is in a word? No versus yes differentially engage the lateral orbitofrontal cortex**. *Emotion* 2007, **7**(3):649-659.

8. Alkozei A, Smith R, Killgore WD: **Exposure to blue wavelength light modulates anterior cingulate cortex activation in response to 'uncertain' versus 'certain' anticipation of positive stimuli**. *Neurosci Lett* 2016, **616**:5-10.

9. Allard ES, Kensinger EA: **Age-related differences in functional connectivity during cognitive emotion regulation**. *J Gerontol B Psychol Sci Soc Sci* 2014, **69**(6):852-860.

10. Almeida JR, Kronhaus DM, Sibille EL, Langenecker SA, Versace A, Labarbara EJ, Phillips ML: **Abnormal left-sided orbitomedial prefrontal cortical-amygdala connectivity during happy and fear face processing: A potential neural mechanism of female mdd**. *Front Psychiatry* 2011, **2**:69.

11. Altmann U, Bohrn IC, Lubrich O, Menninghaus W, Jacobs AM: **Fact vs fiction--how paratextual information shapes our reading processes**. *Soc Cogn Affect Neurosci* 2014, **9**(1):22-29.

12. Amianto F, D'Agata F, Lavagnino L, Caroppo P, Abbate-Daga G, Righi D, Scarone S, Bergui M, Mortara P, Fassino S: **Intrinsic connectivity networks within cerebellum and beyond in eating disorders**. *Cerebellum* 2013, **12**(5):623-631.

13. Andari E, Richard N, Leboyer M, Sirigu A: **Adaptive coding of the value of social cues with oxytocin, an fmri study in autism spectrum disorder**. *Cortex* 2016, **76**:79-88.

14. Andreescu C, Gross JJ, Lenze E, Edelman KD, Snyder S, Tanase C, Aizenstein H: **Altered cerebral blood flow patterns associated with pathologic worry in the elderly**. *Depress Anxiety* 2011, **28**(3):202-209.

15. Anticevic A, Brumbaugh MS, Winkler AM, Lombardo LE, Barrett J, Corlett PR, Kober H, Gruber J, Repovs G, Cole MW *et al*: **Global prefrontal and fronto-amygdala dysconnectivity in bipolar i disorder with psychosis history**. *Biol Psychiatry* 2013, **73**(6):565-573.

16. Arizmendi B, Kaszniak AW, O'Connor MF: **Disrupted prefrontal activity during emotion processing in complicated grief: An fmri investigation**. *Neuroimage* 2016, **124**(Pt A):968-976.

17. Baczkowski BM, van Zutphen L, Siep N, Jacob GA, Domes G, Maier S, Sprenger A, Senft A, Willenborg B, Tuscher O *et al*: **Deficient amygdala-prefrontal intrinsic connectivity after effortful emotion regulation in borderline personality disorder**. *Eur Arch Psychiatry Clin Neurosci* 2016.

18. Ball TM, Ramsawh HJ, Campbell-Sills L, Paulus MP, Stein MB: **Prefrontal dysfunction during emotion regulation in generalized anxiety and panic disorders**. *Psychol Med* 2013, **43**(7):1475-1486.

19. Bangen KJ, Bergheim M, Kaup AR, Mirzakhanian H, Wierenga CE, Jeste DV, Eyler LT: **Brains of optimistic older adults respond less to fearful faces**. *J Neuropsychiatry Clin Neurosci* 2014, **26**(2):155-163.

20. Banks SJ, Eddy KT, Angstadt M, Nathan PJ, Phan KL: **Amygdala-frontal connectivity during emotion regulation**. *Soc Cogn Affect Neurosci* 2007, **2**(4):303-312.

21. Beauchamp KG, Kahn LE, Berkman ET: **Does inhibitory control training transfer?: Behavioral and neural effects on an untrained emotion regulation task**. *Soc Cogn Affect Neurosci* 2016, **11**(9):1374-1382.

22. Belden AC, Luby JL, Pagliaccio D, Barch DM: **Neural activation associated with the cognitive emotion regulation of sadness in healthy children**. *Dev Cogn Neurosci* 2014, **9**:136-147.

23. Belden AC, Pagliaccio D, Murphy ER, Luby JL, Barch DM: **Neural activation during cognitive emotion regulation in previously depressed compared to healthy children: Evidence of specific alterations**. *J Am Acad Child Adolesc Psychiatry* 2015, **54**(9):771-781.

24. Benelli E, Mergenthaler E, Walter S, Messina I, Sambin M, Buchheim A, Sim EJ, Viviani R: **Emotional and cognitive processing of narratives and individual appraisal styles: Recruitment of cognitive control networks vs. Modulation of deactivations**. *Front Hum Neurosci* 2012, **6**:239.

25. Bermingham R, Carballedo A, Lisiecka D, Fagan A, Morris D, Fahey C, Donohoe G, Meaney J, Gill M, Frodl T: **Effect of genetic variant in bicc1 on functional and structural brain changes in depression**. *Neuropsychopharmacology* 2012, **37**(13):2855-2862.

26. Berna C, Leknes S, Holmes EA, Edwards RR, Goodwin GM, Tracey I: **Induction of depressed mood disrupts emotion regulation neurocircuitry and enhances pain unpleasantness**. *Biol Psychiatry* 2010, **67**(11):1083-1090.

27. Bertocci MA, Bebko GM, Mullin BC, Langenecker SA, Ladouceur CD, Almeida JR, Phillips ML: **Abnormal anterior cingulate cortical activity during emotional n-back task performance distinguishes bipolar from unipolar depressed females**. *Psychol Med* 2012, **42**(7):1417-1428.

28. Birn RM, Patriat R, Phillips ML, Germain A, Herringa RJ: **Childhood maltreatment and combat posttraumatic stress differentially predict fear-related fronto-subcortical connectivity**. *Depress Anxiety* 2014, **31**(10):880-892.

29. Brady RO, Jr., Masters GA, Mathew IT, Margolis A, Cohen BM, Ongur D, Keshavan M: **State dependent cortico-amygdala circuit dysfunction in bipolar disorder**. *J Affect Disord* 2016, **201**:79-87.

30. Brassen S, Gamer M, Buchel C: **Anterior cingulate activation is related to a positivity bias and emotional stability in successful aging**. *Biol Psychiatry* 2010.

31. Brown MR, Benoit JR, Juhas M, Dametto E, Tse TT, MacKay M, Sen B, Carroll AM, Hodlevskyy O, Silverstone PH *et al*: **Fmri investigation of response inhibition, emotion, impulsivity, and clinical high-risk behavior in adolescents**. *Front Syst Neurosci* 2015, **9**:124.

32. Brown MR, Benoit JR, Juhas M, Lebel RM, MacKay M, Dametto E, Silverstone PH, Dolcos F, Dursun SM, Greenshaw AJ: **Neural correlates of high-risk behavior tendencies and impulsivity in an emotional go/nogo fmri task**. *Front Syst Neurosci* 2015, **9**:24.

33. Brown WJ, Wojtalik JA, Dewey D, Bruce SE, Yang Z, Sheline YI: **Affect and neural activity in women with ptsd during a task of emotional interference**. *J Affect Disord* 2016, **204**:9-15.

34. Bruce SE, Buchholz KR, Brown WJ, Yan L, Durbin A, Sheline YI: **Altered emotional interference processing in the amygdala and insula in women with post-traumatic stress disorder**. *Neuroimage Clin* 2012, **2**:43-49.

35. Bruhl AB, Herwig U, Delsignore A, Jancke L, Rufer M: **General emotion processing in social anxiety disorder: Neural issues of cognitive control**. *Psychiatry Res* 2013, **212**(2):108-115.

36. Bruhl AB, Scherpiet S, Sulzer J, Stampfli P, Seifritz E, Herwig U: **Real-time neurofeedback using functional mri could improve down-regulation of amygdala activity during emotional stimulation: A proof-of-concept study**. *Brain Topogr* 2014, **27**(1):138-148.

37. Buchheim A, Erk S, George C, Kachele H, Martius P, Pokorny D, Spitzer M, Walter H: **Neural response during the activation of the attachment system in patients with borderline personality disorder: An fmri study**. *Front Hum Neurosci* 2016, **10**:389.

38. Burklund LJ, Creswell JD, Irwin MR, Lieberman MD: **The common and distinct neural bases of affect labeling and reappraisal in healthy adults**. *Front Psychol* 2014, **5**:221.

39. Burklund LJ, Craske MG, Taylor SE, Lieberman MD: **Altered emotion regulation capacity in social phobia as a function of comorbidity**. *Soc Cogn Affect Neurosci* 2015, **10**(2):199-208.

40. Campbell-Sills L, Simmons AN, Lovero KL, Rochlin AA, Paulus MP, Stein MB: **Functioning of neural systems supporting emotion regulation in anxiety-prone individuals**. *Neuroimage* 2011, **54**(1):689-696.

41. Canli T, Congdon E, Gutknecht L, Constable RT, Lesch KP: **Amygdala responsiveness is modulated by tryptophan hydroxylase-2 gene variation**. *Journal of neural transmission (Vienna, Austria : 1996)* 2005, **112**(11):1479-1485.

42. Cao W, Luo C, Zhu B, Zhang D, Dong L, Gong J, Gong D, He H, Tu S, Yin W *et al*: **Resting-state functional connectivity in anterior cingulate cortex in normal aging**. *Front Aging Neurosci* 2014, **6**:280.

43. Carlson E, Saarikallio S, Toiviainen P, Bogert B, Kliuchko M, Brattico E: **Maladaptive and adaptive emotion regulation through music: A behavioral and neuroimaging study of males and females**. *Front Hum Neurosci* 2015, **9**:466.

44. Cascio CN, O'Donnell MB, Tinney FJ, Lieberman MD, Taylor SE, Strecher VJ, Falk EB: **Self-affirmation activates brain systems associated with self-related processing and reward and is reinforced by future orientation**. *Soc Cogn Affect Neurosci* 2016, **11**(4):621-629.

45. Caseras X, Murphy K, Lawrence NS, Fuentes-Claramonte P, Watts J, Jones DK, Phillips ML: **Emotion regulation deficits in euthymic bipolar i versus bipolar ii disorder: A functional and diffusion-tensor imaging study**. *Bipolar Disord* 2015, **17**(5):461-470.

46. Chase HW, Moses-Kolko EL, Zevallos C, Wisner KL, Phillips ML: **Disrupted posterior cingulate-amygdala connectivity in postpartum depressed women as measured with resting bold fmri**. *Soc Cogn Affect Neurosci* 2014, **9**(8):1069-1075.

47. Chen F, Lv X, Fang J, Yu S, Sui J, Fan L, Li T, Hong Y, Wang X, Wang W *et al*: **The effect of body-mind relaxation meditation induction on major depressive disorder: A resting-state fmri study**. *J Affect Disord* 2015, **183**:75-82.

48. Cheng Y, Lin CP, Liu HL, Hsu YY, Lim KE, Hung D, Decety J: **Expertise modulates the perception of pain in others**. *Curr Biol* 2007, **17**(19):1708-1713.

49. Cisler JM, James GA, Tripathi S, Mletzko T, Heim C, Hu XP, Mayberg HS, Nemeroff CB, Kilts CD: **Differential functional connectivity within an emotion regulation neural network among individuals resilient and susceptible to the depressogenic effects of early life stress**. *Psychol Med* 2013, **43**(3):507-518.

50. Cisler JM, Sigel BA, Steele JS, Smitherman S, Vanderzee K, Pemberton J, Kramer TL, Kilts CD: **Changes in functional connectivity of the amygdala during cognitive reappraisal predict symptom reduction during trauma-focused cognitive-behavioral therapy among adolescent girls with post-traumatic stress disorder**. *Psychol Med* 2016, **46**(14):3013-3023.

51. Clarke R, Johnstone T: **Prefrontal inhibition of threat processing reduces working memory interference**. *Front Hum Neurosci* 2013, **7**:228.

52. Clauss JA, Avery SN, VanDerKlok RM, Rogers BP, Cowan RL, Benningfield MM, Blackford JU: **Neurocircuitry underlying risk and resilience to social anxiety disorder**. *Depress Anxiety* 2014, **31**(10):822-833.

53. Cojan Y, Waber L, Carruzzo A, Vuilleumier P: **Motor inhibition in hysterical conversion paralysis**. *Neuroimage* 2009, **47**(3):1026-1037.

54. Coman IL, Gnirke MH, Middleton FA, Antshel KM, Fremont W, Higgins AM, Shprintzen RJ, Kates WR: **The effects of gender and catechol o-methyltransferase (comt) val108/158met polymorphism on emotion regulation in velo-cardio-facial syndrome (22q11.2 deletion syndrome): An fmri study**. *Neuroimage* 2010, **53**(3):1043-1050.

55. Compere L, Sperduti M, Gallarda T, Anssens A, Lion S, Delhommeau M, Martinelli P, Devauchelle AD, Oppenheim C, Piolino P: **Sex differences in the neural correlates of specific and general autobiographical memory**. *Front Hum Neurosci* 2016, **10**:285.

56. Costanzo ME, VanMeter JW, Janelle CM, Braun A, Miller MW, Oldham J, Russell BA, Hatfield BD: **Neural efficiency in expert cognitive-motor performers during affective challenge**. *Journal of motor behavior* 2016, **48**(6):573-588.

57. Cremers HR, Veer IM, Spinhoven P, Rombouts SA, Yarkoni T, Wager TD, Roelofs K: **Altered cortical-amygdala coupling in social anxiety disorder during the anticipation of giving a public speech**. *Psychol Med* 2015, **45**(7):1521-1529.

58. Das P, Calhoun V, Malhi GS: **Bipolar and borderline patients display differential patterns of functional connectivity among resting state networks**. *Neuroimage* 2014, **98**:73-81.

59. Davis TS, Mauss IB, Lumian D, Troy AS, Shallcross AJ, Zarolia P, Ford BQ, McRae K: **Emotional reactivity and emotion regulation among adults with a history of self-harm: Laboratory self-report and functional mri evidence**. *J Abnorm Psychol* 2014, **123**(3):499-509.

60. de Greck M, Shi Z, Wang G, Zuo X, Yang X, Wang X, Northoff G, Han S: **Culture modulates brain activity during empathy with anger**. *Neuroimage* 2012, **59**(3):2871-2882.

61. De Raedt R, Leyman L, Baeken C, Van Schuerbeek P, Luypaert R, Vanderhasselt MA, Dannlowski U: **Neurocognitive effects of hf-rtms over the dorsolateral prefrontal cortex on the attentional processing of emotional information in healthy women: An event-related fmri study**. *Biol Psychol* 2010, **85**(3):487-495.

62. Dean AC, Kohno M, Hellemann G, London ED: **Childhood maltreatment and amygdala connectivity in methamphetamine dependence: A pilot study**. *Brain Behav* 2014, **4**(6):867-876.

63. Delgado MR, Nearing KI, Ledoux JE, Phelps EA: **Neural circuitry underlying the regulation of conditioned fear and its relation to extinction**. *Neuron* 2008, **59**(5):829-838.

64. Demenescu LR, Renken R, Kortekaas R, van Tol MJ, Marsman JB, van Buchem MA, van der Wee NJ, Veltman DJ, den Boer JA, Aleman A: **Neural correlates of perception of emotional facial expressions in out-patients with mild-to-moderate depression and anxiety. A multicenter fmri study**. *Psychol Med* 2011, **41**(11):2253-2264.

65. Denkova E, Dolcos S, Dolcos F: **Neural correlates of 'distracting' from emotion during autobiographical recollection**. *Soc Cogn Affect Neurosci* 2015, **10**(2):219-230.

66. Denny BT, Ochsner KN, Weber J, Wager TD: **Anticipatory brain activity predicts the success or failure of subsequent emotion regulation**. *Soc Cogn Affect Neurosci* 2014, **9**(4):403-411.

67. Denny BT, Fan J, Liu X, Ochsner KN, Guerreri S, Mayson SJ, Rimsky L, McMaster A, New AS, Goodman M *et al*: **Elevated amygdala activity during reappraisal anticipation predicts anxiety in avoidant personality disorder**. *J Affect Disord* 2015, **172**:1-7.

68. Derntl B, Michel TM, Prempeh P, Backes V, Finkelmeyer A, Schneider F, Habel U: **Empathy in individuals clinically at risk for psychosis: Brain and behaviour**. *Br J Psychiatry* 2015, **207**(5):407-413.

69. Deveney CM, Connolly ME, Haring CT, Bones BL, Reynolds RC, Kim P, Pine DS, Leibenluft E: **Neural mechanisms of frustration in chronically irritable children**. *Am J Psychiatry* 2013, **170**(10):1186-1194.

70. DeWitt SJ, Aslan S, Filbey FM: **Adolescent risk-taking and resting state functional connectivity**. *Psychiatry Res* 2014, **222**(3):157-164.

71. Diers K, Weber F, Brocke B, Strobel A, Schonfeld S: **Instructions matter: A comparison of baseline conditions for cognitive emotion regulation paradigms**. *Front Psychol* 2014, **5**:347.

72. Dillon DG, Pizzagalli DA: **Evidence of successful modulation of brain activation and subjective experience during reappraisal of negative emotion in unmedicated depression**. *Psychiatry Res* 2013, **212**(2):99-107.

73. Dodhia S, Hosanagar A, Fitzgerald DA, Labuschagne I, Wood AG, Nathan PJ, Phan KL: **Modulation of resting-state amygdala-frontal functional connectivity by oxytocin in generalized social anxiety disorder**. *Neuropsychopharmacology* 2014, **39**(9):2061-2069.

74. Doerig N, Schlumpf Y, Spinelli S, Spati J, Brakowski J, Quednow BB, Seifritz E, Grosse Holtforth M: **Neural representation and clinically relevant moderators of individualised self-criticism in healthy subjects**. *Soc Cogn Affect Neurosci* 2014, **9**(9):1333-1340.

75. Dolcos S, Sung K, Denkova E, Dixon RA, Dolcos F: **Brain imaging investigation of the neural correlates of emotion regulation**. *J Vis Exp* 2011(54).

76. Dolcos S, Katsumi Y, Dixon RA: **The role of arousal in the spontaneous regulation of emotions in healthy aging: A fmri investigation**. *Front Psychol* 2014, **5**:681.

77. Doll A, Sorg C, Manoliu A, Woller A, Meng C, Forstl H, Zimmer C, Wohlschlager AM, Riedl V: **Shifted intrinsic connectivity of central executive and salience network in borderline personality disorder**. *Front Hum Neurosci* 2013, **7**:727.

78. Doll A, Holzel BK, Mulej Bratec S, Boucard CC, Xie X, Wohlschlager AM, Sorg C: **Mindful attention to breath regulates emotions via increased amygdala-prefrontal cortex connectivity**. *Neuroimage* 2016, **134**:305-313.

79. Dong M, Li J, Shi X, Gao S, Fu S, Liu Z, Liang F, Gong Q, Shi G, Tian J: **Altered baseline brain activity in experts measured by amplitude of low frequency fluctuations (alff): A resting state fmri study using expertise model of acupuncturists**. *Front Hum Neurosci* 2015, **9**:99.

80. Dorfel D, Lamke JP, Hummel F, Wagner U, Erk S, Walter H: **Common and differential neural networks of emotion regulation by detachment, reinterpretation, distraction, and expressive suppression: A comparative fmri investigation**. *Neuroimage* 2014, **101**:298-309.

81. Dougherty LR, Blankenship SL, Spechler PA, Padmala S, Pessoa L: **An fmri pilot study of cognitive reappraisal in children: Divergent effects on brain and behavior**. *J Psychopathol Behav Assess* 2015, **37**(4):634-644.

82. Downar J, Geraci J, Salomons TV, Dunlop K, Wheeler S, McAndrews MP, Bakker N, Blumberger DM, Daskalakis ZJ, Kennedy SH *et al*: **Anhedonia and reward-circuit connectivity distinguish nonresponders from responders to dorsomedial prefrontal repetitive transcranial magnetic stimulation in major depression**. *Biol Psychiatry* 2014, **76**(3):176-185.

83. Drabant EM, McRae K, Manuck SB, Hariri AR, Gross JJ: **Individual differences in typical reappraisal use predict amygdala and prefrontal responses**. *Biol Psychiatry* 2009, **65**(5):367-373.

84. Dziobek I, Preissler S, Grozdanovic Z, Heuser I, Heekeren HR, Roepke S: **Neuronal correlates of altered empathy and social cognition in borderline personality disorder**. *Neuroimage* 2011, **57**(2):539-548.

85. Eack SM, Newhill CE, Keshavan MS: **Cognitive enhancement therapy improves resting-state functional connectivity in early course schizophrenia**. *Journal of the Society for Social Work and Research* 2016, **7**(2):211-230.

86. Eack SM, Wojtalik JA, Barb SM, Newhill CE, Keshavan MS, Phillips ML: **Fronto-limbic brain dysfunction during the regulation of emotion in schizophrenia**. *PLoS ONE* 2016, **11**(3):e0149297.

87. Emmerling F, Schuhmann T, Lobbestael J, Arntz A, Brugman S, Sack AT: **The role of the insular cortex in retaliation**. *PLoS ONE* 2016, **11**(4):e0152000.

88. Engen HG, Singer T: **Compassion-based emotion regulation up-regulates experienced positive affect and associated neural networks**. *Soc Cogn Affect Neurosci* 2015, **10**(9):1291-1301.

89. Enzi B, Lissek S, Edel MA, Tegenthoff M, Nicolas V, Scherbaum N, Juckel G, Roser P: **Alterations of monetary reward and punishment processing in chronic cannabis users: An fmri study**. *PLoS ONE* 2015, **10**(3):e0119150.

90. Enzi B, Amirie S, Brune M: **Empathy for pain-related dorsolateral prefrontal activity is modulated by angry face perception**. *Exp Brain Res* 2016, **234**(11):3335-3345.

91. Erk S, Abler B, Walter H: **Cognitive modulation of emotion anticipation**. *Eur J Neurosci* 2006, **24**(4):1227-1236.

92. Erk S, von Kalckreuth A, Walter H: **Neural long-term effects of emotion regulation on episodic memory processes**. *Neuropsychologia* 2010, **48**(4):989-996.

93. Ertl M, Hildebrandt M, Ourina K, Leicht G, Mulert C: **Emotion regulation by cognitive reappraisal - the role of frontal theta oscillations**. *Neuroimage* 2013, **81**:412-421.

94. Etkin A, Prater KE, Hoeft F, Menon V, Schatzberg AF: **Failure of anterior cingulate activation and connectivity with the amygdala during implicit regulation of emotional processing in generalized anxiety disorder**. *Am J Psychiatry* 2010, **167**(5):545-554.

95. Farb NA, Anderson AK, Bloch RT, Segal ZV: **Mood-linked responses in medial prefrontal cortex predict relapse in patients with recurrent unipolar depression**. *Biol Psychiatry* 2011, **70**(4):366-372.

96. Favre P, Polosan M, Pichat C, Bougerol T, Baciu M: **Cerebral correlates of abnormal emotion conflict processing in euthymic bipolar patients: A functional mri study**. *PLoS ONE* 2015, **10**(8):e0134961.

97. Felder JN, Smoski MJ, Kozink RV, Froeliger B, McClernon J, Bizzell J, Petty C, Dichter GS: **Neural mechanisms of subclinical depressive symptoms in women: A pilot functional brain imaging study**. *BMC Psychiatry* 2012, **12**:152.

98. Feng C, DeMarco AC, Haroon E, Rilling JK: **Neuroticism modulates the effects of intranasal vasopressin treatment on the neural response to positive and negative social interactions**. *Neuropsychologia* 2015, **73**:108-115.

99. Firk C, Siep N, Markus CR: **Serotonin transporter genotype modulates cognitive reappraisal of negative emotions: A functional magnetic resonance imaging study**. *Soc Cogn Affect Neurosci* 2013, **8**(3):247-258.

100. Fisher HE, Brown LL, Aron A, Strong G, Mashek D: **Reward, addiction, and emotion regulation systems associated with rejection in love**. *J Neurophysiol* 2010, **104**(1):51-60.

101. Fox GR, Sobhani M, Aziz-Zadeh L: **Witnessing hateful people in pain modulates brain activity in regions associated with physical pain and reward**. *Front Psychol* 2013, **4**:772.

102. Frijling JL, van Zuiden M, Koch SB, Nawijn L, Veltman DJ, Olff M: **Intranasal oxytocin affects amygdala functional connectivity after trauma script-driven imagery in distressed recently trauma-exposed individuals**. *Neuropsychopharmacology* 2016, **41**(5):1286-1296.

103. Fulwiler CE, King JA, Zhang N: **Amygdala-orbitofrontal resting-state functional connectivity is associated with trait anger**. *Neuroreport* 2012, **23**(10):606-610.

104. Gaebler M, Daniels JK, Lamke JP, Fydrich T, Walter H: **Behavioural and neural correlates of self-focused emotion regulation in social anxiety disorder**. *J Psychiatry Neurosci* 2014, **39**(4):249-258.

105. Gaffrey MS, Barch DM, Singer J, Shenoy R, Luby JL: **Disrupted amygdala reactivity in depressed 4- to 6-year-old children**. *J Am Acad Child Adolesc Psychiatry* 2013, **52**(7):737-746.

106. Gardener EK, Carr AR, Macgregor A, Felmingham KL: **Sex differences and emotion regulation: An event-related potential study**. *PLoS ONE* 2013, **8**(10):e73475.

107. Gasnier M, Pelissolo A, Bondolfi G, Pelissolo S, Tomba M, Mallet L, N'Diaye K: **[mindfulness-based interventions in obsessive-compulsive disorder: Mechanisms of action and presentation of a pilot study]**. *Encephale* 2016.

108. Gee DG, Karlsgodt KH, van Erp TG, Bearden CE, Lieberman MD, Belger A, Perkins DO, Olvet DM, Cornblatt BA, Constable T *et al*: **Altered age-related trajectories of amygdala-prefrontal circuitry in adolescents at clinical high risk for psychosis: A preliminary study**. *Schizophr Res* 2012, **134**(1):1-9.

109. Gee DG, Gabard-Durnam LJ, Flannery J, Goff B, Humphreys KL, Telzer EH, Hare TA, Bookheimer SY, Tottenham N: **Early developmental emergence of human amygdala-prefrontal connectivity after maternal deprivation**. *Proc Natl Acad Sci U S A* 2013, **110**(39):15638-15643.

110. Gee DG, Gabard-Durnam L, Telzer EH, Humphreys KL, Goff B, Shapiro M, Flannery J, Lumian DS, Fareri DS, Caldera C *et al*: **Maternal buffering of human amygdala-prefrontal circuitry during childhood but not during adolescence**. *Psychol Sci* 2014, **25**(11):2067-2078.

111. George MS, Anton RF, Bloomer C, Teneback C, Drobes DJ, Lorberbaum JP, Nahas Z, Vincent DJ: **Activation of prefrontal cortex and anterior thalamus in alcoholic subjects on exposure to alcohol-specific cues**. *Arch Gen Psychiatry* 2001, **58**(4):345-352.

112. Gilam G, Lin T, Raz G, Azrielant S, Fruchter E, Ariely D, Hendler T: **Neural substrates underlying the tendency to accept anger-infused ultimatum offers during dynamic social interactions**. *Neuroimage* 2015, **120**:400-411.

113. Gillath O, Bunge SA, Shaver PR, Wendelken C, Mikulincer M: **Attachment-style differences in the ability to suppress negative thoughts: Exploring the neural correlates**. *Neuroimage* 2005, **28**(4):835-847.

114. Giuliani NR, Pfeifer JH: **Age-related changes in reappraisal of appetitive cravings during adolescence**. *Neuroimage* 2015, **108**:173-181.

115. Glaser YG, Zubieta JK, Hsu DT, Villafuerte S, Mickey BJ, Trucco EM, Burmeister M, Zucker RA, Heitzeg MM: **Indirect effect of corticotropin-releasing hormone receptor 1 gene variation on negative emotionality and alcohol use via right ventrolateral prefrontal cortex**. *J Neurosci* 2014, **34**(11):4099-4107.

116. Glotzbach E, Muhlberger A, Gschwendtner K, Fallgatter AJ, Pauli P, Herrmann MJ: **Prefrontal brain activation during emotional processing: A functional near infrared spectroscopy study (fnirs)**. *Open Neuroimag J* 2011, **5**:33-39.

117. Goerlich-Dobre KS, Witteman J, Schiller NO, van Heuven VJ, Aleman A, Martens S: **Blunted feelings: Alexithymia is associated with a diminished neural response to speech prosody**. *Soc Cogn Affect Neurosci* 2014, **9**(8):1108-1117.

118. Goldin PR, Manber T, Hakimi S, Canli T, Gross JJ: **Neural bases of social anxiety disorder: Emotional reactivity and cognitive regulation during social and physical threat**. *Arch Gen Psychiatry* 2009, **66**(2):170-180.

119. Goldin PR, Manber-Ball T, Werner K, Heimberg R, Gross JJ: **Neural mechanisms of cognitive reappraisal of negative self-beliefs in social anxiety disorder**. *Biol Psychiatry* 2009, **66**(12):1091-1099.

120. Goldin PR, Gross JJ: **Effects of mindfulness-based stress reduction (mbsr) on emotion regulation in social anxiety disorder**. *Emotion* 2010, **10**(1):83-91.

121. Goldin P, Ziv M, Jazaieri H, Hahn K, Gross JJ: **Mbsr vs aerobic exercise in social anxiety: Fmri of emotion regulation of negative self-beliefs**. *Soc Cogn Affect Neurosci* 2013, **8**(1):65-72.

122. Goldin PR, Ziv M, Jazaieri H, Hahn K, Heimberg R, Gross JJ: **Impact of cognitive behavioral therapy for social anxiety disorder on the neural dynamics of cognitive reappraisal of negative self-beliefs: Randomized clinical trial**. *JAMA Psychiatry* 2013, **70**(10):1048-1056.

123. Goldin PR, Ziv M, Jazaieri H, Weeks J, Heimberg RG, Gross JJ: **Impact of cognitive-behavioral therapy for social anxiety disorder on the neural bases of emotional reactivity to and regulation of social evaluation**. *Behav Res Ther* 2014, **62**:97-106.

124. Goldstein RZ, Alia-Klein N, Tomasi D, Carrillo JH, Maloney T, Woicik PA, Wang R, Telang F, Volkow ND: **Anterior cingulate cortex hypoactivations to an emotionally salient task in cocaine addiction**. *Proc Natl Acad Sci U S A* 2009, **106**(23):9453-9458.

125. Gollan JK, Connolly M, Buchanan A, Hoxha D, Rosebrock L, Cacioppo J, Csernansky J, Wang X: **Neural substrates of negativity bias in women with and without major depression**. *Biol Psychol* 2015, **109**:184-191.

126. Goodman M, Carpenter D, Tang CY, Goldstein KE, Avedon J, Fernandez N, Mascitelli KA, Blair NJ, New AS, Triebwasser J *et al*: **Dialectical behavior therapy alters emotion regulation and amygdala activity in patients with borderline personality disorder**. *J Psychiatr Res* 2014, **57**:108-116.

127. Goodman AM, Wheelock MD, Harnett NG, Mrug S, Granger DA, Knight DC: **The hippocampal response to psychosocial stress varies with salivary uric acid level**. *Neuroscience* 2016, **339**:396-401.

128. Gorka SM, Phan KL, Lyons M, Mori S, Angstadt M, Rabinak CA: **Cannabinoid modulation of frontolimbic activation and connectivity during volitional regulation of negative affect**. *Neuropsychopharmacology* 2016, **41**(7):1888-1896.

129. Grant JA, Courtemanche J, Rainville P: **A non-elaborative mental stance and decoupling of executive and pain-related cortices predicts low pain sensitivity in zen meditators**. *Pain* 2011, **152**(1):150-156.

130. Grecucci A, Giorgetta C, Van't Wout M, Bonini N, Sanfey AG: **Reappraising the ultimatum: An fmri study of emotion regulation and decision making**. *Cereb Cortex* 2013, **23**(2):399-410.

131. Grecucci A, Giorgetta C, Bonini N, Sanfey AG: **Reappraising social emotions: The role of inferior frontal gyrus, temporo-parietal junction and insula in interpersonal emotion regulation**. *Front Hum Neurosci* 2013, **7**:523.

132. Greening SG, Osuch EA, Williamson PC, Mitchell DG: **The neural correlates of regulating positive and negative emotions in medication-free major depression**. *Soc Cogn Affect Neurosci* 2014, **9**(5):628-637.

133. Gu X, Kirk U, Lohrenz TM, Montague PR: **Cognitive strategies regulate fictive, but not reward prediction error signals in a sequential investment task**. *Hum Brain Mapp* 2014, **35**(8):3738-3749.

134. Guhn A, Domschke K, Muller LD, Dresler T, Eff F, Kopf J, Deckert J, Reif A, Herrmann MJ: **Neuropeptide s receptor gene variation and neural correlates of cognitive emotion regulation**. *Soc Cogn Affect Neurosci* 2015, **10**(12):1730-1737.

135. Gyurak A, Hooker CI, Miyakawa A, Verosky S, Luerssen A, Ayduk ON: **Individual differences in neural responses to social rejection: The joint effect of self-esteem and attentional control**. *Soc Cogn Affect Neurosci* 2012, **7**(3):322-331.

136. Habel U, Pauly K, Koch K, Kellermann T, Reske M, Backes V, Stocker T, Amunts K, Shah NJ, Schneider F: **Emotion-cognition interactions in schizophrenia**. *World J Biol Psychiatry* 2010, **11**(8):934-944.

137. Hafeman DM, Bebko G, Bertocci MA, Fournier JC, Bonar L, Perlman SB, Travis M, Gill MK, Diwadkar VA, Sunshine JL *et al*: **Abnormal deactivation of the inferior frontal gyrus during implicit emotion processing in youth with bipolar disorder: Attenuated by medication**. *J Psychiatr Res* 2014, **58**:129-136.

138. Hallam GP, Webb TL, Sheeran P, Miles E, Niven K, Wilkinson ID, Hunter MD, Woodruff PW, Totterdell P, Farrow TF: **The neural correlates of regulating another person's emotions: An exploratory fmri study**. *Front Hum Neurosci* 2014, **8**:376.

139. Hamm LL, Jacobs RH, Johnson MW, Fitzgerald DA, Fitzgerald KD, Langenecker SA, Monk CS, Phan KL: **Aberrant amygdala functional connectivity at rest in pediatric anxiety disorders**. *Biol Mood Anxiety Disord* 2014, **4**(1):15.

140. Han S, Qin J, Ma Y: **Neurocognitive processes of linguistic cues related to death**. *Neuropsychologia* 2010, **48**(12):3436-3442.

141. Han HJ, Jung WH, Yun JY, Park JW, Cho KK, Hur JW, Shin NY, Lee TY, Kwon JS: **Disruption of effective connectivity from the dorsolateral prefrontal cortex to the orbitofrontal cortex by negative emotional distraction in obsessive-compulsive disorder**. *Psychol Med* 2016, **46**(5):921-932.

142. Hare TA, Tottenham N, Davidson MC, Glover GH, Casey BJ: **Contributions of amygdala and striatal activity in emotion regulation**. *Biol Psychiatry* 2005, **57**(6):624-632.

143. Hare TA, Tottenham N, Galvan A, Voss HU, Glover GH, Casey BJ: **Biological substrates of emotional reactivity and regulation in adolescence during an emotional go-nogo task**. *Biol Psychiatry* 2008, **63**(10):927-934.

144. Hariri AR, Mattay VS, Tessitore A, Fera F, Weinberger DR: **Neocortical modulation of the amygdala response to fearful stimuli**. *Biol Psychiatry* 2003, **53**(6):494-501.

145. Heissler J, Kanske P, Schonfelder S, Wessa M: **Inefficiency of emotion regulation as vulnerability marker for bipolar disorder: Evidence from healthy individuals with hypomanic personality**. *J Affect Disord* 2014, **152-154**:83-90.

146. Heller AS, Johnstone T, Light SN, Peterson MJ, Kolden GG, Kalin NH, Davidson RJ: **Relationships between changes in sustained fronto-striatal connectivity and positive affect in major depression resulting from antidepressant treatment**. *Am J Psychiatry* 2013, **170**(2):197-206.

147. Heller AS, Johnstone T, Peterson MJ, Kolden GG, Kalin NH, Davidson RJ: **Increased prefrontal cortex activity during negative emotion regulation as a predictor of depression symptom severity trajectory over 6 months**. *JAMA Psychiatry* 2013, **70**(11):1181-1189.

148. Helmbold K, Zvyagintsev M, Dahmen B, Bubenzer-Busch S, Gaber TJ, Crockett MJ, Klasen M, Sanchez CL, Eisert A, Konrad K *et al*: **Effects of serotonin depletion on punishment processing in the orbitofrontal and anterior cingulate cortices of healthy women**. *Eur Neuropsychopharmacol* 2015, **25**(6):846-856.

149. Hennig-Fast K, Michl P, Muller J, Niedermeier N, Coates U, Muller N, Engel RR, Moller HJ, Reiser M, Meindl T: **Obsessive-compulsive disorder--a question of conscience? An fmri study of behavioural and neurofunctional correlates of shame and guilt**. *J Psychiatr Res* 2015, **68**:354-362.

150. Hermann A, Leutgeb V, Scharmuller W, Vaitl D, Schienle A, Stark R: **Individual differences in cognitive reappraisal usage modulate the time course of brain activation during symptom provocation in specific phobia**. *Biol Mood Anxiety Disord* 2013, **3**(1):16.

151. Hermann A, Keck T, Stark R: **Dispositional cognitive reappraisal modulates the neural correlates of fear acquisition and extinction**. *Neurobiol Learn Mem* 2014, **113**:115-124.

152. Hermann A, Kress L, Stark R: **Neural correlates of immediate and prolonged effects of cognitive reappraisal and distraction on emotional experience**. *Brain Imaging Behav* 2016.

153. Herremans SC, De Raedt R, Van Schuerbeek P, Marinazzo D, Matthys F, De Mey J, Baeken C: **Accelerated hf-rtms protocol has a rate-dependent effect on dacc activation in alcohol-dependent patients: An open-label feasibility study**. *Alcohol Clin Exp Res* 2016, **40**(1):196-205.

154. Herwig U, Baumgartner T, Kaffenberger T, Bruhl A, Kottlow M, Schreiter-Gasser U, Abler B, Jancke L, Rufer M: **Modulation of anticipatory emotion and perception processing by cognitive control**. *Neuroimage* 2007, **37**(2):652-662.

155. Herwig U, Kaffenberger T, Jancke L, Bruhl AB: **Self-related awareness and emotion regulation**. *Neuroimage* 2010, **50**(2):734-741.

156. Herwig U, Dhum M, Hittmeyer A, Opialla S, Scherpiet S, Keller C, Bruhl AB, Siegrist M: **Neural signaling of food healthiness associated with emotion processing**. *Front Aging Neurosci* 2016, **8**:16.

157. Holland AC, Kensinger EA: **The neural correlates of cognitive reappraisal during emotional autobiographical memory recall**. *J Cogn Neurosci* 2013, **25**(1):87-108.

158. Holland AC, Kensinger EA: **An fmri investigation of the cognitive reappraisal of negative memories**. *Neuropsychologia* 2013, **51**(12):2389-2400.

159. Holtmann J, Herbort MC, Wustenberg T, Soch J, Richter S, Walter H, Roepke S, Schott BH: **Trait anxiety modulates fronto-limbic processing of emotional interference in borderline personality disorder**. *Front Hum Neurosci* 2013, **7**:54.

160. Holzel BK, Hoge EA, Greve DN, Gard T, Creswell JD, Brown KW, Barrett LF, Schwartz C, Vaitl D, Lazar SW: **Neural mechanisms of symptom improvements in generalized anxiety disorder following mindfulness training**. *Neuroimage Clin* 2013, **2**:448-458.

161. Hooker CI, Gyurak A, Verosky SC, Miyakawa A, Ayduk O: **Neural activity to a partner's facial expression predicts self-regulation after conflict**. *Biol Psychiatry* 2010, **67**(5):406-413.

162. Hulvershorn LA, Finn P, Hummer TA, Leibenluft E, Ball B, Gichina V, Anand A: **Cortical activation deficits during facial emotion processing in youth at high risk for the development of substance use disorders**. *Drug Alcohol Depend* 2013, **131**(3):230-237.

163. Hulvershorn LA, Mennes M, Castellanos FX, Di Martino A, Milham MP, Hummer TA, Roy AK: **Abnormal amygdala functional connectivity associated with emotional lability in children with attention-deficit/hyperactivity disorder**. *J Am Acad Child Adolesc Psychiatry* 2014, **53**(3):351-361.e351.

164. Hwang S, White SF, Nolan ZT, Sinclair S, Blair RJ: **Neurodevelopmental changes in the responsiveness of systems involved in top down attention and emotional responding**. *Neuropsychologia* 2014, **62**:277-285.

165. Hwang S, White SF, Nolan ZT, Craig Williams W, Sinclair S, Blair RJ: **Executive attention control and emotional responding in attention-deficit/hyperactivity disorder--a functional mri study**. *Neuroimage Clin* 2015, **9**:545-554.

166. Hwang JW, Xin SC, Ou YM, Zhang WY, Liang YL, Chen J, Yang XQ, Chen XY, Guo TW, Yang XJ *et al*: **Enhanced default mode network connectivity with ventral striatum in subthreshold depression individuals**. *J Psychiatr Res* 2016, **76**:111-120.

167. Ichikawa N, Siegle GJ, Jones NP, Kamishima K, Thompson WK, Gross JJ, Ohira H: **Feeling bad about screwing up: Emotion regulation and action monitoring in the anterior cingulate cortex**. *Cogn Affect Behav Neurosci* 2011, **11**(3):354-371.

168. Ives-Deliperi VL, Howells F, Stein DJ, Meintjes EM, Horn N: **The effects of mindfulness-based cognitive therapy in patients with bipolar disorder: A controlled functional mri investigation**. *J Affect Disord* 2013, **150**(3):1152-1157.

169. Jacob GA, Zvonik K, Kamphausen S, Sebastian A, Maier S, Philipsen A, Tebartz van Elst L, Lieb K, Tuscher O: **Emotional modulation of motor response inhibition in women with borderline personality disorder: An fmri study**. *J Psychiatry Neurosci* 2013, **38**(3):164-172.

170. Jarcho JM, Berkman ET, Lieberman MD: **The neural basis of rationalization: Cognitive dissonance reduction during decision-making**. *Soc Cogn Affect Neurosci* 2011, **6**(4):460-467.

171. Jarcho JM, Tanofsky-Kraff M, Nelson EE, Engel SG, Vannucci A, Field SE, Romer AL, Hannallah L, Brady SM, Demidowich AP *et al*: **Neural activation during anticipated peer evaluation and laboratory meal intake in overweight girls with and without loss of control eating**. *Neuroimage* 2015, **108**:343-353.

172. Javanbakht A, Kim P, Swain JE, Evans GW, Phan KL, Liberzon I: **Sex-specific effects of childhood poverty on neurocircuitry of processing of emotional cues: A neuroimaging study**. *Behavioral sciences (Basel, Switzerland)* 2016, **6**(4).

173. Jeong JW, Diwadkar VA, Chugani CD, Sinsoongsud P, Muzik O, Behen ME, Chugani HT, Chugani DC: **Congruence of happy and sad emotion in music and faces modifies cortical audiovisual activation**. *Neuroimage* 2011, **54**(4):2973-2982.

174. Johnson SM, Burgess Moser M, Beckes L, Smith A, Dalgleish T, Halchuk R, Hasselmo K, Greenman PS, Merali Z, Coan JA: **Soothing the threatened brain: Leveraging contact comfort with emotionally focused therapy**. *PLoS ONE* 2013, **8**(11):e79314.

175. Johnstone T, van Reekum CM, Urry HL, Kalin NH, Davidson RJ: **Failure to regulate: Counterproductive recruitment of top-down prefrontal-subcortical circuitry in major depression**. *J Neurosci* 2007, **27**(33):8877-8884.

176. Kadosh KC, Luo Q, de Burca C, Sokunbi MO, Feng J, Linden DE, Lau JY: **Using real-time fmri to influence effective connectivity in the developing emotion regulation network**. *Neuroimage* 2016, **125**:616-626.

177. Kalisch R, Wiech K, Herrmann K, Dolan RJ: **Neural correlates of self-distraction from anxiety and a process model of cognitive emotion regulation**. *J Cogn Neurosci* 2006, **18**(8):1266-1276.

178. Kanske P, Heissler J, Schonfelder S, Bongers A, Wessa M: **How to regulate emotion? Neural networks for reappraisal and distraction**. *Cereb Cortex* 2011, **21**(6):1379-1388.

179. Kanske P, Heissler J, Schonfelder S, Wessa M: **Neural correlates of emotion regulation deficits in remitted depression: The influence of regulation strategy, habitual regulation use, and emotional valence**. *Neuroimage* 2012, **61**(3):686-693.

180. Kanske P, Schonfelder S, Forneck J, Wessa M: **Impaired regulation of emotion: Neural correlates of reappraisal and distraction in bipolar disorder and unaffected relatives**. *Transl Psychiatry* 2015, **5**:e497.

181. Kerr KL, Avery JA, Barcalow JC, Moseman SE, Bodurka J, Bellgowan PS, Simmons WK: **Trait impulsivity is related to ventral acc and amygdala activity during primary reward anticipation**. *Soc Cogn Affect Neurosci* 2015, **10**(1):36-42.

182. Keynan JN, Meir-Hasson Y, Gilam G, Cohen A, Jackont G, Kinreich S, Ikar L, Or-Borichev A, Etkin A, Gyurak A *et al*: **Limbic activity modulation guided by functional magnetic resonance imaging-inspired electroencephalography improves implicit emotion regulation**. *Biol Psychiatry* 2016.

183. Kienast T, Schlagenhauf F, Rapp MA, Wrase J, Daig I, Buchholz HG, Smolka MN, Grunder G, Kumakura Y, Cumming P *et al*: **Dopamine-modulated aversive emotion processing fails in alcohol-dependent patients**. *Pharmacopsychiatry* 2013, **46**(4):130-136.

184. Kim P, Evans GW, Angstadt M, Ho SS, Sripada CS, Swain JE, Liberzon I, Phan KL: **Effects of childhood poverty and chronic stress on emotion regulatory brain function in adulthood**. *Proc Natl Acad Sci U S A* 2013, **110**(46):18442-18447.

185. Kim EJ, Kyeong S, Cho SW, Chun JW, Park HJ, Kim J, Kim J, Dolan RJ, Kim JJ: **Happier people show greater neural connectivity during negative self-referential processing**. *PLoS ONE* 2016, **11**(2):e0149554.

186. Klucken T, Kruse O, Schweckendiek J, Stark R: **Increased skin conductance responses and neural activity during fear conditioning are associated with a repressive coping style**. *Front Behav Neurosci* 2015, **9**:132.

187. Klucken T, Kruse O, Wehrum-Osinsky S, Hennig J, Schweckendiek J, Stark R: **Impact of comt val158met-polymorphism on appetitive conditioning and amygdala/prefrontal effective connectivity**. *Hum Brain Mapp* 2015, **36**(3):1093-1101.

188. Klucken T, Wehrum-Osinsky S, Schweckendiek J, Kruse O, Stark R: **Altered appetitive conditioning and neural connectivity in subjects with compulsive sexual behavior**. *J Sex Med* 2016, **13**(4):627-636.

189. Klumpp H, Angstadt M, Phan KL: **Insula reactivity and connectivity to anterior cingulate cortex when processing threat in generalized social anxiety disorder**. *Biol Psychol* 2012, **89**(1):273-276.

190. Klumpp H, Keutmann MK, Fitzgerald DA, Shankman SA, Phan KL: **Resting state amygdala-prefrontal connectivity predicts symptom change after cognitive behavioral therapy in generalized social anxiety disorder**. *Biol Mood Anxiety Disord* 2014, **4**(1):14.

191. Kogler L, Gur RC, Derntl B: **Sex differences in cognitive regulation of psychosocial achievement stress: Brain and behavior**. *Hum Brain Mapp* 2015, **36**(3):1028-1042.

192. Kohn N, Falkenberg I, Kellermann T, Eickhoff SB, Gur RC, Habel U: **Neural correlates of effective and ineffective mood induction**. *Soc Cogn Affect Neurosci* 2014, **9**(6):864-872.

193. Kraus A, Valerius G, Seifritz E, Ruf M, Bremner JD, Bohus M, Schmahl C: **Script-driven imagery of self-injurious behavior in patients with borderline personality disorder: A pilot fmri study**. *Acta Psychiatr Scand* 2010, **121**(1):41-51.

194. Krendl AC, Heatherton TF, Kensinger EA: **Aging minds and twisting attitudes: An fmri investigation of age differences in inhibiting prejudice**. *Psychol Aging* 2009, **24**(3):530-541.

195. Krendl AC, Kensinger EA, Ambady N: **How does the brain regulate negative bias to stigma?** *Soc Cogn Affect Neurosci* 2012, **7**(6):715-726.

196. Kret ME, Denollet J, Grezes J, de Gelder B: **The role of negative affectivity and social inhibition in perceiving social threat: An fmri study**. *Neuropsychologia* 2011, **49**(5):1187-1193.

197. Kross E, Davidson M, Weber J, Ochsner K: **Coping with emotions past: The neural bases of regulating affect associated with negative autobiographical memories**. *Biol Psychiatry* 2009, **65**(5):361-366.

198. Kumari V, Das M, Taylor PJ, Barkataki I, Andrew C, Sumich A, Williams SC, Ffytche DH: **Neural and behavioural responses to threat in men with a history of serious violence and schizophrenia or antisocial personality disorder**. *Schizophr Res* 2009, **110**(1-3):47-58.

199. Ladouceur CD, Farchione T, Diwadkar V, Pruitt P, Radwan J, Axelson DA, Birmaher B, Phillips ML: **Differential patterns of abnormal activity and connectivity in the amygdala-prefrontal circuitry in bipolar-i and bipolar-nos youth**. *J Am Acad Child Adolesc Psychiatry* 2011, **50**(12):1275-1289 e1272.

200. Ladouceur CD, Diwadkar VA, White R, Bass J, Birmaher B, Axelson DA, Phillips ML: **Fronto-limbic function in unaffected offspring at familial risk for bipolar disorder during an emotional working memory paradigm**. *Dev Cogn Neurosci* 2013, **5**:185-196.

201. Laeger I, Dobel C, Dannlowski U, Kugel H, Grotegerd D, Kissler J, Keuper K, Eden A, Zwitserlood P, Zwanzger P: **Amygdala responsiveness to emotional words is modulated by subclinical anxiety and depression**. *Behav Brain Res* 2012, **233**(2):508-516.

202. Lamke JP, Daniels JK, Dorfel D, Gaebler M, Abdel Rahman R, Hummel F, Erk S, Walter H: **The impact of stimulus valence and emotion regulation on sustained brain activation: Task-rest switching in emotion**. *PLoS ONE* 2014, **9**(3):e93098.

203. Lang S, Kotchoubey B, Frick C, Spitzer C, Grabe HJ, Barnow S: **Cognitive reappraisal in trauma-exposed women with borderline personality disorder**. *Neuroimage* 2012, **59**(2):1727-1734.

204. Laurent HK, Ablow JC: **The missing link: Mothers' neural response to infant cry related to infant attachment behaviors**. *Infant Behav Dev* 2012, **35**(4):761-772.

205. Leclerc CM, Kensinger EA: **Age-related differences in medial prefrontal activation in response to emotional images**. *Cogn Affect Behav Neurosci* 2008, **8**(2):153-164.

206. Lee H, Heller AS, van Reekum CM, Nelson B, Davidson RJ: **Amygdala-prefrontal coupling underlies individual differences in emotion regulation**. *Neuroimage* 2012, **62**(3):1575-1581.

207. Lemenager T, Dieter J, Hill H, Hoffmann S, Reinhard I, Beutel M, Vollstadt-Klein S, Kiefer F, Mann K: **Exploring the neural basis of avatar identification in pathological internet gamers and of self-reflection in pathological social network users**. *Journal of behavioral addictions* 2016, **5**(3):485-499.

208. Lepping RJ, Atchley RA, Chrysikou E, Martin LE, Clair AA, Ingram RE, Simmons WK, Savage CR: **Neural processing of emotional musical and nonmusical stimuli in depression**. *PLoS ONE* 2016, **11**(6):e0156859.

209. Leutgeb V, Wabnegger A, Leitner M, Zussner T, Scharmuller W, Klug D, Schienle A: **Altered cerebellar-amygdala connectivity in violent offenders: A resting-state fmri study**. *Neurosci Lett* 2016, **610**:160-164.

210. Lévesque J, Joanette Y, Mensour B, Beaudoin G, Leroux JM, Bourgouin P, Beauregard M: **Neural basis of emotional self-regulation in childhood**. *Neuroscience* 2004, **129**(2):361-369.

211. Li C-sR, Huang C, Yan P, Bhagwagar Z, Milivojevic V, Sinha R: **Neural correlates of impulse control during stop signal inhibition in cocaine-dependent men**. *Neuropsychopharmacology* 2007, **33**(8):1798-1806.

212. Li Z, Tong L, Guan M, He W, Wang L, Bu H, Shi D, Yan B: **Altered resting-state amygdala functional connectivity after real-time fmri emotion self-regulation training**. *Biomed Res Int* 2016, **2016**:2719895.

213. Li Z, Tong L, Wang L, Li Y, He W, Guan M, Yan B: **Self-regulating positive emotion networks by feedback of multiple emotional brain states using real-time fmri**. *Exp Brain Res* 2016, **234**(12):3575-3586.

214. Lueken U, Straube B, Wittchen HU, Konrad C, Strohle A, Wittmann A, Pfleiderer B, Arolt V, Kircher T, Deckert J *et al*: **Therapygenetics: Anterior cingulate cortex-amygdala coupling is associated with 5-httlpr and treatment response in panic disorder with agoraphobia**. *J Neural Transm* 2014.

215. Luo S, Yu D, Han S: **Genetic and neural correlates of romantic relationship satisfaction**. *Soc Cogn Affect Neurosci* 2016, **11**(2):337-348.

216. Lutz J, Herwig U, Opialla S, Hittmeyer A, Jancke L, Rufer M, Grosse Holtforth M, Bruhl AB: **Mindfulness and emotion regulation--an fmri study**. *Soc Cogn Affect Neurosci* 2014, **9**(6):776-785.

217. Lutz J, Bruhl AB, Scheerer H, Jancke L, Herwig U: **Neural correlates of mindful self-awareness in mindfulness meditators and meditation-naive subjects revisited**. *Biol Psychol* 2016, **119**:21-30.

218. Lutz J, Bruhl AB, Doerig N, Scheerer H, Achermann R, Weibel A, Jancke L, Herwig U: **Altered processing of self-related emotional stimuli in mindfulness meditators**. *Neuroimage* 2016, **124**(Pt A):958-967.

219. MacNamara A, Rabinak CA, Kennedy AE, Fitzgerald DA, Liberzon I, Stein MB, Phan KL: **Emotion regulatory brain function and ssri treatment in ptsd: Neural correlates and predictors of change**. *Neuropsychopharmacology* 2016, **41**(2):611-618.

220. Majdandzic J, Bauer H, Windischberger C, Moser E, Engl E, Lamm C: **The human factor: Behavioral and neural correlates of humanized perception in moral decision making**. *PLoS ONE* 2012, **7**(10):e47698.

221. Mak AK, Hu ZG, Zhang JX, Xiao Z, Lee TM: **Sex-related differences in neural activity during emotion regulation**. *Neuropsychologia* 2009, **47**(13):2900-2908.

222. Makovac E, Watson DR, Meeten F, Garfinkel SN, Cercignani M, Critchley HD, Ottaviani C: **Amygdala functional connectivity as a longitudinal biomarker of symptom changes in generalized anxiety**. *Soc Cogn Affect Neurosci* 2016, **11**(11):1719-1728.

223. Manelis A, Ladouceur CD, Graur S, Monk K, Bonar LK, Hickey MB, Dwojak AC, Axelson D, Goldstein BI, Goldstein TR *et al*: **Altered amygdala-prefrontal response to facial emotion in offspring of parents with bipolar disorder**. *Brain* 2015, **138**(Pt 9):2777-2790.

224. Mansson KN, Carlbring P, Frick A, Engman J, Olsson CJ, Bodlund O, Furmark T, Andersson G: **Altered neural correlates of affective processing after internet-delivered cognitive behavior therapy for social anxiety disorder**. *Psychiatry Res* 2013, **214**(3):229-237.

225. Martucci KT, Shirer WR, Bagarinao E, Johnson KA, Farmer MA, Labus JS, Apkarian AV, Deutsch G, Harris RE, Mayer EA *et al*: **The posterior medial cortex in urologic chronic pelvic pain syndrome: Detachment from default mode network-a resting-state study from the mapp research network**. *Pain* 2015, **156**(9):1755-1764.

226. Marusak HA, Martin KR, Etkin A, Thomason ME: **Childhood trauma exposure disrupts the automatic regulation of emotional processing**. *Neuropsychopharmacology* 2015, **40**(5):1250-1258.

227. Mascaro JS, Hackett PD, Gouzoules H, Lori A, Rilling JK: **Behavioral and genetic correlates of the neural response to infant crying among human fathers**. *Soc Cogn Affect Neurosci* 2014, **9**(11):1704-1712.

228. Masten CL, Eisenberger NI, Pfeifer JH, Dapretto M: **Neural responses to witnessing peer rejection after being socially excluded: Fmri as a window into adolescents' emotional processing**. *Dev Sci* 2013, **16**(5):743-759.

229. Mazza M, Tempesta D, Pino MC, Catalucci A, Gallucci M, Ferrara M: **Regional cerebral changes and functional connectivity during the observation of negative emotional stimuli in subjects with post-traumatic stress disorder**. *Eur Arch Psychiatry Clin Neurosci* 2013, **263**(7):575-583.

230. McRae K, Hughes B, Chopra S, Gabrieli JD, Gross JJ, Ochsner KN: **The neural bases of distraction and reappraisal**. *J Cogn Neurosci* 2010, **22**(2):248-262.

231. McRae K, Gross JJ, Weber J, Robertson ER, Sokol-Hessner P, Ray RD, Gabrieli JD, Ochsner KN: **The development of emotion regulation: An fmri study of cognitive reappraisal in children, adolescents and young adults**. *Soc Cogn Affect Neurosci* 2012, **7**(1):11-22.

232. Meyer ML, Berkman ET, Karremans JC, Lieberman MD: **Incidental regulation of attraction: The neural basis of the derogation of attractive alternatives in romantic relationships**. *Cogn Emot* 2011, **25**(3):490-505.

233. Micoulaud-Franchi JA, Fakra E, Cermolacce M, Vion-Dury J: **[towards a new approach of neurophysiology in clinical psychiatry: Functional magnetic resonance imaging neurofeedback applied to emotional dysfunctions]**. *Neurophysiol Clin* 2012, **42**(3):79-94.

234. Minkel JD, McNealy K, Gianaros PJ, Drabant EM, Gross JJ, Manuck SB, Hariri AR: **Sleep quality and neural circuit function supporting emotion regulation**. *Biol Mood Anxiety Disord* 2012, **2**(1):22.

235. Mocking RJ, Figueroa CA, Rive MM, Geugies H, Servaas MN, Assies J, Koeter MW, Vaz FM, Wichers M, van Straalen JP *et al*: **Vulnerability for new episodes in recurrent major depressive disorder: Protocol for the longitudinal delta-neuroimaging cohort study**. *BMJ Open* 2016, **6**(3):e009510.

236. Mohanty A, Engels AS, Herrington JD, Heller W, Ho MH, Banich MT, Webb AG, Warren SL, Miller GA: **Differential engagement of anterior cingulate cortex subdivisions for cognitive and emotional function**. *Psychophysiology* 2007, **44**(3):343-351.

237. Morawetz C, Bode S, Baudewig J, Jacobs AM, Heekeren HR: **Neural representation of emotion regulation goals**. *Hum Brain Mapp* 2016, **37**(2):600-620.

238. Morawetz C, Bode S, Baudewig J, Kirilina E, Heekeren HR: **Changes in effective connectivity between dorsal and ventral prefrontal regions moderate emotion regulation**. *Cereb Cortex* 2016, **26**(5):1923-1937.

239. Morawetz C, Kellermann T, Kogler L, Radke S, Blechert J, Derntl B: **Intrinsic functional connectivity underlying successful emotion regulation of angry faces**. *Soc Cogn Affect Neurosci* 2016, **11**(12):1980-1991.

240. Moriguchi Y, Ohnishi T, Lane RD, Maeda M, Mori T, Nemoto K, Matsuda H, Komaki G: **Impaired self-awareness and theory of mind: An fmri study of mentalizing in alexithymia**. *Neuroimage* 2006, **32**(3):1472-1482.

241. Morris RW, Sparks A, Mitchell PB, Weickert CS, Green MJ: **Lack of cortico-limbic coupling in bipolar disorder and schizophrenia during emotion regulation**. *Transl Psychiatry* 2012, **2**:e90.

242. Morris JA, Leclerc CM, Kensinger EA: **Effects of valence and divided attention on cognitive reappraisal processes**. *Soc Cogn Affect Neurosci* 2014, **9**(12):1952-1961.

243. Moser DA, Aue T, Wang Z, Rusconi Serpa S, Favez N, Peterson BS, Schechter DS: **Limbic brain responses in mothers with post-traumatic stress disorder and comorbid dissociation to video clips of their children**. *Stress* 2013, **16**(5):493-502.

244. Moser DA, Aue T, Suardi F, Kutlikova H, Cordero MI, Rossignol AS, Favez N, Rusconi Serpa S, Schechter DS: **Violence-related ptsd and neural activation when seeing emotionally charged male-female interactions**. *Soc Cogn Affect Neurosci* 2015, **10**(5):645-653.

245. Moser DA, Paoloni-Giacobino A, Stenz L, Adouan W, Manini A, Suardi F, Cordero MI, Vital M, Sancho Rossignol A, Rusconi-Serpa S *et al*: **Bdnf methylation and maternal brain activity in a violence-related sample**. *PLoS ONE* 2015, **10**(12):e0143427.

246. Moutsiana C, Fearon P, Murray L, Cooper P, Goodyer I, Johnstone T, Halligan S: **Making an effort to feel positive: Insecure attachment in infancy predicts the neural underpinnings of emotion regulation in adulthood**. *J Child Psychol Psychiatry* 2014.

247. Mukherjee P, Sabharwal A, Kotov R, Szekely A, Parsey R, Barch DM, Mohanty A: **Disconnection between amygdala and medial prefrontal cortex in psychotic disorders**. *Schizophr Bull* 2016, **42**(4):1056-1067.

248. Mulej Bratec S, Xie X, Schmid G, Doll A, Schilbach L, Zimmer C, Wohlschlager A, Riedl V, Sorg C: **Cognitive emotion regulation enhances aversive prediction error activity while reducing emotional responses**. *Neuroimage* 2015, **123**:138-148.

249. Murakami H, Katsunuma R, Oba K, Terasawa Y, Motomura Y, Mishima K, Moriguchi Y: **Neural networks for mindfulness and emotion suppression**. *PLoS ONE* 2015, **10**(6):e0128005.

250. Murphy ER, Barch DM, Pagliaccio D, Luby JL, Belden AC: **Functional connectivity of the amygdala and subgenual cingulate during cognitive reappraisal of emotions in children with mdd history is associated with rumination**. *Dev Cogn Neurosci* 2016, **18**:89-100.

251. Nelson EE, Vinton DT, Berghorst L, Towbin KE, Hommer RE, Dickstein DP, Rich BA, Brotman MA, Pine DS, Leibenluft E: **Brain systems underlying response flexibility in healthy and bipolar adolescents: An event-related fmri study**. *Bipolar Disord* 2007, **9**(8):810-819.

252. New AS, Fan J, Murrough JW, Liu X, Liebman RE, Guise KG, Tang CY, Charney DS: **A functional magnetic resonance imaging study of deliberate emotion regulation in resilience and posttraumatic stress disorder**. *Biol Psychiatry* 2009, **66**(7):656-664.

253. Nicholson AA, Densmore M, Frewen PA, Theberge J, Neufeld RW, McKinnon MC, Lanius RA: **The dissociative subtype of posttraumatic stress disorder: Unique resting-state functional connectivity of basolateral and centromedial amygdala complexes**. *Neuropsychopharmacology* 2015, **40**(10):2317-2326.

254. Nicholson AA, Ros T, Frewen PA, Densmore M, Theberge J, Kluetsch RC, Jetly R, Lanius RA: **Alpha oscillation neurofeedback modulates amygdala complex connectivity and arousal in posttraumatic stress disorder**. *Neuroimage Clin* 2016, **12**:506-516.

255. Niedtfeld I, Schulze L, Kirsch P, Herpertz SC, Bohus M, Schmahl C: **Affect regulation and pain in borderline personality disorder: A possible link to the understanding of self-injury**. *Biol Psychiatry* 2010, **68**(4):383-391.

256. Niedtfeld I, Schmitt R, Winter D, Bohus M, Schmahl C, Herpertz SC: **Pain-mediated affect regulation is reduced after dialectical behavior therapy in borderline personality disorder: A longitudinal fmri study**. *Soc Cogn Affect Neurosci* 2017, **12**(5):739-747.

257. Olatunji BO, Ferreira-Garcia R, Caseras X, Fullana MA, Wooderson S, Speckens A, Lawrence N, Giampietro V, Brammer MJ, Phillips ML *et al*: **Predicting response to cognitive behavioral therapy in contamination-based obsessive-compulsive disorder from functional magnetic resonance imaging**. *Psychol Med* 2014, **44**(10):2125-2137.

258. O'Neill A, D'Souza A, Samson AC, Carballedo A, Kerskens C, Frodl T: **Dysregulation between emotion and theory of mind networks in borderline personality disorder**. *Psychiatry Res* 2015, **231**(1):25-32.

259. Opel N, Redlich R, Grotegerd D, Dohm K, Haupenthal C, Heindel W, Kugel H, Arolt V, Dannlowski U: **Enhanced neural responsiveness to reward associated with obesity in the absence of food-related stimuli**. *Hum Brain Mapp* 2015, **36**(6):2330-2337.

260. Opialla S, Lutz J, Scherpiet S, Hittmeyer A, Jancke L, Rufer M, Grosse Holtforth M, Herwig U, Bruhl AB: **Neural circuits of emotion regulation: A comparison of mindfulness-based and cognitive reappraisal strategies**. *Eur Arch Psychiatry Clin Neurosci* 2015, **265**(1):45-55.

261. Ossewaarde L, Hermans EJ, van Wingen GA, Kooijman SC, Johansson IM, Backstrom T, Fernandez G: **Neural mechanisms underlying changes in stress-sensitivity across the menstrual cycle**. *Psychoneuroendocrinology* 2010, **35**(1):47-55.

262. Outhred T, Das P, Felmingham KL, Bryant RA, Nathan PJ, Malhi GS, Kemp AH: **Facilitation of emotion regulation with a single dose of escitalopram: A randomized fmri study**. *Psychiatry Res* 2015, **233**(3):451-457.

263. Outhred T, Das P, Dobson-Stone C, Felmingham KL, Bryant RA, Nathan PJ, Malhi GS, Kemp AH: **Impact of 5-httlpr on ssri serotonin transporter blockade during emotion regulation: A preliminary fmri study**. *J Affect Disord* 2016, **196**:11-19.

264. Pannekoek JN, Veer IM, van Tol MJ, van der Werff SJ, Demenescu LR, Aleman A, Veltman DJ, Zitman FG, Rombouts SA, van der Wee NJ: **Aberrant limbic and salience network resting-state functional connectivity in panic disorder without comorbidity**. *J Affect Disord* 2013, **145**(1):29-35.

265. Papini C, White TP, Montagna A, Brittain PJ, Froudist-Walsh S, Kroll J, Karolis V, Simonelli A, Williams SC, Murray RM *et al*: **Altered resting-state functional connectivity in emotion-processing brain regions in adults who were born very preterm**. *Psychol Med* 2016, **46**(14):3025-3039.

266. Paret C, Kluetsch R, Ruf M, Demirakca T, Hoesterey S, Ende G, Schmahl C: **Down-regulation of amygdala activation with real-time fmri neurofeedback in a healthy female sample**. *Front Behav Neurosci* 2014, **8**:299.

267. Paret C, Kluetsch R, Ruf M, Demirakca T, Kalisch R, Schmahl C, Ende G: **Transient and sustained bold signal time courses affect the detection of emotion-related brain activation in fmri**. *Neuroimage* 2014, **103**:522-532.

268. Paret C, Kluetsch R, Zaehringer J, Ruf M, Demirakca T, Bohus M, Ende G, Schmahl C: **Alterations of amygdala-prefrontal connectivity with real-time fmri neurofeedback in bpd patients**. *Soc Cogn Affect Neurosci* 2016, **11**(6):952-960.

269. Paret C, Ruf M, Gerchen MF, Kluetsch R, Demirakca T, Jungkunz M, Bertsch K, Schmahl C, Ende G: **Fmri neurofeedback of amygdala response to aversive stimuli enhances prefrontal-limbic brain connectivity**. *Neuroimage* 2016, **125**:182-188.

270. Passarotti AM, Ellis J, Wegbreit E, Stevens MC, Pavuluri MN: **Reduced functional connectivity of prefrontal regions and amygdala within affect and working memory networks in pediatric bipolar disorder**. *Brain Connect* 2012, **2**(6):320-334.

271. Payer DE, Lieberman MD, London ED: **Neural correlates of affect processing and aggression in methamphetamine dependence**. *Arch Gen Psychiatry* 2011, **68**(3):271-282.

272. Payer DE, Baicy K, Lieberman MD, London ED: **Overlapping neural substrates between intentional and incidental down-regulation of negative emotions**. *Emotion* 2012, **12**(2):229-235.

273. Perlman SB, Pelphrey KA: **Regulatory brain development: Balancing emotion and cognition**. *Soc Neurosci* 2010, **5**(5-6):533-542.

274. Perlman SB, Pelphrey KA: **Developing connections for affective regulation: Age-related changes in emotional brain connectivity**. *J Exp Child Psychol* 2011, **108**(3):607-620.

275. Perlman G, Simmons AN, Wu J, Hahn KS, Tapert SF, Max JE, Paulus MP, Brown GG, Frank GK, Campbell-Sills L *et al*: **Amygdala response and functional connectivity during emotion regulation: A study of 14 depressed adolescents**. *J Affect Disord* 2012, **139**(1):75-84.

276. Perlman SB, Jones BM, Wakschlag LS, Axelson D, Birmaher B, Phillips ML: **Neural substrates of child irritability in typically developing and psychiatric populations**. *Dev Cogn Neurosci* 2015, **14**:71-80.

277. Petrican R, Rosenbaum RS, Grady C: **Expressive suppression and neural responsiveness to nonverbal affective cues**. *Neuropsychologia* 2015, **77**:321-330.

278. Phillips ML, Medford N, Senior C, Bullmore ET, Suckling J, Brammer MJ, Andrew C, Sierra M, Williams SC, David AS: **Depersonalization disorder: Thinking without feeling**. *Psychiatry Res* 2001, **108**(3):145-160.

279. Pitskel NB, Bolling DZ, Kaiser MD, Crowley MJ, Pelphrey KA: **How grossed out are you? The neural bases of emotion regulation from childhood to adolescence**. *Dev Cogn Neurosci* 2011, **1**(3):324-337.

280. Pitskel NB, Bolling DZ, Kaiser MD, Pelphrey KA, Crowley MJ: **Neural systems for cognitive reappraisal in children and adolescents with autism spectrum disorder**. *Dev Cogn Neurosci* 2014, **10**:117-128.

281. Platt B, Campbell CA, James AC, Murphy SE, Cooper MJ, Lau JY: **Cognitive reappraisal of peer rejection in depressed versus non-depressed adolescents: Functional connectivity differences**. *J Psychiatr Res* 2015, **61**:73-80.

282. Plener PL, Bubalo N, Fladung AK, Ludolph AG, Lule D: **Prone to excitement: Adolescent females with non-suicidal self-injury (nssi) show altered cortical pattern to emotional and nss-related material**. *Psychiatry Res* 2012, **203**(2-3):146-152.

283. Prehn K, Korn CW, Bajbouj M, Klann-Delius G, Menninghaus W, Jacobs AM, Heekeren HR: **The neural correlates of emotion alignment in social interaction**. *Soc Cogn Affect Neurosci* 2015, **10**(3):435-443.

284. Preis MA, Schmidt-Samoa C, Dechent P, Kroener-Herwig B: **The effects of prior pain experience on neural correlates of empathy for pain: An fmri study**. *Pain* 2013, **154**(3):411-418.

285. Price RB, Allen KB, Silk JS, Ladouceur CD, Ryan ND, Dahl RE, Forbes EE, Siegle GJ: **Vigilance in the laboratory predicts avoidance in the real world: A dimensional analysis of neural, behavioral, and ecological momentary data in anxious youth**. *Dev Cogn Neurosci* 2016, **19**:128-136.

286. Puetz VB, Kohn N, Dahmen B, Zvyagintsev M, Schuppen A, Schultz RT, Heim CM, Fink GR, Herpertz-Dahlmann B, Konrad K: **Neural response to social rejection in children with early separation experiences**. *J Am Acad Child Adolesc Psychiatry* 2014, **53**(12):1328-1337.e1328.

287. Puetz VB, Viding E, Palmer A, Kelly PA, Lickley R, Koutoufa I, Sebastian CL, McCrory EJ: **Altered neural response to rejection-related words in children exposed to maltreatment**. *J Child Psychol Psychiatry* 2016, **57**(10):1165-1173.

288. Puglia MH, Lillard TS, Morris JP, Connelly JJ: **Epigenetic modification of the oxytocin receptor gene influences the perception of anger and fear in the human brain**. *Proc Natl Acad Sci U S A* 2015, **112**(11):3308-3313.

289. Radaelli D, Sferrazza Papa G, Vai B, Poletti S, Smeraldi E, Colombo C, Benedetti F: **Fronto-limbic disconnection in bipolar disorder**. *Eur Psychiatry* 2015, **30**(1):82-88.

290. Raij TT, Korkeila J, Joutsenniemi K, Saarni SI, Riekki TJ: **Association of stigma resistance with emotion regulation - functional magnetic resonance imaging and neuropsychological findings**. *Compr Psychiatry* 2014, **55**(3):727-735.

291. Rauch AV, Ter Horst L, Paul VG, Bauer J, Dannlowski U, Konrad C, Ohrmann P, Kugel H, Egloff B, Arolt V *et al*: **Influence of repressive coping style on cortical activation during encoding of angry faces**. *PLoS ONE* 2014, **9**(12):e112398.

292. Rausch A, Zhang W, Haak KV, Mennes M, Hermans EJ, van Oort E, van Wingen G, Beckmann CF, Buitelaar JK, Groen WB: **Altered functional connectivity of the amygdaloid input nuclei in adolescents and young adults with autism spectrum disorder: A resting state fmri study**. *Molecular autism* 2016, **7**:13.

293. Raz G, Jacob Y, Gonen T, Winetraub Y, Flash T, Soreq E, Hendler T: **Cry for her or cry with her: Context-dependent dissociation of two modes of cinematic empathy reflected in network cohesion dynamics**. *Soc Cogn Affect Neurosci* 2014, **9**(1):30-38.

294. Reidy BL, Hamann S, Inman C, Johnson KC, Brennan PA: **Decreased sleep duration is associated with increased fmri responses to emotional faces in children**. *Neuropsychologia* 2016, **84**:54-62.

295. Reinecke A, Thilo K, Filippini N, Croft A, Harmer CJ: **Predicting rapid response to cognitive-behavioural treatment for panic disorder: The role of hippocampus, insula, and dorsolateral prefrontal cortex**. *Behav Res Ther* 2014, **62**:120-128.

296. Reinecke A, Filippini N, Berna C, Western DG, Hanson B, Cooper MJ, Taggart P, Harmer CJ: **Effective emotion regulation strategies improve fmri and ecg markers of psychopathology in panic disorder: Implications for psychological treatment action**. *Transl Psychiatry* 2015, **5**:e673.

297. Reitz S, Kluetsch R, Niedtfeld I, Knorz T, Lis S, Paret C, Kirsch P, Meyer-Lindenberg A, Treede RD, Baumgartner U *et al*: **Incision and stress regulation in borderline personality disorder: Neurobiological mechanisms of self-injurious behaviour**. *Br J Psychiatry* 2015, **207**(2):165-172.

298. Reske M, Kellermann T, Habel U, Jon Shah N, Backes V, von Wilmsdorff M, Stocker T, Gaebel W, Schneider F: **Stability of emotional dysfunctions? A long-term fmri study in first-episode schizophrenia**. *J Psychiatr Res* 2007, **41**(11):918-927.

299. Rey G, Piguet C, Benders A, Favre S, Eickhoff SB, Aubry JM, Vuilleumier P: **Resting-state functional connectivity of emotion regulation networks in euthymic and non-euthymic bipolar disorder patients**. *Eur Psychiatry* 2016, **34**:56-63.

300. Richter A, Grimm S, Northoff G: **Lorazepam modulates orbitofrontal signal changes during emotional processing in catatonia**. *Hum Psychopharmacol* 2010, **25**(1):55-62.

301. Riem MM, van IMH, Tops M, Boksem MA, Rombouts SA, Bakermans-Kranenburg MJ: **No laughing matter: Intranasal oxytocin administration changes functional brain connectivity during exposure to infant laughter**. *Neuropsychopharmacology* 2012, **37**(5):1257-1266.

302. Ritchey M, Bessette-Symons B, Hayes SM, Cabeza R: **Emotion processing in the aging brain is modulated by semantic elaboration**. *Neuropsychologia* 2011, **49**(4):640-650.

303. Ritchey M, Dolcos F, Eddington KM, Strauman TJ, Cabeza R: **Neural correlates of emotional processing in depression: Changes with cognitive behavioral therapy and predictors of treatment response**. *J Psychiatr Res* 2011, **45**(5):577-587.

304. Rive MM, Mocking RJ, Koeter MW, van Wingen G, de Wit SJ, van den Heuvel OA, Veltman DJ, Ruhe HG, Schene AH: **State-dependent differences in emotion regulation between unmedicated bipolar disorder and major depressive disorder**. *JAMA Psychiatry* 2015, **72**(7):687-696.

305. Rive MM, Redlich R, Schmaal L, Marquand AF, Dannlowski U, Grotegerd D, Veltman DJ, Schene AH, Ruhe HG: **Distinguishing medication-free subjects with unipolar disorder from subjects with bipolar disorder: State matters**. *Bipolar Disord* 2016, **18**(7):612-623.

306. Roelofs K, Minelli A, Mars RB, van Peer J, Toni I: **On the neural control of social emotional behavior**. *Soc Cogn Affect Neurosci* 2009, **4**(1):50-58.

307. Romero-Rebollar C, Jimenez-Angeles L, Dragustinovis-Ruiz EA, Medina-Banuelos V: **Neural modulation in aversive emotion processing: An independent component analysis study**. *Comput Math Methods Med* 2016, **2016**:2816567.

308. Rosales-Lagarde A, Armony JL, Del Rio-Portilla Y, Trejo-Martinez D, Conde R, Corsi-Cabrera M: **Enhanced emotional reactivity after selective rem sleep deprivation in humans: An fmri study**. *Front Behav Neurosci* 2012, **6**:25.

309. Sadeh N, Spielberg JM, Miller MW, Milberg WP, Salat DH, Amick MM, Fortier CB, McGlinchey RE: **Neurobiological indicators of disinhibition in posttraumatic stress disorder**. *Hum Brain Mapp* 2015, **36**(8):3076-3086.

310. Salomons TV, Johnstone T, Backonja MM, Shackman AJ, Davidson RJ: **Individual differences in the effects of perceived controllability on pain perception: Critical role of the prefrontal cortex**. *J Cogn Neurosci* 2007, **19**(6):993-1003.

311. Sanchez TA, Mocaiber I, Erthal FS, Joffily M, Volchan E, Pereira MG, de Araujo DB, Oliveira L: **Amygdala responses to unpleasant pictures are influenced by task demands and positive affect trait**. *Front Hum Neurosci* 2015, **9**:107.

312. Schaefer SM, Jackson DC, Davidson RJ, Aguirre GK, Kimberg DY, Thompson-Schill SL: **Modulation of amygdalar activity by the conscious regulation of negative emotion**. *J Cogn Neurosci* 2002, **14**(6):913-921.

313. Scharmuller W, Wabnegger A, Schienle A: **Functional brain connectivity during fear of pain: A comparison between dental phobics and controls**. *Brain Connect* 2015, **5**(3):187-191.

314. Scherpiet S, Herwig U, Opialla S, Scheerer H, Habermeyer V, Jancke L, Bruhl AB: **Reduced neural differentiation between self-referential cognitive and emotional processes in women with borderline personality disorder**. *Psychiatry Res* 2015, **233**(3):314-323.

315. Scheuerecker J, Meisenzahl EM, Koutsouleris N, Roesner M, Schopf V, Linn J, Wiesmann M, Bruckmann H, Moller HJ, Frodl T: **Orbitofrontal volume reductions during emotion recognition in patients with major depression**. *J Psychiatry Neurosci* 2010, **35**(5):311-320.

316. Schienle A, Schafer A, Pignanelli R, Vaitl D: **Worry tendencies predict brain activation during aversive imagery**. *Neurosci Lett* 2009, **461**(3):289-292.

317. Schneider-Hassloff H, Straube B, Nuscheler B, Wemken G, Kircher T: **Adult attachment style modulates neural responses in a mentalizing task**. *Neuroscience* 2015, **303**:462-473.

318. Schulze L, Domes G, Kruger A, Berger C, Fleischer M, Prehn K, Schmahl C, Grossmann A, Hauenstein K, Herpertz SC: **Neuronal correlates of cognitive reappraisal in borderline patients with affective instability**. *Biol Psychiatry* 2011, **69**(6):564-573.

319. Schweizer S, Grahn J, Hampshire A, Mobbs D, Dalgleish T: **Training the emotional brain: Improving affective control through emotional working memory training**. *J Neurosci* 2013, **33**(12):5301-5311.

320. Schweizer S, Walsh ND, Stretton J, Dunn VJ, Goodyer IM, Dalgleish T: **Enhanced emotion regulation capacity and its neural substrates in those exposed to moderate childhood adversity**. *Soc Cogn Affect Neurosci* 2016, **11**(2):272-281.

321. Schwerdtner J, Sommer M, Weber T, Muller J: **[functional neuroanatomy of emotions]**. *Psychiatr Prax* 2004, **31 Suppl 1**:S66-67.

322. Seiferth NY, Pauly K, Kellermann T, Shah NJ, Ott G, Herpertz-Dahlmann B, Kircher T, Schneider F, Habel U: **Neuronal correlates of facial emotion discrimination in early onset schizophrenia**. *Neuropsychopharmacology* 2009, **34**(2):477-487.

323. Seitz J, Hueck M, Dahmen B, Schulte-Ruther M, Legenbauer T, Herpertz-Dahlmann B, Konrad K: **Attention network dysfunction in bulimia nervosa - an fmri study**. *PLoS ONE* 2016, **11**(9):e0161329.

324. Seo D, Olman CA, Haut KM, Sinha R, MacDonald AW, 3rd, Patrick CJ: **Neural correlates of preparatory and regulatory control over positive and negative emotion**. *Soc Cogn Affect Neurosci* 2014, **9**(4):494-504.

325. Seo D, Lacadie CM, Sinha R: **Neural correlates and connectivity underlying stress-related impulse control difficulties in alcoholism**. *Alcohol Clin Exp Res* 2016, **40**(9):1884-1894.

326. Servaas MN, Riese H, Ormel J, Aleman A: **The neural correlates of worry in association with individual differences in neuroticism**. *Hum Brain Mapp* 2014, **35**(9):4303-4315.

327. Servaas MN, Geerligs L, Bastiaansen JA, Renken RJ, Marsman JC, Nolte IM, Ormel J, Aleman A, Riese H: **Associations between genetic risk, functional brain network organization and neuroticism**. *Brain Imaging Behav* 2016.

328. Shen C, Wang J, Ma G, Zhu Q, He H, Ding Q, Fan H, Lu Y, Wang W: **Waking-hour cerebral activations in nightmare disorder: A resting-state functional magnetic resonance imaging study**. *Psychiatry Clin Neurosci* 2016, **70**(12):573-581.

329. Shi H, Wang X, Yi J, Zhu X, Zhang X, Yang J, Yao S: **Default mode network alterations during implicit emotional faces processing in first-episode, treatment-naive major depression patients**. *Front Psychol* 2015, **6**:1198.

330. Siegle GJ, Carter CS, Thase ME: **Use of fmri to predict recovery from unipolar depression with cognitive behavior therapy**. *Am J Psychiatry* 2006, **163**(4):735-738.

331. Siegle GJ, Steinhauer SR, Friedman ES, Thompson WS, Thase ME: **Remission prognosis for cognitive therapy for recurrent depression using the pupil: Utility and neural correlates**. *Biol Psychiatry* 2011, **69**(8):726-733.

332. Silvers JA, Shu J, Hubbard AD, Weber J, Ochsner KN: **Concurrent and lasting effects of emotion regulation on amygdala response in adolescence and young adulthood**. *Dev Sci* 2015, **18**(5):771-784.

333. Silvers JA, Weber J, Wager TD, Ochsner KN: **Bad and worse: Neural systems underlying reappraisal of high- and low-intensity negative emotions**. *Soc Cogn Affect Neurosci* 2015, **10**(2):172-179.

334. Silvers JA, Hubbard AD, Biggs E, Shu J, Fertuck E, Chaudhury S, Grunebaum MF, Weber J, Kober H, Chesin M *et al*: **Affective lability and difficulties with regulation are differentially associated with amygdala and prefrontal response in women with borderline personality disorder**. *Psychiatry Res* 2016, **254**:74-82.

335. Silvers JA, Hubbard AD, Chaudhury S, Biggs E, Shu J, Grunebaum MF, Fertuck E, Weber J, Kober H, Carson-Wong A *et al*: **Suicide attempters with borderline personality disorder show differential orbitofrontal and parietal recruitment when reflecting on aversive memories**. *J Psychiatr Res* 2016, **81**:71-78.

336. Simon D, Adler N, Kaufmann C, Kathmann N: **Amygdala hyperactivation during symptom provocation in obsessive-compulsive disorder and its modulation by distraction**. *Neuroimage Clin* 2014, **4**:549-557.

337. Singh MK, Chang KD, Kelley RG, Saggar M, Reiss AL, Gotlib IH: **Early signs of anomalous neural functional connectivity in healthy offspring of parents with bipolar disorder**. *Bipolar Disord* 2014, **16**(7):678-689.

338. Sladky R, Hoflich A, Kublbock M, Kraus C, Baldinger P, Moser E, Lanzenberger R, Windischberger C: **Disrupted effective connectivity between the amygdala and orbitofrontal cortex in social anxiety disorder during emotion discrimination revealed by dynamic causal modeling for fmri**. *Cereb Cortex* 2015, **25**(4):895-903.

339. Smoski MJ, Salsman N, Wang L, Smith V, Lynch TR, Dager SR, LaBar KS, Linehan MM: **Functional imaging of emotion reactivity in opiate-dependent borderline personality disorder**. *Personal Disord* 2011, **2**(3):230-241.

340. Smoski MJ, Keng SL, Schiller CE, Minkel J, Dichter GS: **Neural mechanisms of cognitive reappraisal in remitted major depressive disorder**. *J Affect Disord* 2013, **151**(1):171-177.

341. Smoski MJ, Keng SL, Ji JL, Moore T, Minkel J, Dichter GS: **Neural indicators of emotion regulation via acceptance vs reappraisal in remitted major depressive disorder**. *Soc Cogn Affect Neurosci* 2015, **10**(9):1187-1194.

342. Sokol-Hessner P, Camerer CF, Phelps EA: **Emotion regulation reduces loss aversion and decreases amygdala responses to losses**. *Soc Cogn Affect Neurosci* 2013, **8**(3):341-350.

343. Song H, Zou Z, Kou J, Liu Y, Yang L, Zilverstand A, d'Oleire Uquillas F, Zhang X: **Love-related changes in the brain: A resting-state functional magnetic resonance imaging study**. *Front Hum Neurosci* 2015, **9**:71.

344. Sripada RK, King AP, Garfinkel SN, Wang X, Sripada CS, Welsh RC, Liberzon I: **Altered resting-state amygdala functional connectivity in men with posttraumatic stress disorder**. *J Psychiatry Neurosci* 2012, **37**(4):241-249.

345. Sripada CS, Phan KL, Labuschagne I, Welsh R, Nathan PJ, Wood AG: **Oxytocin enhances resting-state connectivity between amygdala and medial frontal cortex**. *Int J Neuropsychopharmacol* 2013, **16**(2):255-260.

346. Sripada RK, Marx CE, King AP, Rajaram N, Garfinkel SN, Abelson JL, Liberzon I: **Dhea enhances emotion regulation neurocircuits and modulates memory for emotional stimuli**. *Neuropsychopharmacology* 2013, **38**(9):1798-1807.

347. Sripada RK, Marx CE, King AP, Rampton JC, Ho SS, Liberzon I: **Allopregnanolone elevations following pregnenolone administration are associated with enhanced activation of emotion regulation neurocircuits**. *Biol Psychiatry* 2013, **73**(11):1045-1053.

348. Staudinger MR, Erk S, Walter H: **Dorsolateral prefrontal cortex modulates striatal reward encoding during reappraisal of reward anticipation**. *Cereb Cortex* 2011, **21**(11):2578-2588.

349. Stevens JS, Jovanovic T, Fani N, Ely TD, Glover EM, Bradley B, Ressler KJ: **Disrupted amygdala-prefrontal functional connectivity in civilian women with posttraumatic stress disorder**. *J Psychiatr Res* 2013, **47**(10):1469-1478.

350. Steward T, Pico-Perez M, Mata F, Martinez-Zalacain I, Cano M, Contreras-Rodriguez O, Fernandez-Aranda F, Yucel M, Soriano-Mas C, Verdejo-Garcia A: **Emotion regulation and excess weight: Impaired affective processing characterized by dysfunctional insula activation and connectivity**. *PLoS ONE* 2016, **11**(3):e0152150.

351. Sukhodolsky DG, Vander Wyk BC, Eilbott JA, McCauley SA, Ibrahim K, Crowley MJ, Pelphrey KA: **Neural mechanisms of cognitive-behavioral therapy for aggression in children and adolescents: Design of a randomized controlled trial within the national institute for mental health research domain criteria construct of frustrative non-reward**. *J Child Adolesc Psychopharmacol* 2016, **26**(1):38-48.

352. Surguladze SA, Radua J, El-Hage W, Gohier B, Sato JR, Kronhaus DM, Proitsi P, Powell J, Phillips ML: **Interaction of catechol o-methyltransferase and serotonin transporter genes modulates effective connectivity in a facial emotion-processing circuitry**. *Transl Psychiatry* 2012, **2**:e70.

353. Swartz JR, Carrasco M, Wiggins JL, Thomason ME, Monk CS: **Age-related changes in the structure and function of prefrontal cortex-amygdala circuitry in children and adolescents: A multi-modal imaging approach**. *Neuroimage* 2014, **86**:212-220.

354. Swartz JR, Phan KL, Angstadt M, Fitzgerald KD, Monk CS: **Dynamic changes in amygdala activation and functional connectivity in children and adolescents with anxiety disorders**. *Dev Psychopathol* 2014, **26**(4 Pt 2):1305-1319.

355. Tabibnia G, Creswell JD, Kraynak T, Westbrook C, Julson E, Tindle HA: **Common prefrontal regions activate during self-control of craving, emotion, and motor impulses in smokers**. *Clinical Psychological Science* 2014, **2**(5):611-619.

356. Tadayonnejad R, Yang S, Kumar A, Ajilore O: **Multimodal brain connectivity analysis in unmedicated late-life depression**. *PLoS ONE* 2014, **9**(4):e96033.

357. Tamura M, Moriguchi Y, Higuchi S, Hida A, Enomoto M, Umezawa J, Mishima K: **Activity in the action observation network enhances emotion regulation during observation of risk-taking: An fmri study**. *Neurol Res* 2013, **35**(1):22-28.

358. Tang A, Beaton EA, Tatham E, Schulkin J, Hall GB, Schmidt LA: **Processing of different types of social threat in shyness: Preliminary findings of distinct functional neural connectivity**. *Soc Neurosci* 2016, **11**(1):15-37.

359. Tao H, Guo S, Ge T, Kendrick KM, Xue Z, Liu Z, Feng J: **Depression uncouples brain hate circuit**. *Mol Psychiatry* 2013, **18**(1):101-111.

360. Taylor SE, Eisenberger NI, Saxbe D, Lehman BJ, Lieberman MD: **Neural responses to emotional stimuli are associated with childhood family stress**. *Biol Psychiatry* 2006, **60**(3):296-301.

361. Thomaes K, Dorrepaal E, Draijer N, de Ruiter MB, Elzinga BM, Sjoerds Z, van Balkom AJ, Smit JH, Veltman DJ: **Increased anterior cingulate cortex and hippocampus activation in complex ptsd during encoding of negative words**. *Soc Cogn Affect Neurosci* 2013, **8**(2):190-200.

362. Toazza R, Franco AR, Buchweitz A, Molle RD, Rodrigues DM, Reis RS, Mucellini AB, Esper NB, Aguzzoli C, Silveira PP *et al*: **Amygdala-based intrinsic functional connectivity and anxiety disorders in adolescents and young adults**. *Psychiatry Res* 2016, **257**:11-16.

363. Torrisi S, Moody TD, Vizueta N, Thomason ME, Monti MM, Townsend JD, Bookheimer SY, Altshuler LL: **Differences in resting corticolimbic functional connectivity in bipolar i euthymia**. *Bipolar Disord* 2013, **15**(2):156-166.

364. Torrisi SJ, Lieberman MD, Bookheimer SY, Altshuler LL: **Advancing understanding of affect labeling with dynamic causal modeling**. *Neuroimage* 2013, **82**:481-488.

365. Townsend JD, Torrisi SJ, Lieberman MD, Sugar CA, Bookheimer SY, Altshuler LL: **Frontal-amygdala connectivity alterations during emotion downregulation in bipolar i disorder**. *Biol Psychiatry* 2013, **73**(2):127-135.

366. Tromp DP, Grupe DW, Oathes DJ, McFarlin DR, Hernandez PJ, Kral TR, Lee JE, Adams M, Alexander AL, Nitschke JB: **Reduced structural connectivity of a major frontolimbic pathway in generalized anxiety disorder**. *Arch Gen Psychiatry* 2012, **69**(9):925-934.

367. Uchida M, Biederman J, Gabrieli JD, Micco J, de Los Angeles C, Brown A, Kenworthy T, Kagan E, Whitfield-Gabrieli S: **Emotion regulation ability varies in relation to intrinsic functional brain architecture**. *Soc Cogn Affect Neurosci* 2015, **10**(12):1738-1748.

368. Urry HL, van Reekum CM, Johnstone T, Kalin NH, Thurow ME, Schaefer HS, Jackson CA, Frye CJ, Greischar LL, Alexander AL *et al*: **Amygdala and ventromedial prefrontal cortex are inversely coupled during regulation of negative affect and predict the diurnal pattern of cortisol secretion among older adults**. *J Neurosci* 2006, **26**(16):4415-4425.

369. Urry HL, van Reekum CM, Johnstone T, Davidson RJ: **Individual differences in some (but not all) medial prefrontal regions reflect cognitive demand while regulating unpleasant emotion**. *Neuroimage* 2009, **47**(3):852-863.

370. Van den Stock J, De Winter FL, Ahmad R, Sunaert S, Van Laere K, Vandenberghe W, Vandenbulcke M: **Functional brain changes underlying irritability in premanifest huntington's disease**. *Hum Brain Mapp* 2015, **36**(7):2681-2690.

371. van der Horn HJ, Liemburg EJ, Scheenen ME, de Koning ME, Marsman JB, Spikman JM, van der Naalt J: **Brain network dysregulation, emotion, and complaints after mild traumatic brain injury**. *Hum Brain Mapp* 2016, **37**(4):1645-1654.

372. van Reekum CM, Johnstone T, Urry HL, Thurow ME, Schaefer HS, Alexander AL, Davidson RJ: **Gaze fixations predict brain activation during the voluntary regulation of picture-induced negative affect**. *Neuroimage* 2007, **36**(3):1041-1055.

373. Vanderhasselt MA, Kuhn S, De Raedt R: **Healthy brooders employ more attentional resources when disengaging from the negative: An event-related fmri study**. *Cogn Affect Behav Neurosci* 2011, **11**(2):207-216.

374. Vanderhasselt MA, Baeken C, Van Schuerbeek P, Luypaert R, De Raedt R: **Inter-individual differences in the habitual use of cognitive reappraisal and expressive suppression are associated with variations in prefrontal cognitive control for emotional information: An event related fmri study**. *Biol Psychol* 2013, **92**(3):433-439.

375. Vrticka P, Sander D, Vuilleumier P: **Effects of emotion regulation strategy on brain responses to the valence and social content of visual scenes**. *Neuropsychologia* 2011, **49**(5):1067-1082.

376. Vrticka P, Bondolfi G, Sander D, Vuilleumier P: **The neural substrates of social emotion perception and regulation are modulated by adult attachment style**. *Soc Neurosci* 2012, **7**(5):473-493.

377. Vrticka P, Sander D, Vuilleumier P: **Lateralized interactive social content and valence processing within the human amygdala**. *Front Hum Neurosci* 2012, **6**:358.

378. Vrticka P, Simioni S, Fornari E, Schluep M, Vuilleumier P, Sander D: **Neural substrates of social emotion regulation: A fmri study on imitation and expressive suppression to dynamic facial signals**. *Front Psychol* 2013, **4**:95.

379. Wager TD, Davidson ML, Hughes BL, Lindquist MA, Ochsner KN: **Prefrontal-subcortical pathways mediating successful emotion regulation**. *Neuron* 2008, **59**(6):1037-1050.

380. Wagner DD, Heatherton TF: **Self-regulatory depletion increases emotional reactivity in the amygdala**. *Soc Cogn Affect Neurosci* 2013, **8**(4):410-417.

381. Wagner U, Galli L, Schott BH, Wold A, van der Schalk J, Manstead AS, Scherer K, Walter H: **Beautiful friendship: Social sharing of emotions improves subjective feelings and activates the neural reward circuitry**. *Soc Cogn Affect Neurosci* 2015, **10**(6):801-808.

382. Waring JD, Etkin A, Hallmayer JF, O'Hara R: **Connectivity underlying emotion conflict regulation in older adults with 5-httlpr short allele: A preliminary investigation**. *Am J Geriatr Psychiatry* 2014, **22**(9):946-950.

383. Warren SL, Bost KK, Roisman GI, Silton RL, Spielberg JM, Engels AS, Choi E, Sutton BP, Miller GA, Heller W: **Effects of adult attachment and emotional distractors on brain mechanisms of cognitive control**. *Psychol Sci* 2010, **21**(12):1818-1826.

384. Weidt S, Lutz J, Rufer M, Delsignore A, Jakob NJ, Herwig U, Bruehl AB: **Common and differential alterations of general emotion processing in obsessive-compulsive and social anxiety disorder**. *Psychol Med* 2016, **46**(7):1427-1436.

385. Weng HY, Fox AS, Shackman AJ, Stodola DE, Caldwell JZ, Olson MC, Rogers GM, Davidson RJ: **Compassion training alters altruism and neural responses to suffering**. *Psychol Sci* 2013, **24**(7):1171-1180.

386. Westen D, Blagov PS, Harenski K, Kilts C, Hamann S: **Neural bases of motivated reasoning: An fmri study of emotional constraints on partisan political judgment in the 2004 U.S. Presidential election**. *J Cogn Neurosci* 2006, **18**(11):1947-1958.

387. Wheelock MD, Sreenivasan KR, Wood KH, Ver Hoef LW, Deshpande G, Knight DC: **Threat-related learning relies on distinct dorsal prefrontal cortex network connectivity**. *Neuroimage* 2014, **102 Pt 2**:904-912.

388. Wiech K, Farias M, Kahane G, Shackel N, Tiede W, Tracey I: **An fmri study measuring analgesia enhanced by religion as a belief system**. *Pain* 2008, **139**(2):467-476.

389. Wiech K, Edwards R, Moseley GL, Berna C, Ploner M, Tracey I: **Dissociable neural mechanisms underlying the modulation of pain and anxiety? An fmri pilot study**. *PLoS ONE* 2014, **9**(12):e110654.

390. Woo YK, Song J, Jiang Y, Cho C, Bong M, Kim SI: **Effects of informative and confirmatory feedback on brain activation during negative feedback processing**. *Front Hum Neurosci* 2015, **9**:378.

391. Wood KH, Wheelock MD, Shumen JR, Bowen KH, Ver Hoef LW, Knight DC: **Controllability modulates the neural response to predictable but not unpredictable threat in humans**. *Neuroimage* 2015, **119**:371-381.

392. Wu X, Lin P, Yang J, Song H, Yang R, Yang J: **Dysfunction of the cingulo-opercular network in first-episode medication-naive patients with major depressive disorder**. *J Affect Disord* 2016, **200**:275-283.

393. Xie X, Mulej Bratec S, Schmid G, Meng C, Doll A, Wohlschlager A, Finke K, Forstl H, Zimmer C, Pekrun R *et al*: **How do you make me feel better? Social cognitive emotion regulation and the default mode network**. *Neuroimage* 2016, **134**:270-280.

394. Xiong K, Zhang Y, Qiu M, Zhang J, Sang L, Wang L, Xie B, Wang J, Li M: **Negative emotion regulation in patients with posttraumatic stress disorder**. *PLoS ONE* 2013, **8**(12):e81957.

395. Yang W, Chen Q, Liu P, Cheng H, Cui Q, Wei D, Zhang Q, Qiu J: **Abnormal brain activation during directed forgetting of negative memory in depressed patients**. *J Affect Disord* 2016, **190**:880-888.

396. Yao S, Becker B, Geng Y, Zhao Z, Xu X, Zhao W, Ren P, Kendrick KM: **Voluntary control of anterior insula and its functional connections is feedback-independent and increases pain empathy**. *Neuroimage* 2016, **130**:230-240.

397. Yoon H, Kim SA, Kim SH: **Facial expression primes and implicit regulation of negative emotion**. *Neuroreport* 2015, **26**(9):548-553.

398. Yoshimura S, Okamoto Y, Yoshino A, Kobayakawa M, Machino A, Yamawaki S: **Neural basis of anticipatory anxiety reappraisals**. *PLoS ONE* 2014, **9**(7):e102836.

399. Yuan H, Young KD, Phillips R, Zotev V, Misaki M, Bodurka J: **Resting-state functional connectivity modulation and sustained changes after real-time functional magnetic resonance imaging neurofeedback training in depression**. *Brain Connect* 2014, **4**(9):690-701.

400. Zhang T, Li F, Beckes L, Coan JA: **A semi-parametric model of the hemodynamic response for multi-subject fmri data**. *Neuroimage* 2013, **75**:136-145.

401. Zhang W, Guo J, Zhang J, Luo J: **Neural mechanism of placebo effects and cognitive reappraisal in emotion regulation**. *Prog Neuropsychopharmacol Biol Psychiatry* 2013, **40**:364-373.

402. Zhang W, Li H, Chen J, Chen N, Liu X, Wang D, Shen J: **Posterior p1 and early frontal negativity reflect developmental changes in attentional distraction during adolescence**. *Brain Cogn* 2014, **87**:30-38.

403. Zhang X, Zhu X, Wang X, Zhu X, Zhong M, Yi J, Rao H, Yao S: **First-episode medication-naive major depressive disorder is associated with altered resting brain function in the affective network**. *PLoS ONE* 2014, **9**(1):e85241.

404. Zhao LY, Tian J, Wang W, Qin W, Shi J, Li Q, Yuan K, Dong MH, Yang WC, Wang YR *et al*: **The role of dorsal anterior cingulate cortex in the regulation of craving by reappraisal in smokers**. *PLoS ONE* 2012, **7**(8):e43598.

405. Ziv M, Goldin PR, Jazaieri H, Hahn KS, Gross JJ: **Is there less to social anxiety than meets the eye? Behavioral and neural responses to three socio-emotional tasks**. *Biol Mood Anxiety Disord* 2013, **3**(1):5.

406. Zotev V, Phillips R, Young KD, Drevets WC, Bodurka J: **Prefrontal control of the amygdala during real-time fmri neurofeedback training of emotion regulation**. *PLoS ONE* 2013, **8**(11):e79184.

407. Zotev V, Yuan H, Misaki M, Phillips R, Young KD, Feldner MT, Bodurka J: **Correlation between amygdala bold activity and frontal eeg asymmetry during real-time fmri neurofeedback training in patients with depression**. *Neuroimage Clin* 2016, **11**:224-238.

408. Zou L, Guo Q, Xu Y, Yang B, Jiao Z, Xiang J: **Functional connectivity analysis of the neural bases of emotion regulation: A comparison of independent component method with density-based k-means clustering method**. *Technol Health Care* 2016, **24 Suppl 2**:S817-825.

409. Abraham A, Thybusch K, Pieritz K, Hermann C: **Gender differences in creative thinking: behavioral and fMRI findings**. *Brain imaging and behavior* 2014, **8**(1):39-51.

410. Ahluwalia V, Wade JB, Heuman DM, Hammeke TA, Sanyal AJ, Sterling RK, Stravitz RT, Luketic V, Siddiqui MS, Puri P *et al*: **Enhancement of functional connectivity, working memory and inhibitory control on multi-modal brain MR imaging with Rifaximin in Cirrhosis: implications for the gut-liver-brain axis**. *Metabolic brain disease* 2014, **29**(4):1017-1025.

411. Aloia MS, Sweet LH, Jerskey BA, Zimmerman M, Arnedt JT, Millman RP: **Treatment effects on brain activity during a working memory task in obstructive sleep apnea**. *Journal of sleep research* 2009, **18**(4):404-410.

412. Andrews J, Wang L, Csernansky JG, Gado MH, Barch DM: **Abnormalities of thalamic activation and cognition in schizophrenia**. *Am J Psychiatry* 2006, **163**(3):463-469.

413. Archbold KH, Borghesani PR, Mahurin RK, Kapur VK, Landis CA: **Neural activation patterns during working memory tasks and OSA disease severity: preliminary findings**. *Journal of clinical sleep medicine : JCSM : official publication of the American Academy of Sleep Medicine* 2009, **5**(1):21-27.

414. Ashare RL, Valdez JN, Ruparel K, Albelda B, Hopson RD, Keefe JR, Loughead J, Lerman C: **Association of abstinence-induced alterations in working memory function and COMT genotype in smokers**. *Psychopharmacology* 2013, **230**(4):653-662.

415. Ashare RL, Wileyto EP, Ruparel K, Goelz PM, Hopson RD, Valdez JN, Gur RC, Loughead J, Lerman C: **Effects of tolcapone on working memory and brain activity in abstinent smokers: a proof-of-concept study**. *Drug Alcohol Depend* 2013, **133**(3):852-856.

416. Axmacher N, Bialleck KA, Weber B, Helmstaedter C, Elger CE, Fell J: **Working memory representation in atypical language dominance**. *Human brain mapping* 2009, **30**(7):2032-2043.

417. Bakshi N, Pruitt P, Radwan J, Keshavan MS, Rajan U, Zajac-Benitez C, Diwadkar VA: **Inefficiently increased anterior cingulate modulation of cortical systems during working memory in young offspring of schizophrenia patients**. *J Psychiatr Res* 2011, **45**(8):1067-1076.

418. Barch DM, Sheline YI, Csernansky JG, Snyder AZ: **Working memory and prefrontal cortex dysfunction: specificity to schizophrenia compared with major depression**. *Biol Psychiatry* 2003, **53**(5):376-384.

419. Barnes A, Bullmore ET, Suckling J: **Endogenous human brain dynamics recover slowly following cognitive effort**. *PloS one* 2009, **4**(8):e6626.

420. Bayerl M, Dielentheis TF, Vucurevic G, Gesierich T, Vogel F, Fehr C, Stoeter P, Huss M, Konrad A: **Disturbed brain activation during a working memory task in drug-naive adult patients with ADHD**. *Neuroreport* 2010, **21**(6):442-446.

421. Becerril K, Barch D: **Influence of emotional processing on working memory in schizophrenia**. *Schizophrenia bulletin* 2011, **37**(5):1027-1038.

422. Bechtel N, Kobel M, Penner IK, Specht K, Klarhofer M, Scheffler K, Opwis K, Schmitt-Mechelke T, Capone A, Weber P: **Attention-deficit/hyperactivity disorder in childhood epilepsy: a neuropsychological and functional imaging study**. *Epilepsia* 2012, **53**(2):325-333.

423. Bedard AC, Newcorn JH, Clerkin SM, Krone B, Fan J, Halperin JM, Schulz KP: **Reduced prefrontal efficiency for visuospatial working memory in attention-deficit/hyperactivity disorder**. *Journal of the American Academy of Child and Adolescent Psychiatry* 2014, **53**(9):1020-1030.e1026.

424. Beneventi H, Barndon R, Ersland L, Hugdahl K: **An fMRI study of working memory for schematic facial expressions**. *Scand J Psychol* 2007, **48**(2):81-86.

425. Beneventi H, Tonnessen FE, Ersland L, Hugdahl K: **Executive working memory processes in dyslexia: behavioral and fMRI evidence**. *Scand J Psychol* 2010, **51**(3):192-202.

426. Beneventi H, Tonnessen FE, Ersland L, Hugdahl K: **Working memory deficit in dyslexia: behavioral and FMRI evidence**. *Int J Neurosci* 2010, **120**(1):51-59.

427. Bengtsson SL, Dolan RJ, Passingham RE: **Priming for self-esteem influences the monitoring of one's own performance**. *Social cognitive and affective neuroscience* 2011, **6**(4):417-425.

428. Bennett DS, Mohamed FB, Carmody DP, Malik M, Faro SH, Lewis M: **Prenatal tobacco exposure predicts differential brain function during working memory in early adolescence: a preliminary investigation**. *Brain imaging and behavior* 2013, **7**(1):49-59.

429. Bernal-Casas D, Balaguer-Ballester E, Gerchen MF, Iglesias S, Walter H, Heinz A, Meyer-Lindenberg A, Stephan KE, Kirsch P: **Multi-site reproducibility of prefrontal-hippocampal connectivity estimates by stochastic DCM**. *NeuroImage* 2013, **82**:555-563.

430. Bertolino A, Caforio G, Blasi G, De Candia M, Latorre V, Petruzzella V, Altamura M, Nappi G, Papa S, Callicott JH *et al*: **Interaction of COMT (Val(108/158)Met) genotype and olanzapine treatment on prefrontal cortical function in patients with schizophrenia**. *Am J Psychiatry* 2004, **161**(10):1798-1805.

431. Bertolino A, Taurisano P, Pisciotta NM, Blasi G, Fazio L, Romano R, Gelao B, Lo Bianco L, Lozupone M, Di Giorgio A *et al*: **Genetically determined measures of striatal D2 signaling predict prefrontal activity during working memory performance**. *PloS one* 2010, **5**(2):e9348.

432. Bilek E, Schafer A, Ochs E, Esslinger C, Zangl M, Plichta MM, Braun U, Kirsch P, Schulze TG, Rietschel M *et al*: **Application of high-frequency repetitive transcranial magnetic stimulation to the DLPFC alters human prefrontal-hippocampal functional interaction**. *The Journal of neuroscience : the official journal of the Society for Neuroscience* 2013, **33**(16):7050-7056.

433. Bleich-Cohen M, Jamshy S, Sharon H, Weizman R, Intrator N, Poyurovsky M, Hendler T: **Machine learning fMRI classifier delineates subgroups of schizophrenia patients**. *Schizophr Res* 2014, **160**(1-3):196-200.

434. Blokland GA, McMahon KL, Hoffman J, Zhu G, Meredith M, Martin NG, Thompson PM, de Zubicaray GI, Wright MJ: **Quantifying the heritability of task-related brain activation and performance during the N-back working memory task: a twin fMRI study**. *Biol Psychol* 2008, **79**(1):70-79.

435. Blokland GA, McMahon KL, Thompson PM, Hickie IB, Martin NG, de Zubicaray GI, Wright MJ: **Genetic effects on the cerebellar role in working memory: same brain, different genes?** *NeuroImage* 2014, **86**:392-403.

436. Brahmbhatt SB, McAuley T, Barch DM: **Functional developmental similarities and differences in the neural correlates of verbal and nonverbal working memory tasks**. *Neuropsychologia* 2008, **46**(4):1020-1031.

437. Brandt CL, Eichele T, Melle I, Sundet K, Server A, Agartz I, Hugdahl K, Jensen J, Andreassen OA: **Working memory networks and activation patterns in schizophrenia and bipolar disorder: comparison with healthy controls**. *The British journal of psychiatry : the journal of mental science* 2014, **204**:290-298.

438. Brechmann A, Gaschler-Markefski B, Sohr M, Yoneda K, Kaulisch T, Scheich H: **Working memory specific activity in auditory cortex: potential correlates of sequential processing and maintenance**. *Cerebral cortex (New York, NY : 1991)* 2007, **17**(11):2544-2552.

439. Brookes MJ, Wood JR, Stevenson CM, Zumer JM, White TP, Liddle PF, Morris PG: **Changes in brain network activity during working memory tasks: a magnetoencephalography study**. *NeuroImage* 2011, **55**(4):1804-1815.

440. Broome MR, Matthiasson P, Fusar-Poli P, Woolley JB, Johns LC, Tabraham P, Bramon E, Valmaggia L, Williams SC, Brammer MJ *et al*: **Neural correlates of executive function and working memory in the 'at-risk mental state'**. *The British journal of psychiatry : the journal of mental science* 2009, **194**(1):25-33.

441. Brown A, Biederman J, Valera E, Lomedico A, Aleardi M, Makris N, Seidman LJ: **Working memory network alterations and associated symptoms in adults with ADHD and Bipolar Disorder**. *J Psychiatr Res* 2012, **46**(4):476-483.

442. Burgess GC, Gray JR, Conway AR, Braver TS: **Neural mechanisms of interference control underlie the relationship between fluid intelligence and working memory span**. *Journal of experimental psychology General* 2011, **140**(4):674-692.

443. Burzynska AZ, Garrett DD, Preuschhof C, Nagel IE, Li SC, Backman L, Heekeren HR, Lindenberger U: **A scaffold for efficiency in the human brain**. *The Journal of neuroscience : the official journal of the Society for Neuroscience* 2013, **33**(43):17150-17159.

444. Callicott JH, Bertolino A, Mattay VS, Langheim FJ, Duyn J, Coppola R, Goldberg TE, Weinberger DR: **Physiological dysfunction of the dorsolateral prefrontal cortex in schizophrenia revisited**. *Cerebral cortex (New York, NY : 1991)* 2000, **10**(11):1078-1092.

445. Callicott JH, Egan MF, Mattay VS, Bertolino A, Bone AD, Verchinksi B, Weinberger DR: **Abnormal fMRI response of the dorsolateral prefrontal cortex in cognitively intact siblings of patients with schizophrenia**. *Am J Psychiatry* 2003, **160**(4):709-719.

446. Callicott JH, Mattay VS, Verchinski BA, Marenco S, Egan MF, Weinberger DR: **Complexity of prefrontal cortical dysfunction in schizophrenia: more than up or down**. *Am J Psychiatry* 2003, **160**(12):2209-2215.

447. Campanella S, Peigneux P, Petit G, Lallemand F, Saeremans M, Noel X, Metens T, Nouali M, De Tiege X, De Witte P *et al*: **Increased cortical activity in binge drinkers during working memory task: a preliminary assessment through a functional magnetic resonance imaging study**. *PloS one* 2013, **8**(4):e62260.

448. Cao H, Plichta MM, Schafer A, Haddad L, Grimm O, Schneider M, Esslinger C, Kirsch P, Meyer-Lindenberg A, Tost H: **Test-retest reliability of fMRI-based graph theoretical properties during working memory, emotion processing, and resting state**. *NeuroImage* 2014, **84**:888-900.

449. Caseras X, Mataix-Cols D, Giampietro V, Rimes KA, Brammer M, Zelaya F, Chalder T, Godfrey EL: **Probing the working memory system in chronic fatigue syndrome: a functional magnetic resonance imaging study using the n-back task**. *Psychosomatic medicine* 2006, **68**(6):947-955.

450. Castronovo V, Canessa N, Strambi LF, Aloia MS, Consonni M, Marelli S, Iadanza A, Bruschi A, Falini A, Cappa SF: **Brain activation changes before and after PAP treatment in obstructive sleep apnea**. *Sleep* 2009, **32**(9):1161-1172.

451. Cerasa A, Gioia MC, Fera F, Passamonti L, Liguori M, Lanza P, Muglia M, Magariello A, Quattrone A: **Ventro-lateral prefrontal activity during working memory is modulated by MAO A genetic variation**. *Brain research* 2008, **1201**:114-121.

452. Chang K, Adleman NE, Dienes K, Simeonova DI, Menon V, Reiss A: **Anomalous prefrontal-subcortical activation in familial pediatric bipolar disorder: a functional magnetic resonance imaging investigation**. *Archives of general psychiatry* 2004, **61**(8):781-792.

453. Chantiluke K, Barrett N, Giampietro V, Brammer M, Simmons A, Rubia K: **Disorder-dissociated effects of fluoxetine on brain function of working memory in attention deficit hyperactivity disorder and autism spectrum disorder**. *Psychol Med* 2015, **45**(6):1195-1205.

454. Chao HH, Uchio E, Zhang S, Hu S, Bednarski SR, Luo X, Rose M, Concato J, Li CS: **Effects of androgen deprivation on brain function in prostate cancer patients - a prospective observational cohort analysis**. *BMC cancer* 2012, **12**:371.

455. Charlet K, Beck A, Jorde A, Wimmer L, Vollstadt-Klein S, Gallinat J, Walter H, Kiefer F, Heinz A: **Increased neural activity during high working memory load predicts low relapse risk in alcohol dependence**. *Addiction biology* 2014, **19**(3):402-414.

456. Chen CJ, Chen CC, Wu D, Chi NF, Chen PC, Liao YP, Chiu HW, Hu CJ: **Effects of the apolipoprotein E epsilon4 allele on functional MRI during n-back working memory tasks in healthy middle-aged adults**. *AJNR American journal of neuroradiology* 2013, **34**(6):1197-1202.

457. Chen Y, Liu Z, Zhang J, Xu K, Zhang S, Wei D, Zhang Z: **Altered brain activation patterns under different working memory loads in patients with type 2 diabetes**. *Diabetes care* 2014, **37**(12):3157-3163.

458. Choo WC, Lee WW, Venkatraman V, Sheu FS, Chee MW: **Dissociation of cortical regions modulated by both working memory load and sleep deprivation and by sleep deprivation alone**. *NeuroImage* 2005, **25**(2):579-587.

459. Christ SE, Moffitt AJ, Peck D: **Disruption of prefrontal function and connectivity in individuals with phenylketonuria**. *Molecular genetics and metabolism* 2010, **99 Suppl 1**:S33-40.

460. Ciesielski KT, Lesnik PG, Savoy RL, Grant EP, Ahlfors SP: **Developmental neural networks in children performing a Categorical N-Back Task**. *NeuroImage* 2006, **33**(3):980-990.

461. Colizzi M, Fazio L, Ferranti L, Porcelli A, Masellis R, Marvulli D, Bonvino A, Ursini G, Blasi G, Bertolino A: **Functional genetic variation of the cannabinoid receptor 1 and cannabis use interact on prefrontal connectivity and related working memory behavior**. *Neuropsychopharmacology : official publication of the American College of Neuropsychopharmacology* 2015, **40**(3):640-649.

462. Colorado RA, Shukla K, Zhou Y, Wolinsky JS, Narayana PA: **Multi-task functional MRI in multiple sclerosis patients without clinical disability**. *NeuroImage* 2012, **59**(1):573-581.

463. Cousijn H, Rijpkema M, Qin S, van Wingen GA, Fernandez G: **Phasic deactivation of the medial temporal lobe enables working memory processing under stress**. *NeuroImage* 2012, **59**(2):1161-1167.

464. Cousijn J, Vingerhoets WA, Koenders L, de Haan L, van den Brink W, Wiers RW, Goudriaan AE: **Relationship between working-memory network function and substance use: a 3-year longitudinal fMRI study in heavy cannabis users and controls**. *Addiction biology* 2014, **19**(2):282-293.

465. Cousijn J, Wiers RW, Ridderinkhof KR, van den Brink W, Veltman DJ, Goudriaan AE: **Effect of baseline cannabis use and working-memory network function on changes in cannabis use in heavy cannabis users: a prospective fMRI study**. *Human brain mapping* 2014, **35**(5):2470-2482.

466. Creswell JD, Bursley JK, Satpute AB: **Neural reactivation links unconscious thought to decision-making performance**. *Social cognitive and affective neuroscience* 2013, **8**(8):863-869.

467. Cservenka A, Herting MM, Nagel BJ: **Atypical frontal lobe activity during verbal working memory in youth with a family history of alcoholism**. *Drug Alcohol Depend* 2012, **123**(1-3):98-104.

468. Cubillo A, Smith AB, Barrett N, Giampietro V, Brammer M, Simmons A, Rubia K: **Drug-specific laterality effects on frontal lobe activation of atomoxetine and methylphenidate in attention deficit hyperactivity disorder boys during working memory**. *Psychol Med* 2014, **44**(3):633-646.

469. Daamen M, Bauml JG, Scheef L, Sorg C, Busch B, Baumann N, Bartmann P, Wolke D, Wohlschlager A, Boecker H: **Working memory in preterm-born adults: load-dependent compensatory activity of the posterior default mode network**. *Human brain mapping* 2015, **36**(3):1121-1137.

470. D'Aiuto L, Prasad KM, Upton CH, Viggiano L, Milosevic J, Raimondi G, McClain L, Chowdari K, Tischfield J, Sheldon M *et al*: **Persistent infection by HSV-1 is associated with changes in functional architecture of iPSC-derived neurons and brain activation patterns underlying working memory performance**. *Schizophrenia bulletin* 2015, **41**(1):123-132.

471. Daumann J, Schnitker R, Weidemann J, Schnell K, Thron A, Gouzoulis-Mayfrank E: **Neural correlates of working memory in pure and polyvalent ecstasy (MDMA) users**. *Neuroreport* 2003, **14**(15):1983-1987.

472. Daumann J, Jr., Fischermann T, Heekeren K, Thron A, Gouzoulis-Mayfrank E: **Neural mechanisms of working memory in ecstasy (MDMA) users who continue or discontinue ecstasy and amphetamine use: evidence from an 18-month longitudinal functional magnetic resonance imaging study**. *Biol Psychiatry* 2004, **56**(5):349-355.

473. de Frias CM, Marklund P, Eriksson E, Larsson A, Oman L, Annerbrink K, Backman L, Nilsson LG, Nyberg L: **Influence of COMT gene polymorphism on fMRI-assessed sustained and transient activity during a working memory task**. *Journal of cognitive neuroscience* 2010, **22**(7):1614-1622.

474. de Vries FE, de Wit SJ, Cath DC, van der Werf YD, van der Borden V, van Rossum TB, van Balkom AJ, van der Wee NJ, Veltman DJ, van den Heuvel OA: **Compensatory frontoparietal activity during working memory: an endophenotype of obsessive-compulsive disorder**. *Biol Psychiatry* 2014, **76**(11):878-887.

475. Dettwiler A, Murugavel M, Putukian M, Cubon V, Furtado J, Osherson D: **Persistent differences in patterns of brain activation after sports-related concussion: a longitudinal functional magnetic resonance imaging study**. *J Neurotrauma* 2014, **31**(2):180-188.

476. Di Giorgio A, Smith RM, Fazio L, D'Ambrosio E, Gelao B, Tomasicchio A, Selvaggi P, Taurisano P, Quarto T, Masellis R *et al*: **DRD2/CHRNA5 interaction on prefrontal biology and physiology during working memory**. *PloS one* 2014, **9**(5):e95997.

477. DiFrancesco MW, Holland SK, Ris MD, Adler CM, Nelson S, DelBello MP, Altaye M, Brunner HI: **Functional magnetic resonance imaging assessment of cognitive function in childhood-onset systemic lupus erythematosus: a pilot study**. *Arthritis and rheumatism* 2007, **56**(12):4151-4163.

478. Diwadkar VA, Pruitt P, Goradia D, Murphy E, Bakshi N, Keshavan MS, Rajan U, Reid A, Zajac-Benitez C: **Fronto-parietal hypo-activation during working memory independent of structural abnormalities: conjoint fMRI and sMRI analyses in adolescent offspring of schizophrenia patients**. *NeuroImage* 2011, **58**(1):234-241.

479. Diwadkar VA, Meintjes EM, Goradia D, Dodge NC, Warton C, Molteno CD, Jacobson SW, Jacobson JL: **Differences in cortico-striatal-cerebellar activation during working memory in syndromal and nonsyndromal children with prenatal alcohol exposure**. *Human brain mapping* 2013, **34**(8):1931-1945.

480. Dohnel K, Sommer M, Ibach B, Rothmayr C, Meinhardt J, Hajak G: **Neural correlates of emotional working memory in patients with mild cognitive impairment**. *Neuropsychologia* 2008, **46**(1):37-48.

481. Drummond SP, Walker M, Almklov E, Campos M, Anderson DE, Straus LD: **Neural correlates of working memory performance in primary insomnia**. *Sleep* 2013, **36**(9):1307-1316.

482. Druzgal TJ, D'Esposito M: **Activity in fusiform face area modulated as a function of working memory load**. *Brain research Cognitive brain research* 2001, **10**(3):355-364.

483. Dumas JA, Saykin AJ, McDonald BC, McAllister TW, Hynes ML, Newhouse PA: **Nicotinic versus muscarinic blockade alters verbal working memory-related brain activity in older women**. *The American journal of geriatric psychiatry : official journal of the American Association for Geriatric Psychiatry* 2008, **16**(4):272-282.

484. Dumas JA, Kutz AM, Naylor MR, Johnson JV, Newhouse PA: **Increased memory load-related frontal activation after estradiol treatment in postmenopausal women**. *Hormones and behavior* 2010, **58**(5):929-935.

485. Dumas JA, Kutz AM, McDonald BC, Naylor MR, Pfaff AC, Saykin AJ, Newhouse PA: **Increased working memory-related brain activity in middle-aged women with cognitive complaints**. *Neurobiology of aging* 2013, **34**(4):1145-1147.

486. Dumas JA, Newhouse PA: **Impaired working memory in geriatric depression: an FMRI study**. *The American journal of geriatric psychiatry : official journal of the American Association for Geriatric Psychiatry* 2015, **23**(4):433-436.

487. Elsabagh S, Premkumar P, Anilkumar AP, Kumari V: **A longer duration of schizophrenic illness has sex-specific associations within the working memory neural network in schizophrenia**. *Behavioural brain research* 2009, **201**(1):41-47.

488. Elzinga BM, Ardon AM, Heijnis MK, De Ruiter MB, Van Dyck R, Veltman DJ: **Neural correlates of enhanced working-memory performance in dissociative disorder: a functional MRI study**. *Psychol Med* 2007, **37**(2):235-245.

489. Epperson CN, Amin Z, Ruparel K, Gur R, Loughead J: **Interactive effects of estrogen and serotonin on brain activation during working memory and affective processing in menopausal women**. *Psychoneuroendocrinology* 2012, **37**(3):372-382.

490. Ernst T, Chang L, Jovicich J, Ames N, Arnold S: **Abnormal brain activation on functional MRI in cognitively asymptomatic HIV patients**. *Neurology* 2002, **59**(9):1343-1349.

491. Esposito F, Bertolino A, Scarabino T, Latorre V, Blasi G, Popolizio T, Tedeschi G, Cirillo S, Goebel R, Di Salle F: **Independent component model of the default-mode brain function: Assessing the impact of active thinking**. *Brain research bulletin* 2006, **70**(4-6):263-269.

492. Esposito F, Aragri A, Latorre V, Popolizio T, Scarabino T, Cirillo S, Marciano E, Tedeschi G, Di Salle F: **Does the default-mode functional connectivity of the brain correlate with working-memory performances?** *Archives italiennes de biologie* 2009, **147**(1-2):11-20.

493. Esposito F, Aragri A, Piccoli T, Tedeschi G, Goebel R, Di Salle F: **Distributed analysis of simultaneous EEG-fMRI time-series: modeling and interpretation issues**. *Magnetic resonance imaging* 2009, **27**(8):1120-1130.

494. Esslinger C, Schuler N, Sauer C, Gass D, Mier D, Braun U, Ochs E, Schulze TG, Rietschel M, Kirsch P *et al*: **Induction and quantification of prefrontal cortical network plasticity using 5 Hz rTMS and fMRI**. *Human brain mapping* 2014, **35**(1):140-151.

495. Ettinger U, Williams SC, Fannon D, Premkumar P, Kuipers E, Moller HJ, Kumari V: **Functional magnetic resonance imaging of a parametric working memory task in schizophrenia: relationship with performance and effects of antipsychotic treatment**. *Psychopharmacology* 2011, **216**(1):17-27.

496. Faget-Agius C, Boyer L, Lancon C, Richieri R, Fassio E, Soulier E, Chanoine V, Auquier P, Ranjeva JP, Guye M: **Structural and functional reorganization of working memory system during the first decade in schizophrenia. A cross-sectional study**. *Schizophr Res* 2013, **151**(1-3):48-60.

497. Falcone M, Wileyto EP, Ruparel K, Gerraty RT, LaPrate L, Detre JA, Gur R, Loughead J, Lerman C: **Age-related differences in working memory deficits during nicotine withdrawal**. *Addiction biology* 2014, **19**(5):907-917.

498. Fatjo-Vilas M, Pomarol-Clotet E, Salvador R, Monte GC, Gomar JJ, Sarro S, Ortiz-Gil J, Aguirre C, Landin-Romero R, Guerrero-Pedraza A *et al*: **Effect of the interleukin-1beta gene on dorsolateral prefrontal cortex function in schizophrenia: a genetic neuroimaging study**. *Biol Psychiatry* 2012, **72**(9):758-765.

499. Fernandez-Corcuera P, Salvador R, Monte GC, Salvador Sarro S, Goikolea JM, Amann B, Moro N, Sans-Sansa B, Ortiz-Gil J, Vieta E *et al*: **Bipolar depressed patients show both failure to activate and failure to de-activate during performance of a working memory task**. *Journal of affective disorders* 2013, **148**(2-3):170-178.

500. Fitzgerald PB, Srithiran A, Benitez J, Daskalakis ZZ, Oxley TJ, Kulkarni J, Egan GF: **An fMRI study of prefrontal brain activation during multiple tasks in patients with major depressive disorder**. *Human brain mapping* 2008, **29**(4):490-501.

501. Fitzgibbon BM, Fairhall SL, Kirk IJ, Kalev-Zylinska M, Pui K, Dalbeth N, Keelan S, Robinson E, During M, McQueen FM: **Functional MRI in NPSLE patients reveals increased parietal and frontal brain activation during a working memory task compared with controls**. *Rheumatology (Oxford, England)* 2008, **47**(1):50-53.

502. Forn C, Barros-Loscertales A, Escudero J, Benlloch V, Campos S, Antonia Parcet M, Avila C: **Compensatory activations in patients with multiple sclerosis during preserved performance on the auditory N-back task**. *Human brain mapping* 2007, **28**(5):424-430.

503. Frangou S, Kington J, Raymont V, Shergill SS: **Examining ventral and dorsal prefrontal function in bipolar disorder: a functional magnetic resonance imaging study**. *Eur Psychiatry* 2008, **23**(4):300-308.

504. Fusar-Poli P, Howes OD, Allen P, Broome M, Valli I, Asselin MC, Grasby PM, McGuire PK: **Abnormal frontostriatal interactions in people with prodromal signs of psychosis: a multimodal imaging study**. *Archives of general psychiatry* 2010, **67**(7):683-691.

505. Fusar-Poli P, Broome MR, Woolley JB, Johns LC, Tabraham P, Bramon E, Valmaggia L, Williams SC, McGuire P: **Altered brain function directly related to structural abnormalities in people at ultra high risk of psychosis: longitudinal VBM-fMRI study**. *J Psychiatr Res* 2011, **45**(2):190-198.

506. Gaudeau-Bosma C, Moulier V, Allard AC, Sidhoumi D, Bouaziz N, Braha S, Volle E, Januel D: **Effect of two weeks of rTMS on brain activity in healthy subjects during an n-back task: a randomized double blind study**. *Brain stimulation* 2013, **6**(4):569-575.

507. Geerligs L, Saliasi E, Renken RJ, Maurits NM, Lorist MM: **Flexible connectivity in the aging brain revealed by task modulations**. *Human brain mapping* 2014, **35**(8):3788-3804.

508. Gelao B, Fazio L, Selvaggi P, Di Giorgio A, Taurisano P, Quarto T, Romano R, Porcelli A, Mancini M, Masellis R *et al*: **DRD2 genotype predicts prefrontal activity during working memory after stimulation of D2 receptors with bromocriptine**. *Psychopharmacology* 2014, **231**(11):2361-2370.

509. George MS, Molnar CE, Grenesko EL, Anderson B, Mu Q, Johnson K, Nahas Z, Knable M, Fernandes P, Juncos J *et al*: **A single 20 mg dose of dihydrexidine (DAR-0100), a full dopamine D1 agonist, is safe and tolerated in patients with schizophrenia**. *Schizophr Res* 2007, **93**(1-3):42-50.

510. Georgiou-Karistianis N, Poudel GR, Dominguez DJ, Langmaid R, Gray MA, Churchyard A, Chua P, Borowsky B, Egan GF, Stout JC: **Functional and connectivity changes during working memory in Huntington's disease: 18 month longitudinal data from the IMAGE-HD study**. *Brain and cognition* 2013, **83**(1):80-91.

511. Georgiou-Karistianis N, Stout JC, Dominguez DJ, Carron SP, Ando A, Churchyard A, Chua P, Bohanna I, Dymowski AR, Poudel G *et al*: **Functional magnetic resonance imaging of working memory in Huntington's disease: cross-sectional data from the IMAGE-HD study**. *Human brain mapping* 2014, **35**(5):1847-1864.

512. Ginestet CE, Simmons A: **Statistical parametric network analysis of functional connectivity dynamics during a working memory task**. *NeuroImage* 2011, **55**(2):688-704.

513. Gonzales MM, Tarumi T, Miles SC, Tanaka H, Shah F, Haley AP: **Insulin sensitivity as a mediator of the relationship between BMI and working memory-related brain activation**. *Obesity (Silver Spring, Md)* 2010, **18**(11):2131-2137.

514. Gonzales MM, Tarumi T, Tanaka H, Sugawara J, Swann-Sternberg T, Goudarzi K, Haley AP: **Functional imaging of working memory and peripheral endothelial function in middle-aged adults**. *Brain and cognition* 2010, **73**(2):146-151.

515. Gonzales MM, Tarumi T, Eagan DE, Tanaka H, Biney FO, Haley AP: **Current serum lipoprotein levels and FMRI response to working memory in midlife**. *Dementia and geriatric cognitive disorders* 2011, **31**(4):259-267.

516. Gonzales MM, Kaur S, Eagan DE, Goudarzi K, Pasha E, Doan DC, Tanaka H, Haley AP: **Central adiposity and the functional magnetic resonance imaging response to cognitive challenge**. *International journal of obesity (2005)* 2014, **38**(9):1193-1199.

517. Gonzales MM, Tarumi T, Mumford JA, Ellis RC, Hungate JR, Pyron M, Tanaka H, Haley AP: **Greater BOLD response to working memory in endurance-trained adults revealed by breath-hold calibration**. *Human brain mapping* 2014, **35**(7):2898-2910.

518. Gordon EM, Breeden AL, Bean SE, Vaidya CJ: **Working memory-related changes in functional connectivity persist beyond task disengagement**. *Human brain mapping* 2014, **35**(3):1004-1017.

519. Gordon EM, Devaney JM, Bean S, Vaidya CJ: **Resting-state striato-frontal functional connectivity is sensitive to DAT1 genotype and predicts executive function**. *Cerebral cortex (New York, NY : 1991)* 2015, **25**(2):336-345.

520. Greve DN, Duntley SP, Larson-Prior L, Krystal AD, Diaz MT, Drummond SP, Thein SG, Kushida CA, Yang R, Thomas RJ: **Effect of armodafinil on cortical activity and working memory in patients with residual excessive sleepiness associated with CPAP-Treated OSA: a multicenter fMRI study**. *Journal of clinical sleep medicine : JCSM : official publication of the American Academy of Sleep Medicine* 2014, **10**(2):143-153.

521. Griebe M, Amann M, Hirsch JG, Achtnichts L, Hennerici MG, Gass A, Szabo K: **Reduced functional reserve in patients with age-related white matter changes: a preliminary FMRI study of working memory**. *PloS one* 2014, **9**(8):e103359.

522. Griffiths ST, Gundersen H, Neto E, Elgen I, Markestad T, Aukland SM, Hugdahl K: **fMRI: blood oxygen level-dependent activation during a working memory-selective attention task in children born extremely preterm**. *Pediatric research* 2013, **74**(2):196-205.

523. Gropman AL, Shattuck K, Prust MJ, Seltzer RR, Breeden AL, Hailu A, Rigas A, Hussain R, VanMeter J: **Altered neural activation in ornithine transcarbamylase deficiency during executive cognition: an fMRI study**. *Human brain mapping* 2013, **34**(4):753-761.

524. Guerrero-Pedraza A, McKenna PJ, Gomar JJ, Sarro S, Salvador R, Amann B, Carrion MI, Landin-Romero R, Blanch J, Pomarol-Clotet E: **First-episode psychosis is characterized by failure of deactivation but not by hypo- or hyperfrontality**. *Psychol Med* 2012, **42**(1):73-84.

525. Guse B, Falkai P, Gruber O, Whalley H, Gibson L, Hasan A, Obst K, Dechent P, McIntosh A, Suchan B *et al*: **The effect of long-term high frequency repetitive transcranial magnetic stimulation on working memory in schizophrenia and healthy controls--a randomized placebo-controlled, double-blind fMRI study**. *Behavioural brain research* 2013, **237**:300-307.

526. Haberecht MF, Menon V, Warsofsky IS, White CD, Dyer-Friedman J, Glover GH, Neely EK, Reiss AL: **Functional neuroanatomy of visuo-spatial working memory in Turner syndrome**. *Human brain mapping* 2001, **14**(2):96-107.

527. Haeussinger FB, Dresler T, Heinzel S, Schecklmann M, Fallgatter AJ, Ehlis AC: **Reconstructing functional near-infrared spectroscopy (fNIRS) signals impaired by extra-cranial confounds: an easy-to-use filter method**. *NeuroImage* 2014, **95**:69-79.

528. Haldane M, Jogia J, Cobb A, Kozuch E, Kumari V, Frangou S: **Changes in brain activation during working memory and facial recognition tasks in patients with bipolar disorder with Lamotrigine monotherapy**. *European neuropsychopharmacology : the journal of the European College of Neuropsychopharmacology* 2008, **18**(1):48-54.

529. Haley AP, Sweet LH, Gunstad J, Forman DE, Poppas A, Paul RH, Tate DF, Cohen RA: **Verbal working memory and atherosclerosis in patients with cardiovascular disease: an fMRI study**. *Journal of neuroimaging : official journal of the American Society of Neuroimaging* 2007, **17**(3):227-233.

530. Haller S, Rodriguez C, Moser D, Toma S, Hofmeister J, Sinanaj I, Van De Ville D, Giannakopoulos P, Lovblad KO: **Acute caffeine administration impact on working memory-related brain activation and functional connectivity in the elderly: a BOLD and perfusion MRI study**. *Neuroscience* 2013, **250**:364-371.

531. Haller S, Montandon ML, Rodriguez C, Moser D, Toma S, Hofmeister J, Sinanaj I, Lovblad KO, Giannakopoulos P: **Acute caffeine administration effect on brain activation patterns in mild cognitive impairment**. *Journal of Alzheimer's disease : JAD* 2014, **41**(1):101-112.

532. Harding IH, Yucel M, Harrison BJ, Pantelis C, Breakspear M: **Effective connectivity within the frontoparietal control network differentiates cognitive control and working memory**. *NeuroImage* 2015, **106**:144-153.

533. Harvey PO, Van den Eynde F, Zangen A, Berlim MT: **Neural correlates of clinical improvement after deep transcranial magnetic stimulation (DTMS) for treatment-resistant depression: a case report using functional magnetic resonance imaging**. *Neurocase* 2015, **21**(1):16-22.

534. Hautzel H, Mottaghy FM, Specht K, Muller HW, Krause BJ: **Evidence of a modality-dependent role of the cerebellum in working memory? An fMRI study comparing verbal and abstract n-back tasks**. *NeuroImage* 2009, **47**(4):2073-2082.

535. Havermans A, Vuurman EF, van den Hurk J, Hoogsteder P, van Schayck OC: **Treatment with a nicotine vaccine does not lead to changes in brain activity during smoking cue exposure or a working memory task**. *Addiction (Abingdon, England)* 2014, **109**(8):1260-1267.

536. He XS, Wang ZX, Zhu YZ, Wang N, Hu X, Zhang DR, Zhu DF, Zhou JN: **Hyperactivation of working memory-related brain circuits in newly diagnosed middle-aged type 2 diabetics**. *Acta diabetologica* 2015, **52**(1):133-142.

537. Heinzel S, Lorenz RC, Brockhaus WR, Wustenberg T, Kathmann N, Heinz A, Rapp MA: **Working memory load-dependent brain response predicts behavioral training gains in older adults**. *The Journal of neuroscience : the official journal of the Society for Neuroscience* 2014, **34**(4):1224-1233.

538. Henckens MJ, van Wingen GA, Joels M, Fernandez G: **Time-dependent corticosteroid modulation of prefrontal working memory processing**. *Proceedings of the National Academy of Sciences of the United States of America* 2011, **108**(14):5801-5806.

539. Hirano Y, Obata T, Kashikura K, Nonaka H, Tachibana A, Ikehira H, Onozuka M: **Effects of chewing in working memory processing**. *Neurosci Lett* 2008, **436**(2):189-192.

540. Honey GD, Fu CH, Kim J, Brammer MJ, Croudace TJ, Suckling J, Pich EM, Williams SC, Bullmore ET: **Effects of verbal working memory load on corticocortical connectivity modeled by path analysis of functional magnetic resonance imaging data**. *NeuroImage* 2002, **17**(2):573-582.

541. Honey GD, Bullmore ET, Sharma T: **De-coupling of cognitive performance and cerebral functional response during working memory in schizophrenia**. *Schizophr Res* 2002, **53**(1-2):45-56.

542. Hoth KF, Gonzales MM, Tarumi T, Miles SC, Tanaka H, Haley AP: **Functional MR imaging evidence of altered functional activation in metabolic syndrome**. *AJNR American journal of neuroradiology* 2011, **32**(3):541-547.

543. Hu Y, Chen X, Gu H, Yang Y: **Resting-state glutamate and GABA concentrations predict task-induced deactivation in the default mode network**. *The Journal of neuroscience : the official journal of the Society for Neuroscience* 2013, **33**(47):18566-18573.

544. Hurt H, Giannetta JM, Korczykowski M, Hoang A, Tang KZ, Betancourt L, Brodsky NL, Shera DM, Farah MJ, Detre JA: **Functional magnetic resonance imaging and working memory in adolescents with gestational cocaine exposure**. *The Journal of pediatrics* 2008, **152**(3):371-377.

545. Ikeda T, Osaka N: **How are colors memorized in working memory? A functional magnetic resonance imaging study**. *Neuroreport* 2007, **18**(2):111-114.

546. Jacobsen LK, D'Souza DC, Mencl WE, Pugh KR, Skudlarski P, Krystal JH: **Nicotine effects on brain function and functional connectivity in schizophrenia**. *Biol Psychiatry* 2004, **55**(8):850-858.

547. Jacola LM, Willard VW, Ashford JM, Ogg RJ, Scoggins MA, Jones MM, Wu S, Conklin HM: **Clinical utility of the N-back task in functional neuroimaging studies of working memory**. *Journal of clinical and experimental neuropsychology* 2014, **36**(8):875-886.

548. Jansen A, Krach S, Krug A, Markov V, Eggermann T, Zerres K, Stocker T, Shah NJ, Nothen MM, Treutlein J *et al*: **A putative high risk diplotype of the G72 gene is in healthy individuals associated with better performance in working memory functions and altered brain activity in the medial temporal lobe**. *NeuroImage* 2009, **45**(3):1002-1008.

549. Jansma JM, Ramsey NF, Coppola R, Kahn RS: **Specific versus nonspecific brain activity in a parametric N-back task**. *NeuroImage* 2000, **12**(6):688-697.

550. Kalmady SV, Agarwal SM, Shivakumar V, Jose D, Venkatasubramanian G, Reddy YC: **Revisiting Geschwind's hypothesis on brain lateralisation: a functional MRI study of digit ratio (2D:4D) and sex interaction effects on spatial working memory**. *Laterality* 2013, **18**(5):625-640.

551. Kalpakidou AK, Allin MP, Walshe M, Giampietro V, McGuire PK, Rifkin L, Murray RM, Nosarti C: **Functional neuroanatomy of executive function after neonatal brain injury in adults who were born very preterm**. *PloS one* 2014, **9**(12):e113975.

552. Karch S, Leicht G, Giegling I, Lutz J, Kunz J, Buselmeier M, Hey P, Sporl A, Jager L, Meindl T *et al*: **Inefficient neural activity in patients with schizophrenia and nonpsychotic relatives of schizophrenic patients: evidence from a working memory task**. *J Psychiatr Res* 2009, **43**(15):1185-1194.

553. Kasahara M, Menon DK, Salmond CH, Outtrim JG, Tavares JV, Carpenter TA, Pickard JD, Sahakian BJ, Stamatakis EA: **Traumatic brain injury alters the functional brain network mediating working memory**. *Brain injury* 2011, **25**(12):1170-1187.

554. Kearney-Ramos TE, Fausett JS, Gess JL, Reno A, Peraza J, Kilts CD, James GA: **Merging clinical neuropsychology and functional neuroimaging to evaluate the construct validity and neural network engagement of the n-back task**. *Journal of the International Neuropsychological Society : JINS* 2014, **20**(7):736-750.

555. Kerestes R, Ladouceur CD, Meda S, Nathan PJ, Blumberg HP, Maloney K, Ruf B, Saricicek A, Pearlson GD, Bhagwagar Z *et al*: **Abnormal prefrontal activity subserving attentional control of emotion in remitted depressed patients during a working memory task with emotional distracters**. *Psychol Med* 2012, **42**(1):29-40.

556. Kim J, Whyte J, Wang J, Rao H, Tang KZ, Detre JA: **Continuous ASL perfusion fMRI investigation of higher cognition: quantification of tonic CBF changes during sustained attention and working memory tasks**. *NeuroImage* 2006, **31**(1):376-385.

557. Kim J, Whyte J, Patel S, Europa E, Slattery J, Coslett HB, Detre JA: **A perfusion fMRI study of the neural correlates of sustained-attention and working-memory deficits in chronic traumatic brain injury**. *Neurorehabilitation and neural repair* 2012, **26**(7):870-880.

558. Kim C, Johnson NF, Gold BT: **Conflict adaptation in prefrontal cortex: now you see it, now you don't**. *Cortex; a journal devoted to the study of the nervous system and behavior* 2014, **50**:76-85.

559. Klemen J, Buchel C, Buhler M, Menz MM, Rose M: **Auditory working memory load impairs visual ventral stream processing: toward a unified model of attentional load**. *Journal of cognitive neuroscience* 2010, **22**(3):437-446.

560. Knops A, Nuerk HC, Fimm B, Vohn R, Willmes K: **A special role for numbers in working memory? An fMRI study**. *NeuroImage* 2006, **29**(1):1-14.

561. Ko CH, Yen JY, Yen CF, Chen CS, Lin WC, Wang PW, Liu GC: **Brain activation deficit in increased-load working memory tasks among adults with ADHD using fMRI**. *Eur Arch Psychiatry Clin Neurosci* 2013, **263**(7):561-573.

562. Kobel M, Bechtel N, Weber P, Specht K, Klarhofer M, Scheffler K, Opwis K, Penner IK: **Effects of methylphenidate on working memory functioning in children with attention deficit/hyperactivity disorder**. *European journal of paediatric neurology : EJPN : official journal of the European Paediatric Neurology Society* 2009, **13**(6):516-523.

563. Koch K, Pauly K, Kellermann T, Seiferth NY, Reske M, Backes V, Stocker T, Shah NJ, Amunts K, Kircher T *et al*: **Gender differences in the cognitive control of emotion: An fMRI study**. *Neuropsychologia* 2007, **45**(12):2744-2754.

564. Koch K, Wagner G, Schachtzabel C, Peikert G, Schultz CC, Sauer H, Schlosser RG: **Aberrant anterior cingulate activation in obsessive-compulsive disorder is related to task complexity**. *Neuropsychologia* 2012, **50**(5):958-964.

565. Kodama K, Terao T, Hatano K, Kohno K, Makino M, Mizokami Y, Kamei K, Katayama Y, Hoaki Y, Sakai A *et al*: **Identification of the neural correlates of cyclothymic temperament using a working memory task in fMRI**. *Journal of affective disorders* 2015, **171**:1-5.

566. Koike S, Takizawa R, Nishimura Y, Kinou M, Kawasaki S, Kasai K: **Reduced but broader prefrontal activity in patients with schizophrenia during n-back working memory tasks: a multi-channel near-infrared spectroscopy study**. *J Psychiatr Res* 2013, **47**(9):1240-1246.

567. Koppe G, Gruppe H, Sammer G, Gallhofer B, Kirsch P, Lis S: **Temporal unpredictability of a stimulus sequence affects brain activation differently depending on cognitive task demands**. *NeuroImage* 2014, **101**:236-244.

568. Korsnes MS, Lovdahl H, Andersson S, Bjornerud A, Due-Tonnesen P, Endestad T, Malt UF: **Working memory in recurrent brief depression: an fMRI pilot study**. *Journal of affective disorders* 2013, **149**(1-3):383-392.

569. Koshino H, Carpenter PA, Minshew NJ, Cherkassky VL, Keller TA, Just MA: **Functional connectivity in an fMRI working memory task in high-functioning autism**. *NeuroImage* 2005, **24**(3):810-821.

570. Krug A, Markov V, Eggermann T, Krach S, Zerres K, Stocker T, Shah NJ, Schneider F, Nothen MM, Treutlein J *et al*: **Genetic variation in the schizophrenia-risk gene neuregulin1 correlates with differences in frontal brain activation in a working memory task in healthy individuals**. *NeuroImage* 2008, **42**(4):1569-1576.

571. Kumari V, Gray JA, ffytche DH, Mitterschiffthaler MT, Das M, Zachariah E, Vythelingum GN, Williams SC, Simmons A, Sharma T: **Cognitive effects of nicotine in humans: an fMRI study**. *NeuroImage* 2003, **19**(3):1002-1013.

572. Kumari V, Aasen I, ffytche D, Williams SC, Sharma T: **Neural correlates of adjunctive rivastigmine treatment to antipsychotics in schizophrenia: a randomized, placebo-controlled, double-blind fMRI study**. *NeuroImage* 2006, **29**(2):545-556.

573. Kumari V, Aasen I, Taylor P, Ffytche DH, Das M, Barkataki I, Goswami S, O'Connell P, Howlett M, Williams SC *et al*: **Neural dysfunction and violence in schizophrenia: an fMRI investigation**. *Schizophr Res* 2006, **84**(1):144-164.

574. Kumari V, Peters ER, Fannon D, Antonova E, Premkumar P, Anilkumar AP, Williams SC, Kuipers E: **Dorsolateral prefrontal cortex activity predicts responsiveness to cognitive-behavioral therapy in schizophrenia**. *Biol Psychiatry* 2009, **66**(6):594-602.

575. Kwon H, Menon V, Eliez S, Warsofsky IS, White CD, Dyer-Friedman J, Taylor AK, Glover GH, Reiss AL: **Functional neuroanatomy of visuospatial working memory in fragile X syndrome: relation to behavioral and molecular measures**. *Am J Psychiatry* 2001, **158**(7):1040-1051.

576. Kwon H, Reiss AL, Menon V: **Neural basis of protracted developmental changes in visuo-spatial working memory**. *Proceedings of the National Academy of Sciences of the United States of America* 2002, **99**(20):13336-13341.

577. Landin-Romero R, Novo P, Vicens V, McKenna PJ, Santed A, Pomarol-Clotet E, Salgado-Pineda P, Shapiro F, Amann BL: **EMDR therapy modulates the default mode network in a subsyndromal, traumatized bipolar patient**. *Neuropsychobiology* 2013, **67**(3):181-184.

578. Landin-Romero R, McKenna PJ, Salgado-Pineda P, Sarro S, Aguirre C, Sarri C, Compte A, Bosque C, Blanch J, Salvador R *et al*: **Failure of deactivation in the default mode network: a trait marker for schizophrenia?** *Psychol Med* 2015, **45**(6):1315-1325.

579. Lao-Kaim NP, Giampietro VP, Williams SC, Simmons A, Tchanturia K: **Functional MRI investigation of verbal working memory in adults with anorexia nervosa**. *Eur Psychiatry* 2014, **29**(4):211-218.

580. Lee TW, Liu HL, Wai YY, Ko HJ, Lee SH: **Abnormal neural activity in partially remitted late-onset depression: an fMRI study of one-back working memory task**. *Psychiatry research* 2013, **213**(2):133-141.

581. Lenartowicz A, McIntosh AR: **The role of anterior cingulate cortex in working memory is shaped by functional connectivity**. *Journal of cognitive neuroscience* 2005, **17**(7):1026-1042.

582. Leung AW, Alain C: **Working memory load modulates the auditory "What" and "Where" neural networks**. *NeuroImage* 2011, **55**(3):1260-1269.

583. Li T, Luo Q, Gong H: **Gender-specific hemodynamics in prefrontal cortex during a verbal working memory task by near-infrared spectroscopy**. *Behavioural brain research* 2010, **209**(1):148-153.

584. Li J, Chen C, Lei X, Wang Y, Chen C, He Q, Moyzis RK, Xue G, Zhu B, Cao Z *et al*: **The NTSR1 gene modulates the association between hippocampal structure and working memory performance**. *NeuroImage* 2013, **75**:79-86.

585. Li L, Men WW, Chang YK, Fan MX, Ji L, Wei GX: **Acute aerobic exercise increases cortical activity during working memory: a functional MRI study in female college students**. *PloS one* 2014, **9**(6):e99222.

586. Li Y, Li F, He N, Guo L, Huang X, Lui S, Gong Q: **Neural hyperactivity related to working memory in drug-naive boys with attention deficit hyperactivity disorder**. *Progress in neuro-psychopharmacology & biological psychiatry* 2014, **53**:116-122.

587. Liang X, Zou Q, He Y, Yang Y: **Coupling of functional connectivity and regional cerebral blood flow reveals a physiological basis for network hubs of the human brain**. *Proceedings of the National Academy of Sciences of the United States of America* 2013, **110**(5):1929-1934.

588. Liao LM, Zhou LX, Le HB, Yin JJ, Ma SH: **Spatial working memory dysfunction in minimal hepatic encephalopathy: an ethology and BOLD-fMRI study**. *Brain research* 2012, **1445**:62-72.

589. Libertus ME, Brannon EM, Pelphrey KA: **Developmental changes in category-specific brain responses to numbers and letters in a working memory task**. *NeuroImage* 2009, **44**(4):1404-1414.

590. C AL, P AF, Cameron I, A MS: **The long-term effects of prenatal nicotine exposure on verbal working memory: an fMRI study of young adults**. *Drug Alcohol Depend* 2014, **144**:61-69.

591. Loughead J, Wileyto EP, Valdez JN, Sanborn P, Tang K, Strasser AA, Ruparel K, Ray R, Gur RC, Lerman C: **Effect of abstinence challenge on brain function and cognition in smokers differs by COMT genotype**. *Mol Psychiatry* 2009, **14**(8):820-826.

592. Loughead J, Ray R, Wileyto EP, Ruparel K, Sanborn P, Siegel S, Gur RC, Lerman C: **Effects of the alpha4beta2 partial agonist varenicline on brain activity and working memory in abstinent smokers**. *Biol Psychiatry* 2010, **67**(8):715-721.

593. Loughead J, Wileyto EP, Ruparel K, Falcone M, Hopson R, Gur R, Lerman C: **Working memory-related neural activity predicts future smoking relapse**. *Neuropsychopharmacology : official publication of the American College of Neuropsychopharmacology* 2015, **40**(6):1311-1320.

594. Luckhoo H, Hale JR, Stokes MG, Nobre AC, Morris PG, Brookes MJ, Woolrich MW: **Inferring task-related networks using independent component analysis in magnetoencephalography**. *NeuroImage* 2012, **62**(1):530-541.

595. Ludwig C, Chicherio C, Terraneo L, Magistretti P, de Ribaupierre A, Slosman D: **Functional imaging studies of cognition using 99mTc-HMPAO SPECT: empirical validation using the n-back working memory paradigm**. *European journal of nuclear medicine and molecular imaging* 2008, **35**(4):695-703.

596. Luo Y, Qin S, Fernandez G, Zhang Y, Klumpers F, Li H: **Emotion perception and executive control interact in the salience network during emotionally charged working memory processing**. *Human brain mapping* 2014, **35**(11):5606-5616.

597. Lv ZX, Huang DH, Ye W, Chen ZR, Huang WL, Zheng JO: **Alteration of functional connectivity within visuospatial working memory-related brain network in patients with right temporal lobe epilepsy: a resting-state fMRI study**. *Epilepsy & behavior : E&B* 2014, **35**:64-71.

598. Madre M, Radua J, Landin-Romero R, Alonso-Lana S, Salvador R, Panicali F, Pomarol-Clotet E, Amann BL: **Trait or state? A longitudinal neuropsychological evaluation and fMRI study in schizoaffective disorder**. *Schizophr Res* 2014, **159**(2-3):458-464.

599. Manelis A, Reder LM: **He who is well prepared has half won the battle: an FMRI study of task preparation**. *Cerebral cortex (New York, NY : 1991)* 2015, **25**(3):726-735.

600. Mannie ZN, Harmer CJ, Cowen PJ, Norbury R: **A functional magnetic resonance imaging study of verbal working memory in young people at increased familial risk of depression**. *Biol Psychiatry* 2010, **67**(5):471-477.

601. Marklund P, Larsson A, Elgh E, Linder J, Riklund KA, Forsgren L, Nyberg L: **Temporal dynamics of basal ganglia under-recruitment in Parkinson's disease: transient caudate abnormalities during updating of working memory**. *Brain* 2009, **132**(Pt 2):336-346.

602. Markov V, Krug A, Krach S, Jansen A, Eggermann T, Zerres K, Stocker T, Shah NJ, Nothen MM, Treutlein J *et al*: **Impact of schizophrenia-risk gene dysbindin 1 on brain activation in bilateral middle frontal gyrus during a working memory task in healthy individuals**. *Human brain mapping* 2010, **31**(2):266-275.

603. Marquand AF, Mourao-Miranda J, Brammer MJ, Cleare AJ, Fu CH: **Neuroanatomy of verbal working memory as a diagnostic biomarker for depression**. *Neuroreport* 2008, **19**(15):1507-1511.

604. Marrelec G, Fransson P: **Assessing the influence of different ROI selection strategies on functional connectivity analyses of fMRI data acquired during steady-state conditions**. *PloS one* 2011, **6**(4):e14788.

605. Martinkauppi S, Rama P, Aronen HJ, Korvenoja A, Carlson S: **Working memory of auditory localization**. *Cerebral cortex (New York, NY : 1991)* 2000, **10**(9):889-898.

606. Massat I, Slama H, Kavec M, Linotte S, Mary A, Baleriaux D, Metens T, Mendlewicz J, Peigneux P: **Working memory-related functional brain patterns in never medicated children with ADHD**. *PloS one* 2012, **7**(11):e49392.

607. Matsuo K, Glahn DC, Peluso MA, Hatch JP, Monkul ES, Najt P, Sanches M, Zamarripa F, Li J, Lancaster JL *et al*: **Prefrontal hyperactivation during working memory task in untreated individuals with major depressive disorder**. *Mol Psychiatry* 2007, **12**(2):158-166.

608. Mattay VS, Fera F, Tessitore A, Hariri AR, Berman KF, Das S, Meyer-Lindenberg A, Goldberg TE, Callicott JH, Weinberger DR: **Neurophysiological correlates of age-related changes in working memory capacity**. *Neurosci Lett* 2006, **392**(1-2):32-37.

609. McAllister TW, Sparling MB, Flashman LA, Guerin SJ, Mamourian AC, Saykin AJ: **Differential working memory load effects after mild traumatic brain injury**. *NeuroImage* 2001, **14**(5):1004-1012.

610. McDonald BC, Conroy SK, Ahles TA, West JD, Saykin AJ: **Alterations in brain activation during working memory processing associated with breast cancer and treatment: a prospective functional magnetic resonance imaging study**. *Journal of clinical oncology : official journal of the American Society of Clinical Oncology* 2012, **30**(20):2500-2508.

611. McGeown WJ, Shanks MF, Forbes-McKay KE, Waiter GD, Elrick I, Venneri MG, Venneri A: **Established donepezil treatment modulates task relevant regional brain activation in early Alzheimer's disease**. *Current Alzheimer research* 2010, **7**(5):415-427.

612. Medaglia JD, Chiou KS, Slocomb J, Fitzpatrick NM, Wardecker BM, Ramanathan D, Vesek J, Good DC, Hillary FG: **The less BOLD, the wiser: support for the latent resource hypothesis after traumatic brain injury**. *Human brain mapping* 2012, **33**(4):979-993.

613. Meisenzahl EM, Scheuerecker J, Zipse M, Ufer S, Wiesmann M, Frodl T, Koutsouleris N, Zetzsche T, Schmitt G, Riedel M *et al*: **Effects of treatment with the atypical neuroleptic quetiapine on working memory function: a functional MRI follow-up investigation**. *Eur Arch Psychiatry Clin Neurosci* 2006, **256**(8):522-531.

614. Menzies L, Ooi C, Kamath S, Suckling J, McKenna P, Fletcher P, Bullmore E, Stephenson C: **Effects of gamma-aminobutyric acid-modulating drugs on working memory and brain function in patients with schizophrenia**. *Archives of general psychiatry* 2007, **64**(2):156-167.

615. Meusel LA, Hall GB, Fougere P, McKinnon MC, MacQueen GM: **Neural correlates of cognitive remediation in patients with mood disorders**. *Psychiatry research* 2013, **214**(2):142-152.

616. Migo EM, Mitterschiffthaler M, O'Daly O, Dawson GR, Dourish CT, Craig KJ, Simmons A, Wilcock GK, McCulloch E, Jackson SH *et al*: **Alterations in working memory networks in amnestic mild cognitive impairment**. *Neuropsychology, development, and cognition Section B, Aging, neuropsychology and cognition* 2015, **22**(1):106-127.

617. Miskowiak K, Inkster B, O'Sullivan U, Selvaraj S, Goodwin GM, Harmer CJ: **Differential effects of erythropoietin on neural and cognitive measures of executive function 3 and 7 days post-administration**. *Experimental brain research* 2008, **184**(3):313-321.

618. Mitchell RL: **fMRI delineation of working memory for emotional prosody in the brain: commonalities with the lexico-semantic emotion network**. *NeuroImage* 2007, **36**(3):1015-1025.

619. Nagel IE, Preuschhof C, Li SC, Nyberg L, Backman L, Lindenberger U, Heekeren HR: **Load modulation of BOLD response and connectivity predicts working memory performance in younger and older adults**. *Journal of cognitive neuroscience* 2011, **23**(8):2030-2045.

620. Nagel BJ, Herting MM, Maxwell EC, Bruno R, Fair D: **Hemispheric lateralization of verbal and spatial working memory during adolescence**. *Brain and cognition* 2013, **82**(1):58-68.

621. Nakao T, Nakagawa A, Nakatani E, Nabeyama M, Sanematsu H, Yoshiura T, Togao O, Tomita M, Masuda Y, Yoshioka K *et al*: **Working memory dysfunction in obsessive-compulsive disorder: a neuropsychological and functional MRI study**. *J Psychiatr Res* 2009, **43**(8):784-791.

622. Nejad AB, Ebdrup BH, Siebner HR, Rasmussen H, Aggernaes B, Glenthoj BY, Baare WF: **Impaired temporoparietal deactivation with working memory load in antipsychotic-naive patients with first-episode schizophrenia**. *The world journal of biological psychiatry : the official journal of the World Federation of Societies of Biological Psychiatry* 2011, **12**(4):271-281.

623. Nejad AB, Madsen KH, Ebdrup BH, Siebner HR, Rasmussen H, Aggernaes B, Glenthoj BY, Baare WF: **Neural markers of negative symptom outcomes in distributed working memory brain activity of antipsychotic-naive schizophrenia patients**. *The international journal of neuropsychopharmacology* 2013, **16**(6):1195-1204.

624. Newsome MR, Scheibel RS, Hunter JV, Wang ZJ, Chu Z, Li X, Levin HS: **Brain activation during working memory after traumatic brain injury in children**. *Neurocase* 2007, **13**(1):16-24.

625. Newsome MR, Scheibel RS, Steinberg JL, Troyanskaya M, Sharma RG, Rauch RA, Li X, Levin HS: **Working memory brain activation following severe traumatic brain injury**. *Cortex; a journal devoted to the study of the nervous system and behavior* 2007, **43**(1):95-111.

626. Newton AT, Morgan VL, Rogers BP, Gore JC: **Modulation of steady state functional connectivity in the default mode and working memory networks by cognitive load**. *Human brain mapping* 2011, **32**(10):1649-1659.

627. Nichols TT, Gates KM, Molenaar PC, Wilson SJ: **Greater BOLD activity but more efficient connectivity is associated with better cognitive performance within a sample of nicotine-deprived smokers**. *Addiction biology* 2014, **19**(5):931-940.

628. Nixon DC, Prust MJ, Sambataro F, Tan HY, Mattay VS, Weinberger DR, Callicott JH: **Interactive effects of DAOA (G72) and catechol-O-methyltransferase on neurophysiology in prefrontal cortex**. *Biol Psychiatry* 2011, **69**(10):1006-1008.

629. Norbury R, Godlewska B, Cowen PJ: **When less is more: a functional magnetic resonance imaging study of verbal working memory in remitted depressed patients**. *Psychol Med* 2014, **44**(6):1197-1203.

630. Nyberg L, Dahlin E, Stigsdotter Neely A, Backman L: **Neural correlates of variable working memory load across adult age and skill: dissociative patterns within the fronto-parietal network**. *Scand J Psychol* 2009, **50**(1):41-46.

631. Nystrom LE, Braver TS, Sabb FW, Delgado MR, Noll DC, Cohen JD: **Working memory for letters, shapes, and locations: fMRI evidence against stimulus-based regional organization in human prefrontal cortex**. *NeuroImage* 2000, **11**(5 Pt 1):424-446.

632. Oakes TR, Johnstone T, Ores Walsh KS, Greischar LL, Alexander AL, Fox AS, Davidson RJ: **Comparison of fMRI motion correction software tools**. *NeuroImage* 2005, **28**(3):529-543.

633. O'Daly OG, Joyce D, Stephan KE, Murray RM, Shergill SS: **Functional magnetic resonance imaging investigation of the amphetamine sensitization model of schizophrenia in healthy male volunteers**. *Archives of general psychiatry* 2011, **68**(6):545-554.

634. Oksanen KM, Waldum ER, McDaniel MA, Braver TS: **Neural mechanisms of time-based prospective memory: evidence for transient monitoring**. *PloS one* 2014, **9**(3):e92123.

635. Ortiz-Gil J, Pomarol-Clotet E, Salvador R, Canales-Rodriguez EJ, Sarro S, Gomar JJ, Guerrero A, Sans-Sansa B, Capdevila A, Junque C *et al*: **Neural correlates of cognitive impairment in schizophrenia**. *The British journal of psychiatry : the journal of mental science* 2011, **199**(3):202-210.

636. Palacios EM, Sala-Llonch R, Junque C, Roig T, Tormos JM, Bargallo N, Vendrell P: **White matter integrity related to functional working memory networks in traumatic brain injury**. *Neurology* 2012, **78**(12):852-860.

637. Papassotiropoulos A, Henke K, Stefanova E, Aerni A, Muller A, Demougin P, Vogler C, Sigmund JC, Gschwind L, Huynh KD *et al*: **A genome-wide survey of human short-term memory**. *Mol Psychiatry* 2011, **16**(2):184-192.

638. Park HJ, Chun JW, Park B, Park H, Kim JI, Lee JD, Kim JJ: **Activation of the occipital cortex and deactivation of the default mode network during working memory in the early blind**. *Journal of the International Neuropsychological Society : JINS* 2011, **17**(3):407-422.

639. Paulus FM, Bedenbender J, Krach S, Pyka M, Krug A, Sommer J, Mette M, Nothen MM, Witt SH, Rietschel M *et al*: **Association of rs1006737 in CACNA1C with alterations in prefrontal activation and fronto-hippocampal connectivity**. *Human brain mapping* 2014, **35**(4):1190-1200.

640. Pauly K, Seiferth NY, Kellermann T, Backes V, Vloet TD, Shah NJ, Schneider F, Habel U, Kircher TT: **Cerebral dysfunctions of emotion-cognition interactions in adolescent-onset schizophrenia**. *Journal of the American Academy of Child and Adolescent Psychiatry* 2008, **47**(11):1299-1310.

641. Pauly K, Seiferth NY, Kellermann T, Ruhrmann S, Daumann B, Backes V, Klosterkotter J, Shah NJ, Schneider F, Kircher TT *et al*: **The interaction of working memory and emotion in persons clinically at risk for psychosis: an fMRI pilot study**. *Schizophr Res* 2010, **120**(1-3):167-176.

642. Pavisian B, MacIntosh BJ, Szilagyi G, Staines RW, O'Connor P, Feinstein A: **Effects of cannabis on cognition in patients with MS: a psychometric and MRI study**. *Neurology* 2014, **82**(21):1879-1887.

643. Pavuluri MN, Passarotti AM, Fitzgerald JM, Wegbreit E, Sweeney JA: **Risperidone and divalproex differentially engage the fronto-striato-temporal circuitry in pediatric mania: a pharmacological functional magnetic resonance imaging study**. *Journal of the American Academy of Child and Adolescent Psychiatry* 2012, **51**(2):157-170.e155.

644. Perlstein WM, Carter CS, Noll DC, Cohen JD: **Relation of prefrontal cortex dysfunction to working memory and symptoms in schizophrenia**. *Am J Psychiatry* 2001, **158**(7):1105-1113.

645. Perlstein WM, Dixit NK, Carter CS, Noll DC, Cohen JD: **Prefrontal cortex dysfunction mediates deficits in working memory and prepotent responding in schizophrenia**. *Biol Psychiatry* 2003, **53**(1):25-38.

646. Perlstein WM, Cole MA, Demery JA, Seignourel PJ, Dixit NK, Larson MJ, Briggs RW: **Parametric manipulation of working memory load in traumatic brain injury: behavioral and neural correlates**. *Journal of the International Neuropsychological Society : JINS* 2004, **10**(5):724-741.

647. Pfefferbaum A, Desmond JE, Galloway C, Menon V, Glover GH, Sullivan EV: **Reorganization of frontal systems used by alcoholics for spatial working memory: an fMRI study**. *NeuroImage* 2001, **14**(1 Pt 1):7-20.

648. Philip NS, Sweet LH, Tyrka AR, Price LH, Carpenter LL, Kuras YI, Clark US, Niaura RS: **Early life stress is associated with greater default network deactivation during working memory in healthy controls: a preliminary report**. *Brain imaging and behavior* 2013, **7**(2):204-212.

649. Plichta MM, Schwarz AJ, Grimm O, Morgen K, Mier D, Haddad L, Gerdes AB, Sauer C, Tost H, Esslinger C *et al*: **Test-retest reliability of evoked BOLD signals from a cognitive-emotive fMRI test battery**. *NeuroImage* 2012, **60**(3):1746-1758.

650. Plichta MM, Grimm O, Morgen K, Mier D, Sauer C, Haddad L, Tost H, Esslinger C, Kirsch P, Schwarz AJ *et al*: **Amygdala habituation: a reliable fMRI phenotype**. *NeuroImage* 2014, **103**:383-390.

651. Pochon JB, Levy R, Fossati P, Lehericy S, Poline JB, Pillon B, Le Bihan D, Dubois B: **The neural system that bridges reward and cognition in humans: an fMRI study**. *Proceedings of the National Academy of Sciences of the United States of America* 2002, **99**(8):5669-5674.

652. Pomarol-Clotet E, Salvador R, Sarro S, Gomar J, Vila F, Martinez A, Guerrero A, Ortiz-Gil J, Sans-Sansa B, Capdevila A *et al*: **Failure to deactivate in the prefrontal cortex in schizophrenia: dysfunction of the default mode network?** *Psychol Med* 2008, **38**(8):1185-1193.

653. Pomarol-Clotet E, Moro N, Sarro S, Goikolea JM, Vieta E, Amann B, Fernandez-Corcuera P, Sans-Sansa B, Monte GC, Capdevila A *et al*: **Failure of de-activation in the medial frontal cortex in mania: evidence for default mode network dysfunction in the disorder**. *The world journal of biological psychiatry : the official journal of the World Federation of Societies of Biological Psychiatry* 2012, **13**(8):616-626.

654. Pomarol-Clotet E, Alonso-Lana S, Moro N, Sarro S, Bonnin MC, Goikolea JM, Fernandez-Corcuera P, Amann BL, Romaguera A, Vieta E *et al*: **Brain functional changes across the different phases of bipolar disorder**. *The British journal of psychiatry : the journal of mental science* 2015, **206**(2):136-144.

655. Poudel GR, Stout JC, Dominguez DJ, Gray MA, Salmon L, Churchyard A, Chua P, Borowsky B, Egan GF, Georgiou-Karistianis N: **Functional changes during working memory in Huntington's disease: 30-month longitudinal data from the IMAGE-HD study**. *Brain structure & function* 2015, **220**(1):501-512.

656. Prilipko O, Huynh N, Schwartz S, Tantrakul V, Kim JH, Peralta AR, Kushida C, Paiva T, Guilleminault C: **Task positive and default mode networks during a parametric working memory task in obstructive sleep apnea patients and healthy controls**. *Sleep* 2011, **34**(3):293-301a.

657. Prilipko O, Huynh N, Schwartz S, Tantrakul V, Kushida C, Paiva T, Guilleminault C: **The effects of CPAP treatment on task positive and default mode networks in obstructive sleep apnea patients: an fMRI study**. *PloS one* 2012, **7**(12):e47433.

658. Pu S, Yamada T, Yokoyama K, Matsumura H, Kobayashi H, Sasaki N, Mitani H, Adachi A, Kaneko K, Nakagome K: **A multi-channel near-infrared spectroscopy study of prefrontal cortex activation during working memory task in major depressive disorder**. *Neuroscience research* 2011, **70**(1):91-97.

659. Pyka M, Beckmann CF, Schoning S, Hauke S, Heider D, Kugel H, Arolt V, Konrad C: **Impact of working memory load on FMRI resting state pattern in subsequent resting phases**. *PloS one* 2009, **4**(9):e7198.

660. Pyka M, Hahn T, Heider D, Krug A, Sommer J, Kircher T, Jansen A: **Baseline activity predicts working memory load of preceding task condition**. *Human brain mapping* 2013, **34**(11):3010-3022.

661. Qin S, Hermans EJ, van Marle HJ, Luo J, Fernandez G: **Acute psychological stress reduces working memory-related activity in the dorsolateral prefrontal cortex**. *Biol Psychiatry* 2009, **66**(1):25-32.

662. Rama P, Martinkauppi S, Linnankoski I, Koivisto J, Aronen HJ, Carlson S: **Working memory of identification of emotional vocal expressions: an fMRI study**. *NeuroImage* 2001, **13**(6 Pt 1):1090-1101.

663. Rampino A, Walker RM, Torrance HS, Anderson SM, Fazio L, Di Giorgio A, Taurisano P, Gelao B, Romano R, Masellis R *et al*: **Expression of DISC1-interactome members correlates with cognitive phenotypes related to schizophrenia**. *PloS one* 2014, **9**(6):e99892.

664. Rasetti R, Mattay VS, Wiedholz LM, Kolachana BS, Hariri AR, Callicott JH, Meyer-Lindenberg A, Weinberger DR: **Evidence that altered amygdala activity in schizophrenia is related to clinical state and not genetic risk**. *Am J Psychiatry* 2009, **166**(2):216-225.

665. Richter S, Gorny X, Machts J, Behnisch G, Wustenberg T, Herbort MC, Munte TF, Seidenbecher CI, Schott BH: **Effects of AKAP5 Pro100Leu genotype on working memory for emotional stimuli**. *PloS one* 2013, **8**(1):e55613.

666. Rodriguez-Cano E, Sarro S, Monte GC, Maristany T, Salvador R, McKenna PJ, Pomarol-Clotet E: **Evidence for structural and functional abnormality in the subgenual anterior cingulate cortex in major depressive disorder**. *Psychol Med* 2014, **44**(15):3263-3273.

667. Rodriguez-Jimenez R, Avila C, Garcia-Navarro C, Bagney A, Aragon AM, Ventura-Campos N, Martinez-Gras I, Forn C, Ponce G, Rubio G *et al*: **Differential dorsolateral prefrontal cortex activation during a verbal n-back task according to sensory modality**. *Behavioural brain research* 2009, **205**(1):299-302.

668. Rose EJ, Simonotto E, Ebmeier KP: **Limbic over-activity in depression during preserved performance on the n-back task**. *NeuroImage* 2006, **29**(1):203-215.

669. Rose EJ, Simonotto E, Spencer EP, Ebmeier KP: **The effects of escitalopram on working memory and brain activity in healthy adults during performance of the n-back task**. *Psychopharmacology* 2006, **185**(3):339-347.

670. Roussotte FF, Bramen JE, Nunez SC, Quandt LC, Smith L, O'Connor MJ, Bookheimer SY, Sowell ER: **Abnormal brain activation during working memory in children with prenatal exposure to drugs of abuse: the effects of methamphetamine, alcohol, and polydrug exposure**. *NeuroImage* 2011, **54**(4):3067-3075.

671. Royer A, Schneider FC, Grosselin A, Pellet J, Barral FG, Laurent B, Brouillet D, Lang F: **Brain activation during executive processes in schizophrenia**. *Psychiatry research* 2009, **173**(3):170-176.

672. Rudner M, Fransson P, Ingvar M, Nyberg L, Ronnberg J: **Neural representation of binding lexical signs and words in the episodic buffer of working memory**. *Neuropsychologia* 2007, **45**(10):2258-2276.

673. Rzucidlo JK, Roseman PL, Laurienti PJ, Dagenbach D: **Stability of whole brain and regional network topology within and between resting and cognitive states**. *PloS one* 2013, **8**(8):e70275.

674. Sabri O, Owega A, Schreckenberger M, Sturz L, Fimm B, Kunert P, Meyer PT, Sander D, Klingelhofer J: **A truly simultaneous combination of functional transcranial Doppler sonography and H(2)(15)O PET adds fundamental new information on differences in cognitive activation between schizophrenics and healthy control subjects**. *Journal of nuclear medicine : official publication, Society of Nuclear Medicine* 2003, **44**(5):671-681.

675. Sabri M, Humphries C, Verber M, Liebenthal E, Binder JR, Mangalathu J, Desai A: **Neural effects of cognitive control load on auditory selective attention**. *Neuropsychologia* 2014, **61**:269-279.

676. Sala-Llonch R, Pena-Gomez C, Arenaza-Urquijo EM, Vidal-Pineiro D, Bargallo N, Junque C, Bartres-Faz D: **Brain connectivity during resting state and subsequent working memory task predicts behavioural performance**. *Cortex; a journal devoted to the study of the nervous system and behavior* 2012, **48**(9):1187-1196.

677. Saliasi E, Geerligs L, Lorist MM, Maurits NM: **Neural correlates associated with successful working memory performance in older adults as revealed by spatial ICA**. *PloS one* 2014, **9**(6):e99250.

678. Salvador R, Martinez A, Pomarol-Clotet E, Gomar J, Vila F, Sarro S, Capdevila A, Bullmore E: **A simple view of the brain through a frequency-specific functional connectivity measure**. *NeuroImage* 2008, **39**(1):279-289.

679. Sambataro F, Blasi G, Fazio L, Caforio G, Taurisano P, Romano R, Di Giorgio A, Gelao B, Lo Bianco L, Papazacharias A *et al*: **Treatment with olanzapine is associated with modulation of the default mode network in patients with Schizophrenia**. *Neuropsychopharmacology : official publication of the American College of Neuropsychopharmacology* 2010, **35**(4):904-912.

680. Sanchez-Carrion R, Fernandez-Espejo D, Junque C, Falcon C, Bargallo N, Roig T, Bernabeu M, Tormos JM, Vendrell P: **A longitudinal fMRI study of working memory in severe TBI patients with diffuse axonal injury**. *NeuroImage* 2008, **43**(3):421-429.

681. Sandstrom A, Sall R, Peterson J, Salami A, Larsson A, Olsson T, Nyberg L: **Brain activation patterns in major depressive disorder and work stress-related long-term sick leave among Swedish females**. *Stress (Amsterdam, Netherlands)* 2012, **15**(5):503-513.

682. Sapara A, Ffytche DH, Birchwood M, Cooke MA, Fannon D, Williams SC, Kuipers E, Kumari V: **Preservation and compensation: the functional neuroanatomy of insight and working memory in schizophrenia**. *Schizophr Res* 2014, **152**(1):201-209.

683. Satterthwaite TD, Ruparel K, Loughead J, Elliott MA, Gerraty RT, Calkins ME, Hakonarson H, Gur RC, Gur RE, Wolf DH: **Being right is its own reward: load and performance related ventral striatum activation to correct responses during a working memory task in youth**. *NeuroImage* 2012, **61**(3):723-729.

684. Savini N, Brunetti M, Babiloni C, Ferretti A: **Working memory of somatosensory stimuli: an fMRI study**. *International journal of psychophysiology : official journal of the International Organization of Psychophysiology* 2012, **86**(3):220-228.

685. Scheibel RS, Pearson DA, Faria LP, Kotrla KJ, Aylward E, Bachevalier J, Levin HS: **An fMRI study of executive functioning after severe diffuse TBI**. *Brain injury* 2003, **17**(11):919-930.

686. Schlagenhauf F, Dinges M, Beck A, Wustenberg T, Friedel E, Dembler T, Sarkar R, Wrase J, Gallinat J, Juckel G *et al*: **Switching schizophrenia patients from typical neuroleptics to aripiprazole: effects on working memory dependent functional activation**. *Schizophr Res* 2010, **118**(1-3):189-200.

687. Schlosser R, Gesierich T, Kaufmann B, Vucurevic G, Hunsche S, Gawehn J, Stoeter P: **Altered effective connectivity during working memory performance in schizophrenia: a study with fMRI and structural equation modeling**. *NeuroImage* 2003, **19**(3):751-763.

688. Schlosser R, Gesierich T, Kaufmann B, Vucurevic G, Stoeter P: **Altered effective connectivity in drug free schizophrenic patients**. *Neuroreport* 2003, **14**(17):2233-2237.

689. Schlosser RG, Gesierich T, Wagner G, Bolz M, Grunder G, Dielentheis TF, Scherb C, Stoeter P: **Altered benzodiazepine receptor sensitivity in alcoholism: a study with fMRI and acute lorazepam challenge**. *Psychiatry research* 2007, **154**(3):241-251.

690. Schmidt A, Smieskova R, Aston J, Simon A, Allen P, Fusar-Poli P, McGuire PK, Riecher-Rossler A, Stephan KE, Borgwardt S: **Brain connectivity abnormalities predating the onset of psychosis: correlation with the effect of medication**. *JAMA psychiatry* 2013, **70**(9):903-912.

691. Schmidt A, Smieskova R, Simon A, Allen P, Fusar-Poli P, McGuire PK, Bendfeldt K, Aston J, Lang UE, Walter M *et al*: **Abnormal effective connectivity and psychopathological symptoms in the psychosis high-risk state**. *Journal of psychiatry & neuroscience : JPN* 2014, **39**(4):239-248.

692. Schoning S, Zwitserlood P, Engelien A, Behnken A, Kugel H, Schiffbauer H, Lipina K, Pachur C, Kersting A, Dannlowski U *et al*: **Working-memory fMRI reveals cingulate hyperactivation in euthymic major depression**. *Human brain mapping* 2009, **30**(9):2746-2756.

693. Seidman LJ, Thermenos HW, Poldrack RA, Peace NK, Koch JK, Faraone SV, Tsuang MT: **Altered brain activation in dorsolateral prefrontal cortex in adolescents and young adults at genetic risk for schizophrenia: an fMRI study of working memory**. *Schizophr Res* 2006, **85**(1-3):58-72.

694. Seidman LJ, Rosso IM, Thermenos HW, Makris N, Juelich R, Gabrieli JD, Faraone SV, Tsuang MT, Whitfield-Gabrieli S: **Medial temporal lobe default mode functioning and hippocampal structure as vulnerability indicators for schizophrenia: a MRI study of non-psychotic adolescent first-degree relatives**. *Schizophr Res* 2014, **159**(2-3):426-434.

695. Smieskova R, Allen P, Simon A, Aston J, Bendfeldt K, Drewe J, Gruber K, Gschwandtner U, Klarhoefer M, Lenz C *et al*: **Different duration of at-risk mental state associated with neurofunctional abnormalities. A multimodal imaging study**. *Human brain mapping* 2012, **33**(10):2281-2294.

696. Smith AM, Fried PA, Hogan MJ, Cameron I: **Effects of prenatal marijuana on visuospatial working memory: an fMRI study in young adults**. *Neurotoxicology and teratology* 2006, **28**(2):286-295.

697. Smith AM, Longo CA, Fried PA, Hogan MJ, Cameron I: **Effects of marijuana on visuospatial working memory: an fMRI study in young adults**. *Psychopharmacology* 2010, **210**(3):429-438.

698. Smits M, Dippel DW, Houston GC, Wielopolski PA, Koudstaal PJ, Hunink MG, van der Lugt A: **Postconcussion syndrome after minor head injury: brain activation of working memory and attention**. *Human brain mapping* 2009, **30**(9):2789-2803.

699. Spadoni AD, Bazinet AD, Fryer SL, Tapert SF, Mattson SN, Riley EP: **BOLD response during spatial working memory in youth with heavy prenatal alcohol exposure**. *Alcoholism, clinical and experimental research* 2009, **33**(12):2067-2076.

700. Spreng RN, DuPre E, Selarka D, Garcia J, Gojkovic S, Mildner J, Luh WM, Turner GR: **Goal-congruent default network activity facilitates cognitive control**. *The Journal of neuroscience : the official journal of the Society for Neuroscience* 2014, **34**(42):14108-14114.

701. Stingl JC, Esslinger C, Tost H, Bilek E, Kirsch P, Ohmle B, Viviani R, Walter H, Rietschel M, Meyer-Lindenberg A: **Genetic variation in CYP2D6 impacts neural activation during cognitive tasks in humans**. *NeuroImage* 2012, **59**(3):2818-2823.

702. Stokes PR, Rhodes RA, Grasby PM, Mehta MA: **The effects of the COMT Val108/158Met polymorphism on BOLD activation during working memory, planning, and response inhibition: a role for the posterior cingulate cortex?** *Neuropsychopharmacology : official publication of the American College of Neuropsychopharmacology* 2011, **36**(4):763-771.

703. Stollstorff M, Foss-Feig J, Cook EH, Jr., Stein MA, Gaillard WD, Vaidya CJ: **Neural response to working memory load varies by dopamine transporter genotype in children**. *NeuroImage* 2010, **53**(3):970-977.

704. Stoodley CJ, Valera EM, Schmahmann JD: **An fMRI study of intra-individual functional topography in the human cerebellum**. *Behavioural neurology* 2010, **23**(1-2):65-79.

705. Subramaniam K, Luks TL, Garrett C, Chung C, Fisher M, Nagarajan S, Vinogradov S: **Intensive cognitive training in schizophrenia enhances working memory and associated prefrontal cortical efficiency in a manner that drives long-term functional gains**. *NeuroImage* 2014, **99**:281-292.

706. Sugranyes G, Kyriakopoulos M, Dima D, O'Muircheartaigh J, Corrigall R, Pendelbury G, Hayes D, Calhoun VD, Frangou S: **Multimodal analyses identify linked functional and white matter abnormalities within the working memory network in schizophrenia**. *Schizophr Res* 2012, **138**(2-3):136-142.

707. Sumowski JF, Wylie GR, Deluca J, Chiaravalloti N: **Intellectual enrichment is linked to cerebral efficiency in multiple sclerosis: functional magnetic resonance imaging evidence for cognitive reserve**. *Brain* 2010, **133**(Pt 2):362-374.

708. Sundermann EE, Bishop JR, Rubin LH, Little DM, Meyer VJ, Martin E, Weber K, Cohen M, Maki PM: **Genetic predictor of working memory and prefrontal function in women with HIV**. *Journal of neurovirology* 2015, **21**(1):81-91.

709. Surguladze SA, Chu EM, Evans A, Anilkumar AP, Patel MX, Timehin C, David AS: **The effect of long-acting risperidone on working memory in schizophrenia: a functional magnetic resonance imaging study**. *Journal of clinical psychopharmacology* 2007, **27**(6):560-570.

710. Sweet LH, Rao SM, Primeau M, Mayer AR, Cohen RA: **Functional magnetic resonance imaging of working memory among multiple sclerosis patients**. *Journal of neuroimaging : official journal of the American Society of Neuroimaging* 2004, **14**(2):150-157.

711. Sweet LH, Rao SM, Primeau M, Durgerian S, Cohen RA: **Functional magnetic resonance imaging response to increased verbal working memory demands among patients with multiple sclerosis**. *Human brain mapping* 2006, **27**(1):28-36.

712. Sweet LH, Paskavitz JF, Haley AP, Gunstad JJ, Mulligan RC, Nyalakanti PK, Cohen RA: **Imaging phonological similarity effects on verbal working memory**. *Neuropsychologia* 2008, **46**(4):1114-1123.

713. Sweet LH, Jerskey BA, Aloia MS: **Default network response to a working memory challenge after withdrawal of continuous positive airway pressure treatment for obstructive sleep apnea**. *Brain imaging and behavior* 2010, **4**(2):155-163.

714. Szatkowska I, Bogorodzki P, Wolak T, Marchewka A, Szeszkowski W: **The effect of motivation on working memory: an fMRI and SEM study**. *Neurobiology of learning and memory* 2008, **90**(2):475-478.

715. Takeuchi H, Taki Y, Hashizume H, Sassa Y, Nagase T, Nouchi R, Kawashima R: **Effects of training of processing speed on neural systems**. *The Journal of neuroscience : the official journal of the Society for Neuroscience* 2011, **31**(34):12139-12148.

716. Takeuchi H, Taki Y, Hashizume H, Sassa Y, Nagase T, Nouchi R, Kawashima R: **Failing to deactivate: the association between brain activity during a working memory task and creativity**. *NeuroImage* 2011, **55**(2):681-687.

717. Takeuchi H, Sugiura M, Sassa Y, Sekiguchi A, Yomogida Y, Taki Y, Kawashima R: **Neural correlates of the difference between working memory speed and simple sensorimotor speed: an fMRI study**. *PloS one* 2012, **7**(1):e30579.

718. Takeuchi H, Taki Y, Nouchi R, Hashizume H, Sassa Y, Sekuguchi A, Kotozaki Y, Nakagawa S, Nagase T, Miyauchi CM *et al*: **Associations among imaging measures (2): the association between gray matter concentration and task-induced activation changes**. *Human brain mapping* 2014, **35**(1):185-198.

719. Tan HY, Sust S, Buckholtz JW, Mattay VS, Meyer-Lindenberg A, Egan MF, Weinberger DR, Callicott JH: **Dysfunctional prefrontal regional specialization and compensation in schizophrenia**. *Am J Psychiatry* 2006, **163**(11):1969-1977.

720. Tang CY, Carpenter DM, Eaves EL, Ng J, Ganeshalingam N, Weisel C, Qian H, Lange G, Fiedler NL: **Occupational solvent exposure and brain function: an fMRI study**. *Environmental health perspectives* 2011, **119**(7):908-913.

721. Teipel S, Ehlers I, Erbe A, Holzmann C, Lau E, Hauenstein K, Berger C: **Structural Connectivity Changes Underlying Altered Working Memory Networks in Mild Cognitive Impairment: A Three-Way Image Fusion Analysis**. *Journal of neuroimaging : official journal of the American Society of Neuroimaging* 2015, **25**(4):634-642.

722. Thermenos HW, Goldstein JM, Buka SL, Poldrack RA, Koch JK, Tsuang MT, Seidman LJ: **The effect of working memory performance on functional MRI in schizophrenia**. *Schizophr Res* 2005, **74**(2-3):179-194.

723. Thermenos HW, Goldstein JM, Milanovic SM, Whitfield-Gabrieli S, Makris N, Laviolette P, Koch JK, Faraone SV, Tsuang MT, Buka SL *et al*: **An fMRI study of working memory in persons with bipolar disorder or at genetic risk for bipolar disorder**. *American journal of medical genetics Part B, Neuropsychiatric genetics : the official publication of the International Society of Psychiatric Genetics* 2010, **153b**(1):120-131.

724. Thermenos HW, Makris N, Whitfield-Gabrieli S, Brown AB, Giuliano AJ, Lee EH, Faraone SV, Tsuang MT, Seidman LJ: **A functional MRI study of working memory in adolescents and young adults at genetic risk for bipolar disorder: preliminary findings**. *Bipolar Disord* 2011, **13**(3):272-286.

725. Thomas RJ: **Fatigue in the executive cortical network demonstrated in narcoleptics using functional magnetic resonance imaging--a preliminary study**. *Sleep medicine* 2005, **6**(5):399-406.

726. Thomas RJ, Kwong K: **Modafinil activates cortical and subcortical sites in the sleep-deprived state**. *Sleep* 2006, **29**(11):1471-1481.

727. Thormodsen R, Jensen J, Holmen A, Juuhl-Langseth M, Emblem KE, Andreassen OA, Rund BR: **Prefrontal hyperactivation during a working memory task in early-onset schizophrenia spectrum disorders: an fMRI study**. *Psychiatry research* 2011, **194**(3):257-262.

728. Thornton MA, Conway AR: **Working memory for social information: chunking or domain-specific buffer?** *NeuroImage* 2013, **70**:233-239.

729. Thurling M, Hautzel H, Kuper M, Stefanescu MR, Maderwald S, Ladd ME, Timmann D: **Involvement of the cerebellar cortex and nuclei in verbal and visuospatial working memory: a 7 T fMRI study**. *NeuroImage* 2012, **62**(3):1537-1550.

730. Tomasi DG, Caparelli EC: **Macrovascular contribution in activation patterns of working memory**. *Journal of cerebral blood flow and metabolism : official journal of the International Society of Cerebral Blood Flow and Metabolism* 2007, **27**(1):33-42.

731. Tu PC, Hsieh JC, Li CT, Bai YM, Su TP: **Cortico-striatal disconnection within the cingulo-opercular network in schizophrenia revealed by intrinsic functional connectivity analysis: a resting fMRI study**. *NeuroImage* 2012, **59**(1):238-247.

732. Valera EM, Faraone SV, Biederman J, Poldrack RA, Seidman LJ: **Functional neuroanatomy of working memory in adults with attention-deficit/hyperactivity disorder**. *Biol Psychiatry* 2005, **57**(5):439-447.

733. Valera EM, Brown A, Biederman J, Faraone SV, Makris N, Monuteaux MC, Whitfield-Gabrieli S, Vitulano M, Schiller M, Seidman LJ: **Sex differences in the functional neuroanatomy of working memory in adults with ADHD**. *Am J Psychiatry* 2010, **167**(1):86-94.

734. van Ast VA, Spicer J, Smith EE, Schmer-Galunder S, Liberzon I, Abelson JL, Wager TD: **Brain Mechanisms of Social Threat Effects on Working Memory**. *Cerebral cortex (New York, NY : 1991)* 2016, **26**(2):544-556.

735. van der Wee NJ, Ramsey NF, van Megen HJ, Denys D, Westenberg HG, Kahn RS: **Spatial working memory in obsessive-compulsive disorder improves with clinical response: A functional MRI study**. *European neuropsychopharmacology : the journal of the European College of Neuropsychopharmacology* 2007, **17**(1):16-23.

736. Venneri A, McGeown WJ, Shanks MF: **Responders to ChEI treatment of Alzheimer's disease show restitution of normal regional cortical activation**. *Current Alzheimer research* 2009, **6**(2):97-111.

737. Vermeij A, van Beek AH, Olde Rikkert MG, Claassen JA, Kessels RP: **Effects of aging on cerebral oxygenation during working-memory performance: a functional near-infrared spectroscopy study**. *PloS one* 2012, **7**(9):e46210.

738. Voss B, Thienel R, Reske M, Kellermann T, Sheldrick AJ, Halfter S, Radenbach K, Shah NJ, Habel U, Kircher TT: **Cholinergic blockade under working memory demands encountered by increased rehearsal strategies: evidence from fMRI in healthy subjects**. *Eur Arch Psychiatry Clin Neurosci* 2012, **262**(4):329-339.

739. Vu MA, Thermenos HW, Terry DP, Wolfe DJ, Voglmaier MM, Niznikiewicz MA, McCarley RW, Seidman LJ, Dickey CC: **Working memory in schizotypal personality disorder: fMRI activation and deactivation differences**. *Schizophr Res* 2013, **151**(1-3):113-123.

740. Vuontela V, Steenari MR, Aronen ET, Korvenoja A, Aronen HJ, Carlson S: **Brain activation and deactivation during location and color working memory tasks in 11-13-year-old children**. *Brain and cognition* 2009, **69**(1):56-64.

741. Walsh ND, Williams SC, Brammer MJ, Bullmore ET, Kim J, Suckling J, Mitterschiffthaler MT, Cleare AJ, Pich EM, Mehta MA *et al*: **A longitudinal functional magnetic resonance imaging study of verbal working memory in depression after antidepressant therapy**. *Biol Psychiatry* 2007, **62**(11):1236-1243.

742. Walter H, Wunderlich AP, Blankenhorn M, Schafer S, Tomczak R, Spitzer M, Gron G: **No hypofrontality, but absence of prefrontal lateralization comparing verbal and spatial working memory in schizophrenia**. *Schizophr Res* 2003, **61**(2-3):175-184.

743. Wardle MC, de Wit H, Penton-Voak I, Lewis G, Munafo MR: **Lack of association between COMT and working memory in a population-based cohort of healthy young adults**. *Neuropsychopharmacology : official publication of the American College of Neuropsychopharmacology* 2013, **38**(7):1253-1263.

744. Wei X, Yoo SS, Dickey CC, Zou KH, Guttmann CR, Panych LP: **Functional MRI of auditory verbal working memory: long-term reproducibility analysis**. *NeuroImage* 2004, **21**(3):1000-1008.

745. Weiland BJ, Nigg JT, Welsh RC, Yau WY, Zubieta JK, Zucker RA, Heitzeg MM: **Resiliency in adolescents at high risk for substance abuse: flexible adaptation via subthalamic nucleus and linkage to drinking and drug use in early adulthood**. *Alcoholism, clinical and experimental research* 2012, **36**(8):1355-1364.

746. Winston GP, Stretton J, Sidhu MK, Symms MR, Thompson PJ, Duncan JS: **Structural correlates of impaired working memory in hippocampal sclerosis**. *Epilepsia* 2013, **54**(7):1143-1153.

747. Wishart HA, Saykin AJ, McDonald BC, Mamourian AC, Flashman LA, Schuschu KR, Ryan KA, Fadul CE, Kasper LH: **Brain activation patterns associated with working memory in relapsing-remitting MS**. *Neurology* 2004, **62**(2):234-238.

748. Wishart HA, Saykin AJ, Rabin LA, Santulli RB, Flashman LA, Guerin SJ, Mamourian AC, Belloni DR, Rhodes CH, McAllister TW: **Increased brain activation during working memory in cognitively intact adults with the APOE epsilon4 allele**. *Am J Psychiatry* 2006, **163**(9):1603-1610.

749. Wolfe KR, Madan-Swain A, Hunter GR, Reddy AT, Banos J, Kana RK: **An fMRI investigation of working memory and its relationship with cardiorespiratory fitness in pediatric posterior fossa tumor survivors who received cranial radiation therapy**. *Pediatric blood & cancer* 2013, **60**(4):669-675.

750. Wylie GR, Genova H, DeLuca J, Chiaravalloti N, Sumowski JF: **Functional magnetic resonance imaging movers and shakers: does subject-movement cause sampling bias?** *Human brain mapping* 2014, **35**(1):1-13.

751. Xu J, Mendrek A, Cohen MS, Monterosso J, Rodriguez P, Simon SL, Brody A, Jarvik M, Domier CP, Olmstead R *et al*: **Brain activity in cigarette smokers performing a working memory task: effect of smoking abstinence**. *Biol Psychiatry* 2005, **58**(2):143-150.

752. Xu J, Mendrek A, Cohen MS, Monterosso J, Simon S, Brody AL, Jarvik M, Rodriguez P, Ernst M, London ED: **Effects of acute smoking on brain activity vary with abstinence in smokers performing the N-Back task: a preliminary study**. *Psychiatry research* 2006, **148**(2-3):103-109.

753. Yan X, Zhang J, Gong Q, Weng X: **Prolonged high-altitude residence impacts verbal working memory: an fMRI study**. *Experimental brain research* 2011, **208**(3):437-445.

754. Yin JJ, Liao LM, Luo DX, Xu K, Ma SH, Wang ZX, Le HB, Huang RR, Cai ZL, Zhang J: **Spatial working memory impairment in subclinical hypothyroidism: an FMRI study**. *Neuroendocrinology* 2013, **97**(3):260-270.

755. Yoo SS, Choi BG, Juh RH, Park JM, Pae CU, Kim JJ, Lee SJ, Lee C, Paik IH, Lee CU: **Working memory processing of facial images in schizophrenia: fMRI investigation**. *Int J Neurosci* 2005, **115**(3):351-366.

756. Yu B, Guo Q, Fan G, Ma H, Wang L, Liu N: **Evaluation of working memory impairment in children with primary nocturnal enuresis: evidence from event-related functional magnetic resonance imaging**. *Journal of paediatrics and child health* 2011, **47**(7):429-435.

757. Zhang J, Wang Z, Xu S, Chen Y, Chen K, Liu L, Wang Y, Guo R, Zhang Z: **The effects of CCRC on cognition and brain activity in aMCI patients: a pilot placebo controlled BOLD fMRI study**. *Current Alzheimer research* 2014, **11**(5):484-493.

758. Zhou Y, Wang Z, Zuo XN, Zhang H, Wang Y, Jiang T, Liu Z: **Hyper-coupling between working memory task-evoked activations and amplitude of spontaneous fluctuations in first-episode schizophrenia**. *Schizophr Res* 2014, **159**(1):80-89.

759. Zhu DF, Wang ZX, Zhang DR, Pan ZL, He S, Hu XP, Chen XC, Zhou JN: **fMRI revealed neural substrate for reversible working memory dysfunction in subclinical hypothyroidism**. *Brain* 2006, **129**(Pt 11):2923-2930.

760. Ziemus B, Baumann O, Luerding R, Schlosser R, Schuierer G, Bogdahn U, Greenlee MW: **Impaired working-memory after cerebellar infarcts paralleled by changes in BOLD signal of a cortico-cerebellar circuit**. *Neuropsychologia* 2007, **45**(9):2016-2024.

761. Zou Q, Ross TJ, Gu H, Geng X, Zuo XN, Hong LE, Gao JH, Stein EA, Zang YF, Yang Y: **Intrinsic resting-state activity predicts working memory brain activation and behavioral performance**. *Human brain mapping* 2013, **34**(12):3204-3215.
