# Supplementary material for: Does emotion regulation engage the same neural circuit as working memory? A meta-analytical comparison between cognitive reappraisal of negative emotion and 2-back working memory task
Source: PLoS One. 2018 Sep 13;13(9):e0203753. doi: 10.1371/journal.pone.0203753 (PMC6136767; doi:10.1371/journal.pone.0203753)
Supplement: S3 File — (DOCX) [file pone.0203753.s003.docx]

**PRISMA-IPD Checklist of items to include when reporting a systematic review and meta-analysis of individual participant data (IPD)**

| **PRISMA-IPD**  **Section/topic** | **Item No** | **Checklist item** | **Reported on page** |
| --- | --- | --- | --- |
| **Title** | | | |
| Title | 1 | P1. Does emotion regulation engage the same neural circuit as working memory? A meta-analytical comparison between cognitive reappraisal of negative emotion and 2-back working memory task |  |
| **Abstract** | | | |
| Structured summary | 2 | P3. Structured summary is provided in **Abstract** of the submitted manuscript. |  |
| **Introduction** | | | |
| Rationale | 3 | P5-6. Rational is provided in **Introduction** of the submitted manuscript. |  |
| Objectives | 4 | P8. Objectives are provided in **Introduction** of the submitted manuscript. |  |
| **Methods** | | | |
| Protocol and registration | 5 | N/A |  |
| Eligibility criteria | 6 | P9, P12. Eligibility criteria are provided in **Methods** of the submitted manuscript. |  |
| Identifying studies - information sources | 7 | P10, P12. The information source is from PubMed. Since the study numbers of ER and WM both are approximately 50, the power is enough in terms of a meta-analysis. |  |
| Identifying studies - search | 8 | P10, P12. Search strategies of ER and WM are provided in **Methods** of the submitted manuscript. |  |
| Study selection processes | 9 | P10, P12. Study selection processes are provided in **Methods** of the submitted manuscript. |  |
| Data collection processes | 10 | P10, P13. Data collection processes are detailed in **Supplementary Material** and are summarized in **Methods** of the submitted manuscript. |  |
| Data items | 11 | P11, P14. Data items are the Talairach coordinates. |  |
| IPD integrity | A1 | N/A |  |
| Risk of bias assessment in individual studies. | 12 | P14. The meta-analytic tool (GingerALE) may identify the data coordinates that are out of the brain. |  |
| Specification of outcomes and effect measures | 13 | N/A |  |
| Synthesis methods | 14 | P14, P15. This meta-analysis used broadly-acknowledged meta-analytic tool GingerALE software and applied cluster-level inferences according to an updated report (one of the most rigorous and reliable methods for the modelling of peak coordinates across neuroimaging studies). |  |
| Exploration of variation in effects | A2 | N/A |  |
| Risk of bias across studies | 15 | P13 and **Supplementary Material**. This meta-analysis is based on an exhaustive survey, and around 1000 articles for 10+ years were assessed. Later studies may only report between condition/group comparisons, which may constitute a potential bias due to time. This point has been explained in the **Methods.** |  |
| Additional analyses | 16 | P15. In addition to the main effects, contrast and conjunction analyses were also performed. |  |
| **Results** | | | |
| Study selection and IPD obtained | 17 | P11, P12. The numbers of screened, excluded and included studies screened are detailed in **Results** and in **Supplementary Material**. A flow diagram (Figure 1) is included. |  |
| Study characteristics | 18 | P15. This meta-analysis is based on brain imaging findings of healthy subjects. |  |
| IPD integrity | A3 | N/A |  |
| Risk of bias within studies | 19 | P15. The meta-analytic tool (GingerALE) may identify the data coordinates that are out of the brain. |  |
| Results of individual studies | 20 | P48-53. The detailed of each of the included studies are summarized in **Tables 1 and 2**. |  |
| Results of syntheses | 21 | P15-17. The reported summary effects for each meta-analysis undertaken conform to the state-of-art mea-analytic convention of neuroimaging research. |  |
| Risk of bias across studies | 22 | P15 and **Supplementary Material**. This meta-analysis is based on an exhaustive survey, and around 1000 articles for 10+ years were assessed. Later studies may only report between condition/group comparisons, which may constitute a potential bias due to time. This point has been explained in the **Methods.** |  |
| Additional analyses | 23 | P17 and **Supplementary Material**. In addition to the main effects, contrast and conjunction analyses were also performed. The results are illustrated in Figures and summarized in Tables of **Supplementary Material**. |  |
| **Discussion** | | | |
| Summary of evidence | 24 | P17-P24. The main findings are summarized in **Discussion**. |  |
| Strengths and limitations | 25 | P17, P25. Important strengths are provided in **Discussion**. In addition, there is a separate section **Limitation**. |  |
| Conclusions | 26 | P27. A general interpretation of the findings is provided in **Conclusion**. |  |
| Implications | A4 | P24. The potential theoretical and clinical implications are provided in **Discussion**. |  |
| **Funding** | | | |
| Funding | 27 | P29. The funding sources are provided in **Acknowledgements**. |  |

**A1 – A3 denote new items that are additional to standard PRISMA items. A4 has been created as a result of re-arranging content of the standard PRISMA statement to suit the way that systematic review IPD meta-analyses are reported.**

© Reproduced with permission of the PRISMA IPD Group, which encourages sharing and reuse for non-commercial purposes
